# Staphylococcal Bap Proteins Build Amyloid Scaffold Biofilm Matrices in Response to Environmental Signals

**DOI:** 10.1371/journal.ppat.1005711

**Published:** 2016-06-21

**Authors:** Agustina Taglialegna, Susanna Navarro, Salvador Ventura, James A. Garnett, Steve Matthews, José R. Penades, Iñigo Lasa, Jaione Valle

**Affiliations:** 1 Laboratory of Microbial Biofilms, Idab-CSIC-Universidad Pública de Navarra-Gobierno de Navarra and Dpto Producción Agraria, Pamplona, Spain; 2 Institut de Biotecnologia i de Biomedicina and Departament de Bioquimica i Biologia Molecular, Universitat Autonoma de Barcelona, Bellaterra, Spain; 3 School of Biological and Chemical Sciences, Queen Mary University of London, London, United Kingdom; 4 Department of Life Sciences, Imperial College London, London, United Kingdom; 5 Institute of Infection, Immunity and Inflammation, College of Medical, Veterinary and Life Sciences, University of Glasgow, Glasgow, United Kingdom; 6 Navarrabiomed-Universidad Pública de Navarra, IdiSNA, Pamplona, Spain; University of Washington, UNITED STATES

## Abstract

Biofilms are communities of bacteria that grow encased in an extracellular matrix that often contains proteins. The spatial organization and the molecular interactions between matrix scaffold proteins remain in most cases largely unknown. Here, we report that Bap protein of *Staphylococcus aureus* self-assembles into functional amyloid aggregates to build the biofilm matrix in response to environmental conditions. Specifically, Bap is processed and fragments containing at least the N-terminus of the protein become aggregation-prone and self-assemble into amyloid-like structures under acidic pHs and low concentrations of calcium. The molten globule-like state of Bap fragments is stabilized upon binding of the cation, hindering its self-assembly into amyloid fibers. These findings define a dual function for Bap, first as a sensor and then as a scaffold protein to promote biofilm development under specific environmental conditions. Since the pH-driven multicellular behavior mediated by Bap occurs in coagulase-negative staphylococci and many other bacteria exploit Bap-like proteins to build a biofilm matrix, the mechanism of amyloid-like aggregation described here may be widespread among pathogenic bacteria.

## Author Summary

Major components of the biofilm matrix scaffold are proteins that assemble to create a unified structure that maintain bacteria attached to each other and to surfaces. We provide evidence that a surface protein present in several staphylococcal species forms functional amyloid aggregates to build the biofilm matrix in response to specific environmental conditions. Under low Ca^2+^ concentrations and acidic pH, Bap is processed and forms insoluble aggregates with amyloidogenic properties. When the Ca^2+^ concentration increases, metal-coordinated Bap adopts a structurally more stable conformation and as a consequence, the N-terminal region is unable to assemble into amyloid aggregates. The control of Bap cleavage and assembly helps to regulate biofilm matrix development as a function of environmental changes.

## Introduction

Biofilm formation is universal for all bacteria. The molecular mechanisms governing this process vary among bacteria, but they all culminate in the synthesis of an extracellular matrix. The composition of the extracellular matrix is complex and variable, even within the same bacterial species when environmental conditions are altered [1,2]. However, one common principle is that the matrix scaffold is built from exopolysaccharide or proteins, which eventually can be interwoven with extracellular genomic DNA [3–5]. The reasons underlying the election of a polysaccharide or protein-based biofilm matrix are not well understood, but an increasing number of studies indicate that proteinaceous scaffolds are more common than previously anticipated. Proteins anchored to the bacterial cell surface can assemble the matrix scaffold through homophilic interactions between identical molecules expressed on neighboring cells or through heterophilic interactions with other surface proteins or with non-proteinaceous cell wall structures [6,7]. Members of this group of proteins include autotransporter adhesins [8–11], carbohydrate-binding proteins [12–14], and cell-wall anchored proteins covalently linked to the peptidoglycan (CWA) [2,15–21]. Another strategy by which proteins can contribute to the formation of the matrix scaffold is through polymerization into functional amyloid fibers. Secreted proteins can assemble to form insoluble fibers with a characteristic cross-β-strand structure, where the β-sheets run perpendicular to the fibril axis [22]. Once polymerized, amyloid fibers constitute a strong platform able to mediate interactions between the neighboring cells and surfaces [23–26]. Examples of amyloid fibers mediating biofilm development include curli pili present in *Enterobacteriaceae* [27,28], FapC in *Pseudomonas fluorescens* [29], TasA in *Bacillus subtilis* [30], the aggregative flexible pili named MTP in the pathogen *Mycobacterium tuberculosis* [31,32] and phenol soluble modulins (PSMs) in *Staphylococcus aureus* [33].

Biofilm associated proteins (Bap) are high molecular weight multi-domain proteins, characterized by a repetitive structure and localized at the cell surface [34]. The first member of this family of proteins was identified in a mobile pathogenicity island (SaPIbov2) present in some strains of *S*. *aureus*. So far, the *bap* gene has been identified in mastitis-derived staphylococcal species, but has never been found in *S*. *aureus* human isolates. However, *bap* orthologous genes are present in the core genome of several coagulase-negative staphylococcal species that belong to the human commensal microbiota such as *S*. *saprophyticus* ATCC15305 (Accession number GCA_000010125.1), *S*. *epidermidis* (GCA_000759555.1) and *S*. *warneri* SG1 (GCA_000332735.1) [35]. Bap promotes the initial attachment to inert surfaces and cell-to-cell interactions through a mechanism that is independent of exopolysaccharide [21,36]. During infection, Bap facilitates the persistence in the mammary gland by enhancing adhesion to epithelial cells and prevents cellular internalization through the binding to GP96 host receptor, which interferes with the FnBPs mediated invasion pathway [37,38]. Overall these results indicated that Bap plays a dual function: on the one hand, mediating bacterial-bacterial interactions and on the other, bacterial-host interactions. However, the molecular mechanisms by which Bap performs these functions and the region of the protein involved in each process remain unexplored.

In this report, we investigated the mechanistic basis by which Bap proteins promote the formation of the biofilm matrix scaffold. Our results have shown that Bap is constitutively expressed along the growth curve and processed. The resulting fragments, which likely contain mainly the N-terminal region of the protein, form insoluble amyloid–like aggregates when the pH of the media becomes acidic and the concentration of calcium is low. If calcium concentration increases, metal-coordinated Bap adopts a more stable conformation as shown by thermal denaturation monitored by intrinsic fluorescence, nuclear magnetic resonance (NMR), proteinase K digestion and analytical ultracentrifugation. As a consequence, the N-terminal region is unable to self-assemble and to mediate intercellular aggregation and biofilm formation. Furthermore, we show that biofilm assembly by Bap orthologs also depends on the critical N-terminal domain suggesting that the mechanism of biofilm assemblage is conserved in staphylococci. In view of these results, we propose that Bap plays dual role in the bacterial physiology, acting as a sensor and promoting biofilm formation, a configuration that has not hitherto been described for any component involved in biofilm formation.

## Results

### Bap forms aggregates under acidic solution conditions

**Fig 1:**
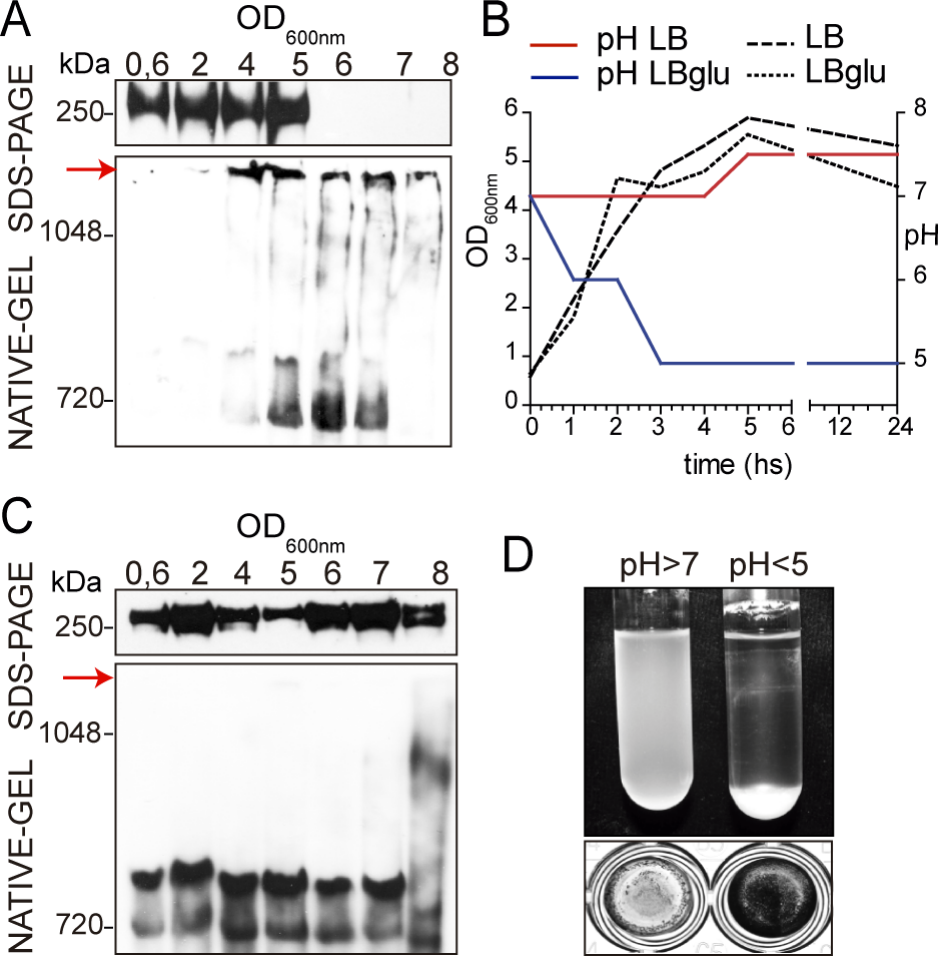
Culture pH determines Bap-mediated aggregation. Cell wall extracts taken at different points of the growth curve from *S*. *aureus* V329 grown in LB-glu (A) or LB (C) were separated on 7.5% acrylamide gels (A and C, upper panel) or Criterion XT Tris-acetate gels with Tris/glycine running buffer (A and C, lower panel) and probed with anti-Bap antibodies. Bap-related insoluble aggregates are indicated by a red arrow. B) Growth curve of *S*. *aureus* V329 culture in LB-glu (dotted line) or LB (dashed line). pH changes in the supernatant of LB-glu culture (blue line) or LB culture (red line) throughout the growth curve. D) Bacterial clumping and biofilm formation of *S*. *aureus* V329 culture in LB (pH>7) and LB-glu (pH<5). For bacterial clumping (upper panel), bacteria were culture overnight with agitation (200 rpm). For biofilm formation, *S*. *aureus* V329 was culture overnight at 37°C in microtiter plates under static conditions. Cell clumps and biofilm formation from 3 independent experiments were quantified (S1A Fig).

To investigate the molecular mechanisms underlying Bap-mediated staphylococcal biofilm development, we monitored the expression of Bap in rich liquid media (LB-glu) along the growth curve using native and denaturing gel electrophoresis. Western immunoblotting under denaturing conditions revealed the presence of Bap from early stages of growth until the population entered stationary phase (OD_600nm_ = 5). From that point, the levels of Bap decreased significantly in denaturing gels, whereas a band of high molecular weight appeared in the native gels suggesting that Bap formed aggregates when bacteria entered stationary growth phase (Fig 1A).

When *S*. *aureus* is grown in a media containing glucose, the entry in stationary phase is accompanied by a decrease in pH due to the accumulation of acidic byproducts from glucose fermentation [39]. We therefore investigated whether Bap aggregation and Bap mediated biofilm development were related and occurred in response to changes in the media pH. To investigate this hypothesis, bacteria were grown in LB-glu, where the pH levels dropped below 5 when bacteria reached stationary phase (OD_600nm_ = 5) and in LB without glucose, where the pH remained neutral all along the growth curve (Fig 1B). Western immunoblotting revealed that Bap failed to form protein aggregates when bacteria grew in LB (Fig 1C). Moreover, results showed a strong correlation between Bap protein aggregates and Bap mediated biofilm formation since bacteria grown in LB-glu (pH<5) formed bacterial clumps and strong biofilms in microtiter plates whereas in LB media (neutral pH) Bap failed to promote bacterial clumping and biofilm development (Fig 1D and S1A Fig). To further corroborate the effect of pH on aggregation of Bap-positive strains we evaluated cell clumping of *S*. *aureus* V329 and ∆*bap* grown in LB medium acidified with 0.1 M HCl to pH 4.5. After an overnight incubation V329 wild type strain clearly showed a biofilm adhered to the microtiter plate and bacterial clumps at the bottom of the tube, while ∆*bap* strain did not (S2A and S2B Fig). Moreover, the two strains were also grown in LB-glu and, after an overnight incubation the medium was replaced by LB to evaluate the possible disassembly of bacterial aggregates. Indeed, no bacterial clumps and no biofilm were observed after media were exchanged indicating that the process of interbacterial interaction mediated by Bap is reversible when pH arises (S2C and S2D Fig). Together, these results suggest that acidification of the growth media promotes Bap aggregation and biofilm development.

### N-terminal region of Bap forms oligomers and mediates biofilm development

**Fig 2:**
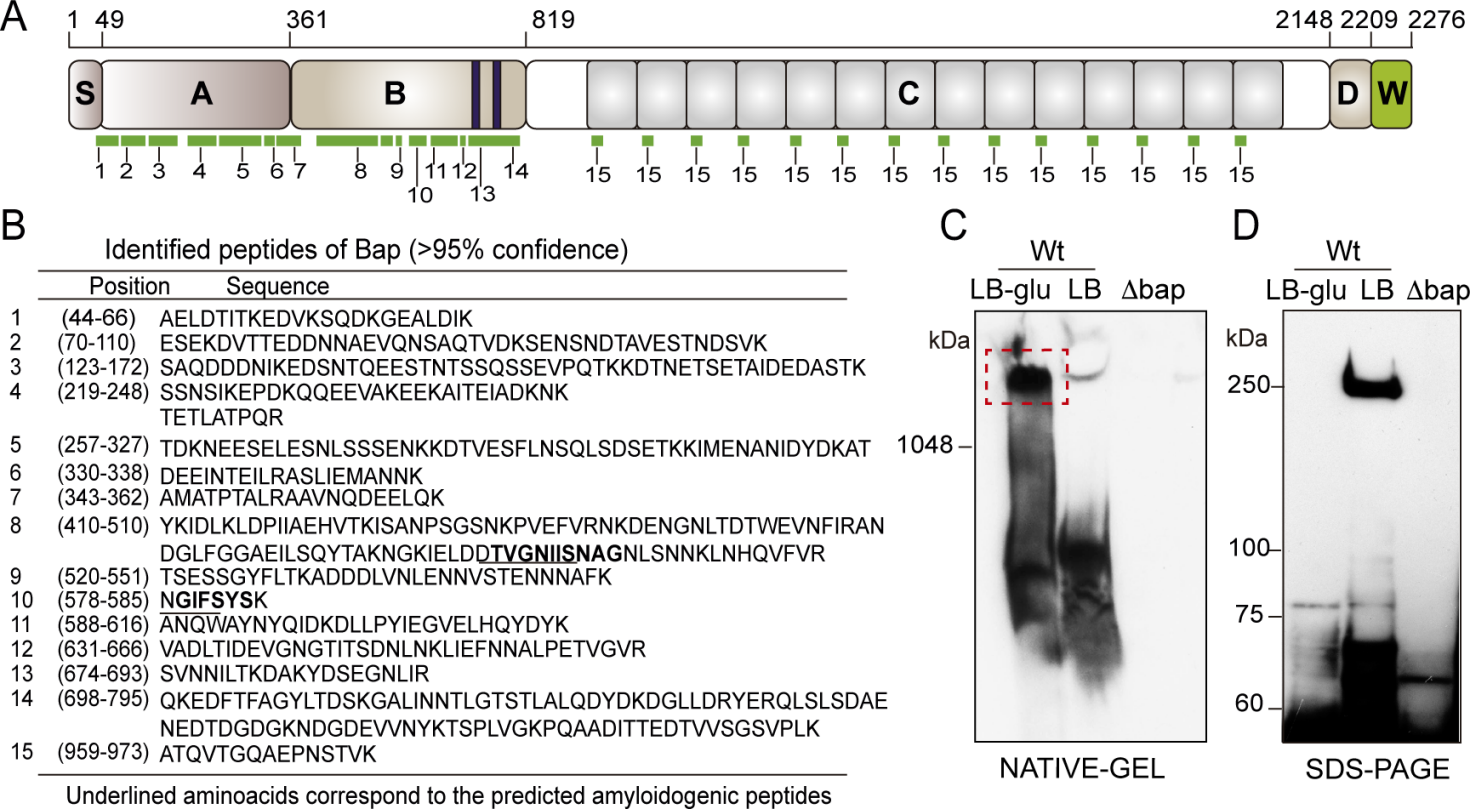
Proteolytic cleavage of Bap protein allows the formation of Bap-aggregates. A) Structural organization of Bap. S, signal peptide; A, region A; B, region B; C, repetition region; D, region of serine-aspartate (SD) repeats; W, cell wall anchor. Green lines correspond to peptides obtained by the MS analysis of the Bap-related insoluble aggregates. B) Summary of the peptides corresponding mostly to the N-terminal region of Bap as identified by MS analysis. Amino acid sequences and positions of identified peptides are shown. C) Insoluble material retained in the native gel pocket that was subjected to MS analysis. Samples were separated in Criterion XT Tris-acetate gels with Tris/glycine running buffer and probed with anti-Bap antibodies. D) Cell wall extracts from *S*. *aureus* V329 stationary culture (OD≈5) grown in LB-glu or LB were separated on 3–8% Criterion Tris-acetate acrylamide gels and probed with anti-Bap antibodies specifically raised against domain B.

If Bap is engaged in homophilic interactions during biofilm development, Bap aggregates should be composed primarily of the Bap protein. In contrast, if Bap mediates heterophilic interactions with other surface proteins, Bap aggregates should also contain additional proteins. To distinguish between these possibilities, we determined the protein content of the Bap aggregates by recovering the insoluble protein material retained within the wells of the stacking gel from preparations of *S*. *aureus* grown in LB-glu and LB and analyzing their identity by mass spectrometry (MS). MS analysis of the material retained in gel pockets from preparations of *S*. *aureus* grown in LB-glu identified peptides that corresponded mostly to the Bap protein strongly suggesting that this polypeptide is the main constituent of the aggregates (S3 Table). Apart from Bap and some ribosomal proteins, the vast majority of the other identified peptides corresponded to proteins that were also detected by MS analysis from preparations of *S*. *aureus* grown in LB medium, where no presence of Bap was observed (S3 Table). We also discarded the possibility that additional matrix molecules such as PNAG or eDNA could be involved in the formation of the Bap aggregates since neither Bap insoluble aggregates nor Bap-mediated biofilms were affected by the treatment with dispersin B (DspB) and DNase I (S3A, S3B and S3C Fig). MS analysis also showed that the identified peptides covered the N-terminal sequence of mature Bap almost completely (amino acid 49 to 819), but only a single short peptide from C repeats region (Fig 2A and 2B) was observed. These results suggest that the insoluble aggregates likely contain Bap fragments that at least include N-terminal region.

To further investigate the mechanism of Bap proteolytic cleavage, we performed western immunoblotting of cell wall extracts of *S*. *aureus* V329 grown in LB-glu and LB culture conditions. Results revealed that Bap was proteolytically processed in both media (Fig 2D), but the cleavage products obtained in LB were unable to form high molecular weight aggregates (Fig 2C). Western immunoblotting of surface proteins from cells grown in LB-glu extracted at different points of the growth curve showed the presence of degradation bands that increased in number and intensity as bacteria grew (S4A Fig). Mass spectrometry analysis of the largest processed band confirmed that it corresponded to a degradation product of Bap containing at least the N-terminal region of the protein (S4B Fig). Interestingly, when bacteria entered stationary phase (OD600nm≈4, pH<5) bands corresponding to full-length and the resulting processed fragments of Bap disappeared from the gel and insoluble aggregates recognized by anti-Bap antibody were readily detectable (S4B Fig).

With the aim to identify the extracellular proteases responsible for the proteolytic processing of Bap, we constructed mutants in 3 extracellular proteases: a serine protease (V8 protease; SspA), a cysteine protease (SspB) and its specific inhibitor (SspC) and a metalloprotease (aureolysin; Aur). The resulting protease-deficient strains showed similar Bap cleavage patterns and formed cell clumps and biofilm at levels similar to wild-type strain (S4 Fig, left panels). Besides, addition to the culture media of protease inhibitors such as α_2_-macroglobulin, E-64 (cysteine protease inhibitor), PMSF (serine protease inhibitor) and the inhibitor Staphostatin A (ScpB) that specifically targets the extracellular cysteine protease ScpA, did not interfere with Bap-mediated aggregation and biofilm development (S4 Fig, right panels). Taking together these findings suggest that Bap is processed either by spontaneous cleavage or by the activity of a protease different from the ones tested here, or perhaps by the action of more than one protease. The resulting processed products are more aggregation prone and form the high molecular weight aggregates under acidic conditions.

### Bap_B domain is sufficient to confer multicellular behavior under acidic culture conditions

**Fig 3:**
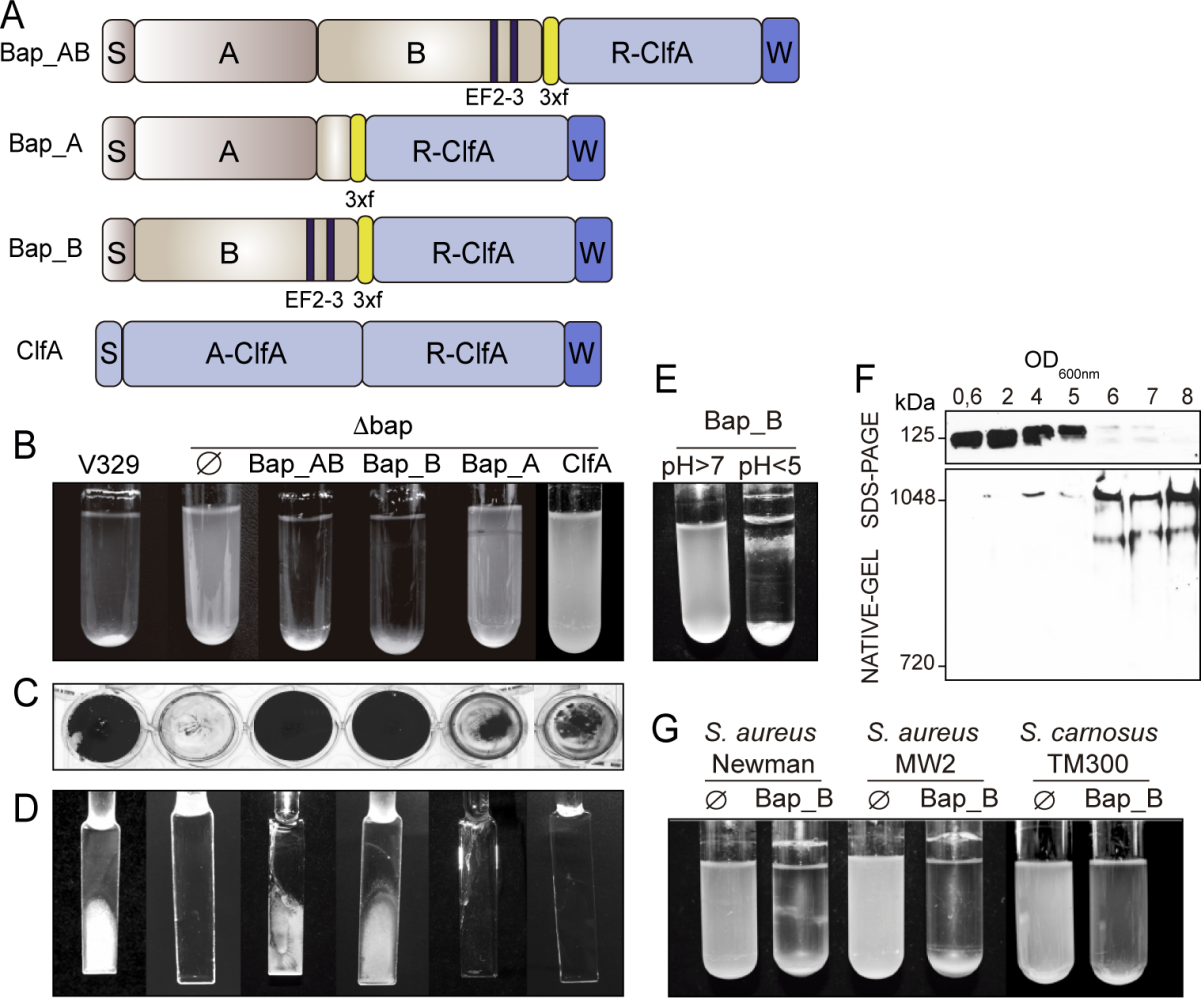
Region B of Bap is sufficient to promote biofilm development. A) Structural organization of Bap chimeric proteins. S, signal peptide; A, region A; B, region B. EF2-3, EF-hand calcium binding motifs 2 and 3; 3xf, 3xFlag tag; R-ClfA, clumping factor R domain; A-ClfA, clumping factor A domain; W, cell wall anchor. B) Bacterial clumping of overnight cultures grown under shaken conditions (200 rpm) at 37°C. C) Biofilm formation in microtiter plates under static conditions. D) Biofilm formed on glass slides under flow culture conditions using microfermentors. E) Bacterial clumping of *S*. *aureus* Δ*bap* mutant expressing Bap_B chimeric protein. Cells were cultured in LB (pH>7) and LB-glu (pH<5). F) Western immunoblotting results demonstrating that Bap_B chimeric protein is sufficient to induce insoluble aggregates. Cell wall extracts from Δ*bap* mutant expressing Bap_B chimeric protein grown in LB-glu were separated on 7.5% acrylamide gels (upper panel) or Criterion XT Tris-acetate gels with Tris/glycine running buffer (lower panel) and probed with anti-Bap antibodies. G) Bacterial clumping of *S*. *aureus* Newman, *S*. *aureus* MW2 and *S*. *carnosus* TM300 complemented with the plasmid carrying the Bap_B chimeric protein or with empty plasmid (Ø). Bacteria were cultured overnight at 37°C with agitation in LB-glu. For assays performed with *S*. *aureus* Δ*bap* expressing the chimeric proteins of Bap, media were all supplemented with 1 μM of CdCl_2_. Cell clumps and biofilm from 3 independent experiments were quantified (S1B Fig).

These latter observations lead us to consider that the N-terminal region of Bap may be sufficient to promote biofilm development. To assess this hypothesis, we generated chimeric proteins comprising different regions of Bap tagged with the 3xFLAG amino acid sequence and linked to the R domain of the clumping factor A (R-ClfA) containing the LPXDG motif (Fig 3A). Variants of Bap comprising domain A (Bap_A, amino acid residues 49 to 361), domain B (Bap_B, amino acid residues 362 to 819), or domain A and B (Bap_AB, amino acid residues 49 to 819) were cloned in pCN51 vector under the control of the P_*cad*_ *-cadC* promoter and expressed in *S*. *aureus* ∆*bap*. The expression of the whole Bap or the chimeric Bap proteins on the bacterial cell wall was verified by western-blot and immunofluorescence using strains deleted in their *spa* gene (V329 Δ*spa* and Δ*bap*Δ*spa*) to avoid unspecific antibody labeling of protein A through its union to the Fc fraction of immunoglobulins (S5 Fig). *S*. *aureus* producing Bap_AB or Bap_B formed huge cell-to-cell aggregates (Fig 3B) and robust biofilms on polystyrene (Fig 3C and S1B Fig) or on a glass surface under flow culture conditions (Fig 3D). In contrast, no cell clusters and biofilm development were found in *S*. *aureus* Bap_A and ClfA strains.

The observation that domain B of Bap is sufficient to induce biofilm phenotype suggests that Bap_B functionality could be affected by the pH. Similarly to the Bap full-length protein, Bap_B formed high molecular weight aggregates when a culture of ∆*bap* strain expressing Bap_B reached stationary phase (Fig 3F). Accordingly, this strain formed biofilm when it was grown in LB-glu (pH<5), but not in LB (Fig 3E and S1C Fig), and showed bacterial clumping and biofilm formation when grown in LB acidified with 0.1 M HCl (S2A and S2B Fig). Bacterial aggregates formed by ∆*bap* expressing Bap_B chimeric protein in LB-glu (pH<5) were disassembled when the medium was exchanged for LB (pH>7) (S2C Fig).

Next, we explored whether Bap_B was sufficient to confer cell-to-cell interactions to naturally *bap* deficient strains: *S*. *aureus* MW2, *S*. *aureus* Newman and *S*. *carnosus* TM300. As shown in Fig 3G and S1D Fig, expression of Bap_B in these strains conferred strong bacterial clumping capacity after an overnight incubation in LB-glu. Taking together, these results indicated that the B domain of Bap (amino acids 362 to 819) is sufficient to bestow multicellular behavior under acidic culture conditions, similarly to the entire Bap protein.

### Purified recombinant Bap_B protein adopts amyloid conformation at acidic pH

**Fig 4:**
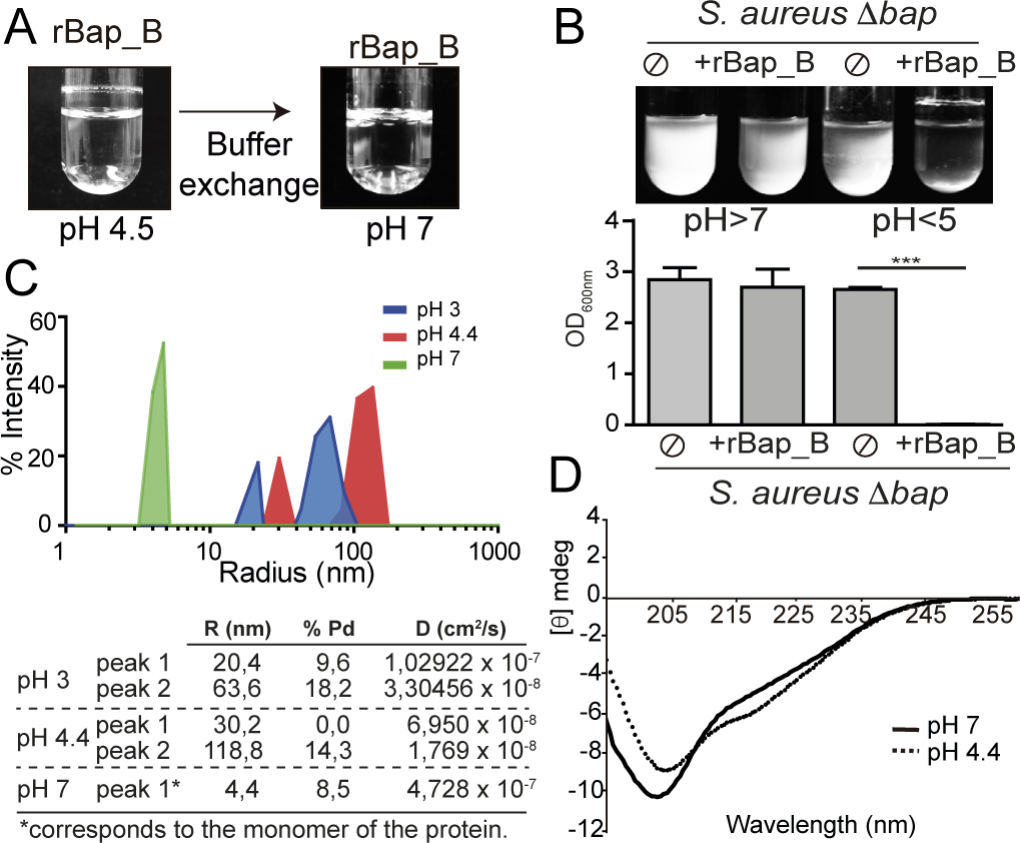
rBap_B aggregation propensity and secondary conformation at different pH. A) Purified rBap_B protein forms visible aggregates when incubated in phosphate-citrate buffer solution at pH 4.5. Reversion assay shows complete disassembly of rBap_B aggregates both at the bottom and on the top of the tube, after performing phosphate-citrate buffer exchange from pH 4.5 to pH 7. B) Bacterial clumping of *S*. *aureus* Δ*bap* grown in LB-glu (pH<5) or LB (pH>7) incubated with 2 μM purified rBap_B protein. Ø, no addition of rBap_B. Cells were incubated for 24 h, with agitation at 37°C. Statistical analysis was carried out using the unpaired Student *t* test (***, *P*<0.001; *n* = 4). C) Hydrodynamic radius (R) of rBap_B in solution at different pH analyzed by dynamic light scattering. Table shows the diffusion coefficient (D) of each population and its corresponding radius (R). (Pd) polydispersity percentage of the corresponding detected population. D) Far-UV CD spectra of 0.2 mg/ml rBap_B at pH 7 (solid line) and pH 4.4 (dotted line).

To get insights about the molecular mechanisms by which the N-terminal region of Bap mediates cell-to-cell interactions, we used a purified recombinant protein comprising exclusively the B region of Bap (rBap_B). Purified rBap_B formed a visible ring of protein adhered to the walls of the tube when incubated at acidic pH in a grade of pH from 3.6 until 5 (S6A Fig). Interestingly, the process was reversible and rBap_B aggregates dissociated completely when the pH was raised to 7 (Fig 4A). To validate the functionality of rBap_B, we analyzed the capacity of rBap_B to restore bacterial clumping phenotype of *S*. *aureus* ∆*bap*. Exogenous addition of rBap_B protein (2 μM) induced bacterial clumping only when *S*. *aureus* ∆*bap* was grown under acidic culture conditions (Fig 4B).

Next, we performed a biophysical characterization of the rBap_B domain. First, we determined the relative size of the aggregates by dynamic light scattering (DLS). The graphic in Fig 4C illustrates the size characterization (hydrodynamic radius R) of rBap_B in solution at different pH. In the table, the correlation between the diffusion coefficient (D) of each population and its corresponding radius is shown. It can be observed that pH 4.4 is the condition at which rBap_B protein presented the highest hydrodynamic radius and the lowest D value (peak 2), as expected for aggregated particles that move slower that smaller particles, with a polydispersity percentage below 15% characteristic of monodispersed samples (Fig 4C). At pH 3, rBap_B presented protein populations with intermediate R values. At neutral pH the obtained peak showed a D that once substituted in the Svedberg equation, together with the previously obtained experimental sedimentation coefficient, buffer density and partial specific volume, corresponded to the monomer of the protein (Fig 4C). Far-UV circular dichroism spectra (CD) of rBap_B showed a moderate increase in β-sheet structure (+5%) when the pH was acidified, at the expense of the predominant non-regular secondary structure (Fig 4D and S4 Table).

**Fig 5:**
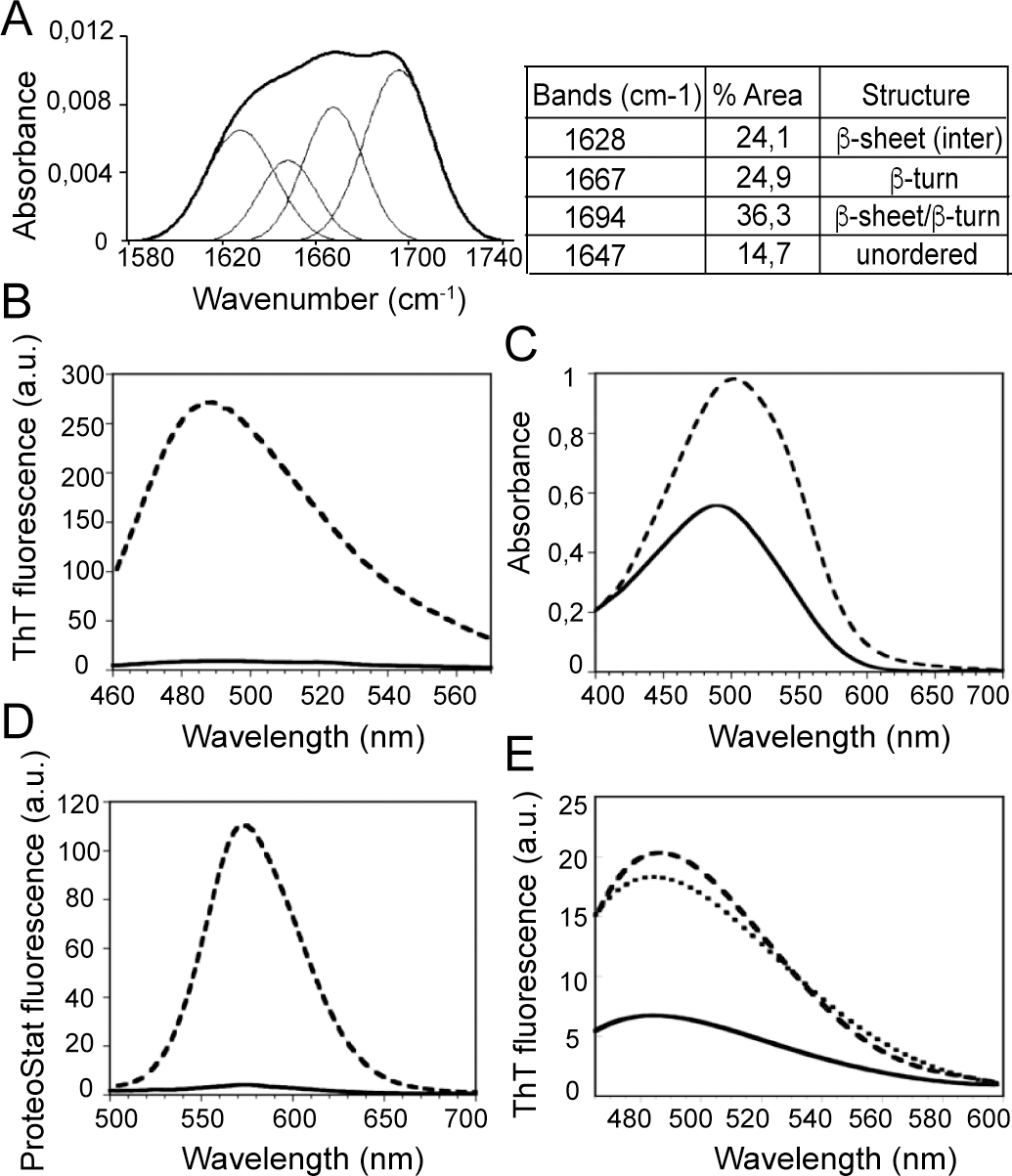
rBap_B aggregates adopt an amyloid-like conformation. A) ATR-FTIR spectrum in the amide I region of rBap_B ring-shaped aggregates formed after 24h in phosphate-citrate buffer pH 4.5 (thick curve). Deconvolution of the ATR-FTIR absorbance spectra into its main secondary structure contributions is shown (thin curves). Percentage of each secondary structure is detailed in the table. B) Increase in ThT fluorescence emission upon binding to 0.1 mg/ml rBap_B ring aggregates at pH 4.5 (dashed line). Free ThT emission spectrum is represented in solid line. C) Shift in CR absorbance spectrum upon binding to 0.1 mg/ml rBap_B from ring aggregates (dashed line). Free CR absorbance spectrum is represented in solid line. D) Increase in ProteoStat fluorescence emission upon binding to 0.1 mg/ml rBap_B ring aggregates (dashed line). Free Proteostat emission spectrum is represented in solid line. E) ThT fluorescence emission spectra upon binding to 10 μM amyloid peptide I (dashed line), and peptide II (dotted line). Free ThT emission spectrum is represented in solid line.

Next, we analyzed more in depth these β-sheet-rich rBap_B aggregates formed at acidic pH. We examined the amide I region of the Attenuated Total Reflectance–Fourier Transform Infrared spectroscopy (ATR-FTIR) spectrum (1700–1600 cm^-1^) of rBap_B ring assemblies. This region corresponds to the absorption of the carbonyl peptide bond group of the protein main chain and is a sensitive marker of the protein secondary structure. Deconvolution of the FTIR-absorbance spectra allowed us to assign the individual secondary structure elements and their relative contribution to the main absorbance signal. The FTIR spectra of rBap_B aggregates was dominated by β-sheet/β-turn components contributing >80% to the signal. In particular, the strong bands at 1628 and 1694 cm^-1^ were consistent with the presence of amyloid-like intermolecular β-sheet structure (Fig 5A). To assess whether the prevalent intermolecular β-sheet in the rings formed by rBap_B aggregates was amyloid-like in nature, we evaluated the binding of the aggregates to the amyloid diagnostic dyes Thioflavin-T (ThT), Congo Red (CR) and ProteoStat. The presence of rBap_B aggregates induced a 25-fold increase in ThT maximum fluorescence emission (Fig 5B). Interestingly, when fresh rBap_B was incubated at pH 4.5 for 5 min it bound readily to ThT in a concentration dependent manner, indicating a fast assembly of rBap_B into ThT positive structures (S7A Fig). In contrast, no change in ThT fluorescence was observed when the protein was incubated at pH 7.0, independent of the protein concentration assayed (S7B Fig). The fast assembly of rBap_B at pH 4.5 was also evident from the strong increase in light scattering relative to the signal obtained at pH 7.0. (S7C Fig). These early assemblies displayed a strong binding to the dye bis-ANS, evidencing the presence of hydrophobic patches exposed to solvent, which potentially might recruit rBap_B monomers into the aggregates and/or contact other cellular molecules through non-polar interactions (S7D Fig). In agreement with an amyloid-like conformation the absorbance of CR and its spectrum maximum red-shifted in the presence of rBap_B ring aggregates (Fig 5C). The absorbance of ProteoStat, a novel fluorescent dye able to stain specifically amyloid aggregates *in vivo* [40], showed a 20-fold increase in its fluorescence maximum at 550 nm (Fig 5D). Altogether, these data strongly suggest that the intermolecular β-sheet structures formed upon aggregation of rBap_B at pH 4.5 posses an amyloid-like conformation.

### Identification and characterization of short amyloid stretches in Bap_B

**Fig 6:**
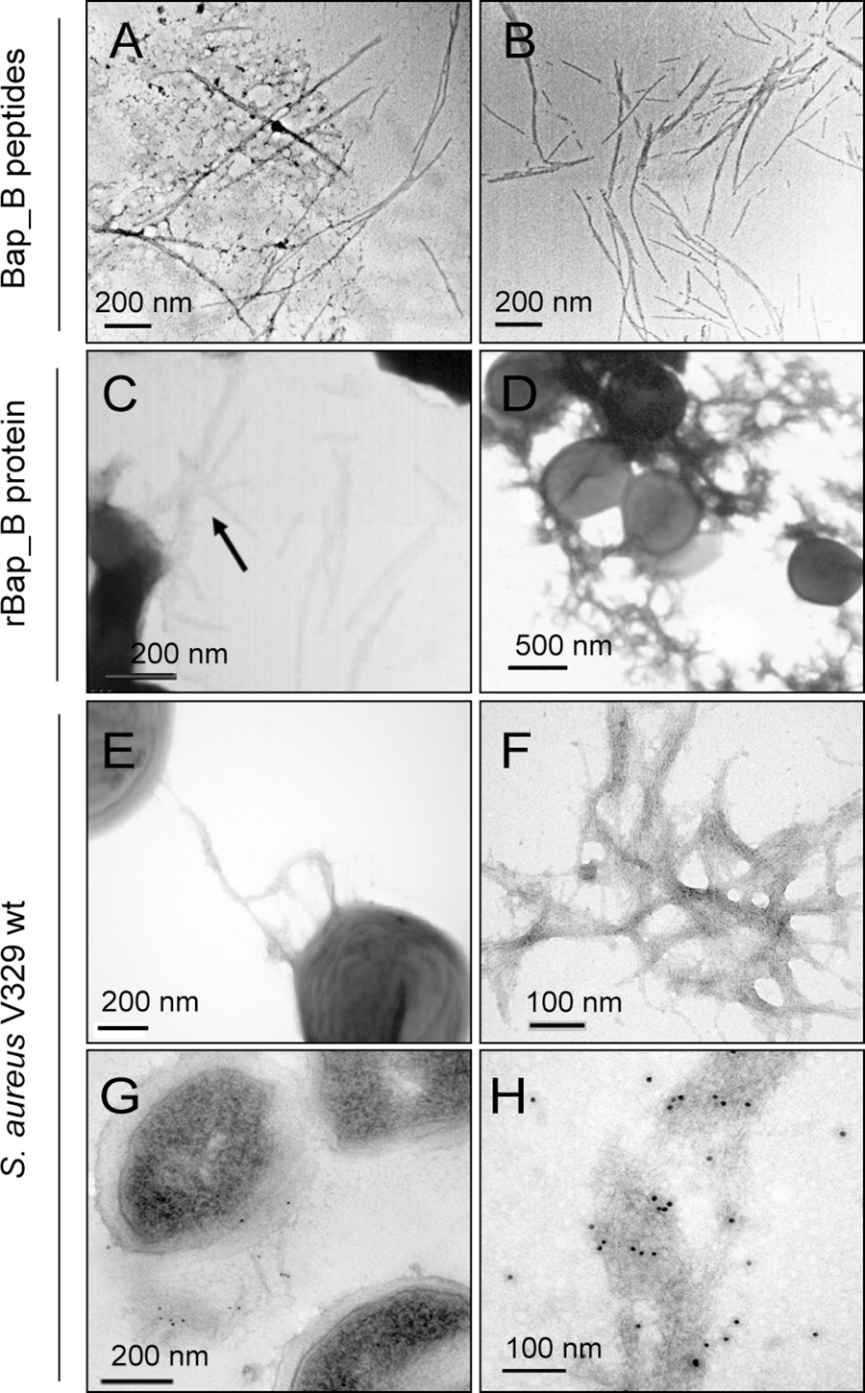
Bap forms amyloid-like fibers. A-B) Electron micrographs of negatively stained fibers formed by amyloid Bap_B peptide I and amyloid Bap_B peptide II respectively. C) Negatively stained fibers formed by purified rBap_B protein. Protein was incubated 24 h in phosphate-citrate buffer pH 4.5. D) Electron micrographs of *S*. *aureus* ∆*bap* cells grown in LB-glu (pH<5) incubated with 2 μM purified rBap_B protein. E) Electron micrographs of negatively stained *S*. *aureus* V329 cells grown in LB-glu (pH<5) for 24 h. G) Immunogold labeled samples from *S*. *aureus* V329 cells grown in LB-glu (pH<5) for 24 h using anti-Bap B antibodies. Fibers are shown at higher magnification in F and H.

In amyloid-like aggregates, short sequence fragments usually promote and guide the formation of amyloid-like structures and become embedded in the inner core of the cross-β structure [41–43]. To identify the likely amyloidogenic regions in the series of Bap_B peptides previously identified by MS in the biofilm we used computational algorithms: AGGRESCAN [44], PASTA [45], WALTZ [46] and ZipperDB [47]. The predictions converged to indicate two Bap_B short sequence stretches as potentially amyloidogenic: TVGNIISNAG named as peptide I (aa 487 to 496), and GIFSYS named as peptide II (aa 579 to 584) (Fig 2B). We synthetized the two peptides and incubated them at 10 μM at pH 4.5. Both peptide solutions formed an evident gel (S8 Fig), a property shared by many amyloidogenic peptides [48] as well as biofilm matrices [49]. Analysis of the structure of the two gels by transmission electron microscopy (TEM) indicated that they comprise fibrils with a typical amyloid morphology (Fig 6A and 6B), and bound to ThT with high affinity (Fig 5E). Taken together these data indicate that Bap_B contains at least two short regions with high amyloidogenic propensity. Analysis of these peptides using the RosettaDesign program [50] implemented in ZipperDB [51] rendered average interaction energies of -25.0 and -25.9 kcal/mol for peptide I and peptide II and shape complementarities between strands of 0.87 and 0.81, respectively. These parameters are compatible with these peptides being able to form steric-zippers that might contribute to N-terminal Bap amyloid assembly. Of course, we cannot discard that the presence of several additional or alternative short amino acid stretches with amyloidogenic tendencies in the sequence of B-domain of Bap would be required for the assembly of the complete domain into aggregated structures at acidic pH.

### Bap protein assembles into amyloid-like fibers at acidic pH

We next determined by transmission electron microscopy the presence of amyloid fibers. Electron microscopy analysis of the aggregates formed by purified rBap_B at pH 4.5, first revealed the presence of isolated fibers and fibers entangled in larger electron dense aggregates (Fig 6C). Similar fibers were detected in the surface of *S*. *aureus* Δ*bap* when bacteria were grown in the presence of exogenously added rBap_B under acidic culture conditions (Fig 6D). We further analyzed the presence of fibers in wild-type *S*. *aureus* V329 grown under biofilm forming conditions. Consistent with all the findings obtained for the rBap_B domain, *S*. *aureus* V329 contained fibers (Fig 6E and 6F) that specifically reacted with gold-labelled anti-Bap antibody (Fig 6G and 6H). Finally, we determined the presence of amyloid fibers in the Bap-mediated biofilm by staining the extracellular matrix of *S*. *aureus* V329 grown in LB-glu medium with ProteoStat, a dye specific of amyloid fibers (S9A Fig) [40]. We also extracted from a gel native pocket the insoluble aggregated material of *S*. *aureus* V329 strain and stained it with ProteoStat. As shown in S9B Fig, the dye stained the protein aggregates formed by *S*. *aureus* V329 but not the Δ*bap* strain.

Also, we tested the effect of the compound (-)-epigallocatechine gallate (EGCG), known to exert an anti-amyloidogenic effect in the case of proteins involved in neurodegenerative diseases [52], on biofilm development by *S*. *aureus* V329 wild type strain. As a control, we used *S*. *aureus* 15981 strain that forms a PNAG-dependent biofilm. ECGC significantly disassembled biofilm formed by *S*. *aureus* V329, but not by 15981, at all concentrations tested (*P*<0.001, *n* = 5) (S9C Fig). These results ratify the amyloidogenic nature of Bap assemblies and their role in biofilm formation.

### Biological relevance of Bap amyloids

**Fig 7:**
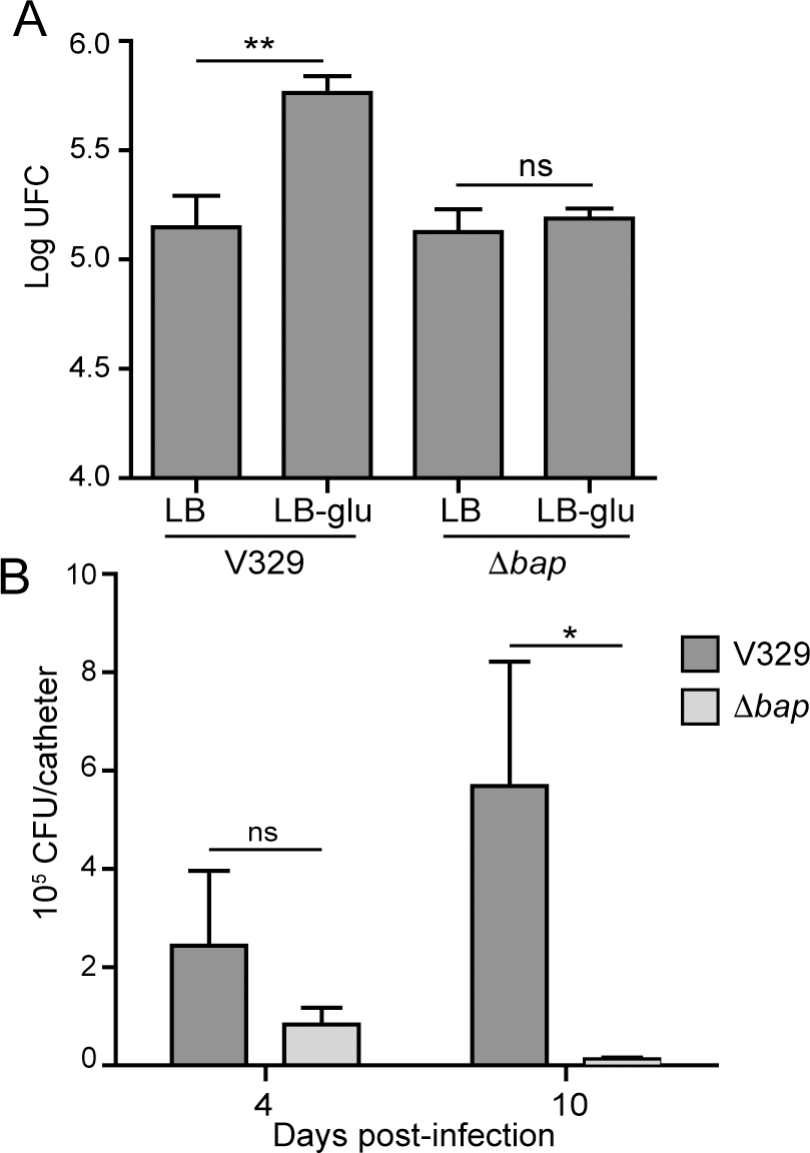
Biological relevance of Bap amyloids. A) Effect of Bap fibers in *S*. *aureus* adhesion to epithelial cells. Adhesion of *S*. *aureus* V329 wild type and Δ*bap* mutant to the mammary gland epithelial cells MAC-T. Bacterial adhesion was determined after 1 hour of infection. Data represent the means from three independent experiments (**, *P*<0.01). B) *In vivo* biofilm formation of *S*. *aureus* V329 wild type and Δ*bap* mutant using a catheter infection model. Catheters were infected with equal numbers of the strains, and bacteria were recovered from implanted catheters and counted after 4 or 10 days post-infection. Asterisks indicate significant differences *P*<0.05. ns, no significant differences. Statistical analysis was performed using the Mann–Whitney test.

In order to investigate the biological relevance of Bap-dependent biofilm formation related to the amyloidogenic properties of domain B, we have analyzed the capacity of *S*. *aureus* V329 strain grown in LB-glu and LB media to adhere to bovine mammary epithelial (MAC-T) cell line. The results revealed that V329 strain adhered more efficiently (*P*<0.01) to epithelial cells when bacteria were grown in LB-glu compared to LB (Fig 7A). Accordingly, the corresponding *S*. *aureus* Δ*bap* mutant strain showed similar capacity to adhere to epithelial cells when grown in LB and LB-glu media. These results suggest that fibers formation would improve *S*. *aureus* adhesion to host cells.

Then, we performed an experiment to evaluate the colonization ability of *S*. *aureus* V329 wild type and Δ*bap* mutant using a mouse foreign body infection model. The reasoning is that *S*. *aureus* V329 wild type should have higher capacity to colonize and persist on catheters than the Δ*bap* strain in the case that Bap mediates biofilm development in this specific environment. To test this hypothesis, sterile catheters were implanted and inoculated into the mice with 10^7^ CFU of *S*. *aureus* V329 and Δ*bap* strains grown overnight in LB-glu at 37°C. Enumeration of *S*. *aureus* cells attached to the catheters 4 days after infection showed slight but not significant differences between *S*. *aureus* V329 wild type and the Δ*bap* mutant strains (Fig 7B). However, at 10 days post-infection, the number of recovered bacteria was significantly higher for the wild type strain (CFU 5.8 x 10^5^) compared to the *bap* mutant (*P*<0.05) (Fig 7B). These results suggest that Bap-mediated biofilm is important for the persistence of *S*. *aureus* through an infection process and, since in *S*. *aureus* V329 biofilm development depends on Bap amyloid fibers, this would imply a key role of these structures in the colonization of indwelling medical devices *in vivo*. However, further studies using different mutant strains that express Bap proteins incapable of aggregate (mutated in the major amyloid sequence stretches required for fibrillation, or mutated in its N-terminal domain) are required.

### Calcium inhibits the formation of Bap amyloid aggregates

Bap-mediated multicellular behavior is inhibited in the presence of millimolar concentrations of calcium bound to the EF-hand domains present in the region B of Bap [36]. The question arises as how calcium and pH environmental signals reconcile to regulate Bap-mediated biofilm formation. To address this question, we investigated the aggregation kinetics of Bap when *S*. *aureus* V329 and ∆*bap* producing Bap_B were grown in LB-glu supplemented with 20 mM of CaCl_2_. The presence of calcium inhibited aggregation of the Bap protein (S10A and S10B Fig, left panels), as well as biofilm formation and bacterial clumping (S10C Fig) despite acidification of the growth media. On the other hand, the wild type V329 and the Δ*bap* Bap_B strains mutated in their EF-hand 2–3 calcium binding motifs (ΔEF and Bap_B_ΔEF respectively) showed no disruption of either biofilm phenotype (S10C Fig) or protein aggregation (S10A and S10B Fig, right panels) in the presence of calcium. Moreover, *S*. *aureus* V329 strain was grown in LB-glu and, after an overnight incubation the medium was replaced by LB-glu containing 20 mM CaCl_2_ to evaluate the possible disassembly of formed biofilm. No disaggregation was observed after the medium was exchanged suggesting that Ca^2+^ inhibitory effect on Bap functionality might be relevant in steps prior to amyloid self-polymerization process (S2D Fig).

**Fig 8:**
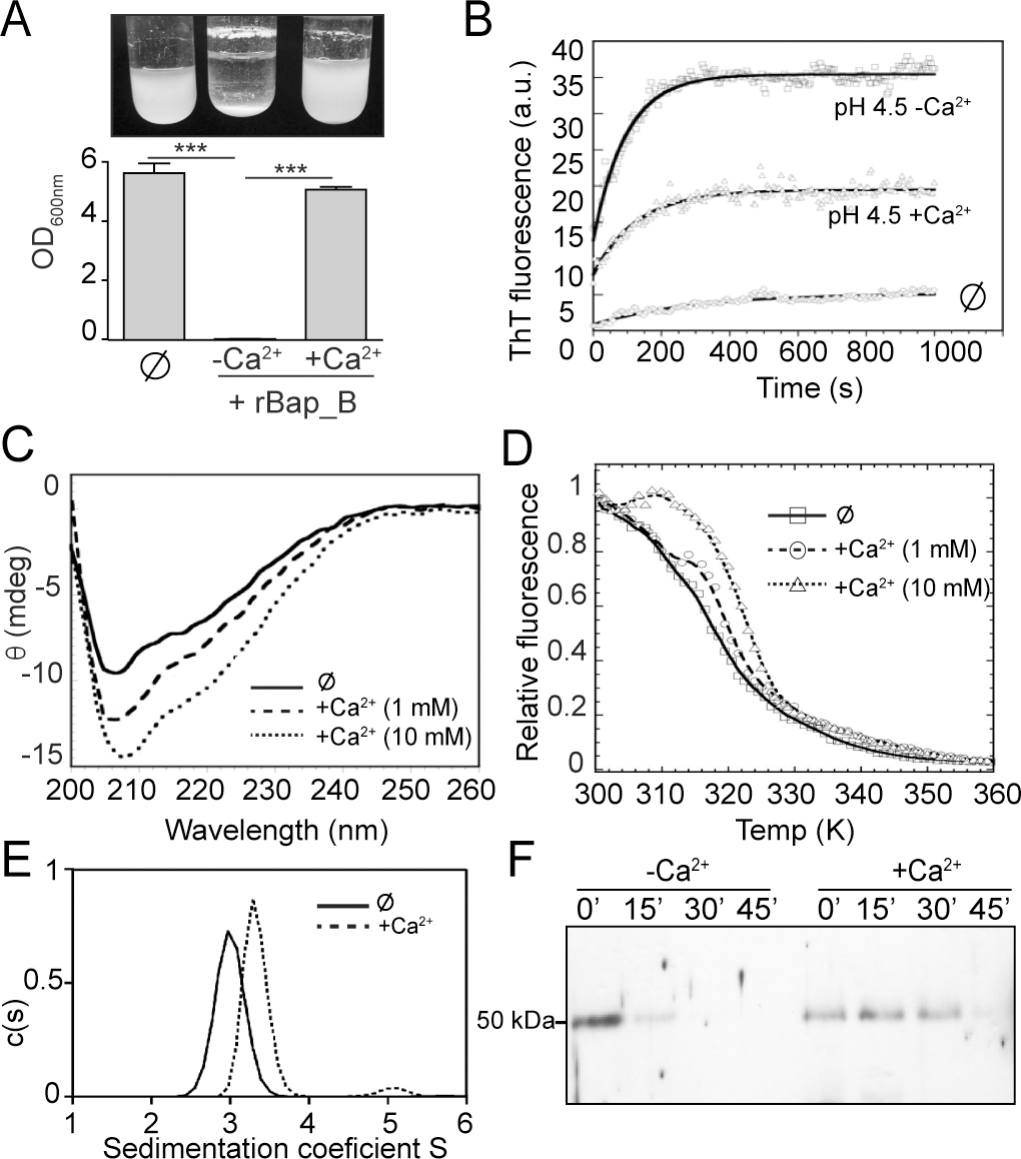
Effect of calcium in rBap_B structure. A) Bacterial clumping of *S*. *aureus* Δ*bap* grown in LB-glu (pH<5) incubated with 2 μM purified rBap_B protein in the presence or absence of 20 mM CaCl_2_. ⊘, no addition of rBap_B. Cells were incubated for 24 h with agitation at 37°C. Statistical analysis was carried out using the unpaired Student *t* test (***, *P*<0.001; *n* = 4). B) Aggregation kinetics of rBap_B were monitored by following the changes in relative ThT fluorescence. rBap_B at pH 4.5 (squares), rBap_B at pH 4.5 + 100mM CaCl_2_ (triangles), and rBap_B at pH 7 (⊘, circles) are represented. C) Far-UV CD spectra of 0.2 mg/ml rBap_B at pH 7 in the absence (solid line) or presence of 1 mM (dashed line) and 10 mM (dotted line) CaCl_2_. D) Thermal melting curves of 0.2 mg/ml rBap_B in the absence (square) or presence of 1 mM (circles) and 10 mM (triangles) CaCl_2_ monitored by intrinsic fluorescence. E) Sedimentation velocity analyses of 1 mg/ml rBap_B in the presence of calcium (dashed line) revealed a specific retard in the sedimentation velocity. The continuous line represents cation-free rBap_B. F) Time course of rBap_B proteinase-K digestion. Immunoblot patterns using anti-BapB antibodies are shown for the protein in the presence (+Ca^2+^) and absence (-Ca^2+^) of 50 mM of CaCl_2_ at 0, 15, 30 and 45 min after proteinase-K addition.

Next, we evaluated the effect of Ca^2+^ on bacterial aggregation of Δ*bap* strain induced by rBap_B under acidic culture conditions. We observed that interbacterial interactions did not occur when Δ*bap* mutant strain exogenously complemented with rBap_B was incubated in the presence of calcium (Fig 8A). We also tested the effect of calcium on Bap amyloid formation by analyzing *in vitro* aggregation of rBap_B into ThT positive amyloid-like structures in the presence of the cation. Results showed that calcium significantly inhibited the formation of amyloid-like aggregates (Fig 8B).

To determine whether the inhibitory effect of Bap-amyloid aggregation induced by calcium is due to a change in protein structure upon binding to the cation we used several biophysical approaches. For this, it is important to clarify that because fast aggregation of rBap_B at pH 4.5 even at low protein concentration (0.01 mg/ml) makes difficult the characterization of the conformational properties of the soluble monomers at this pH, we analyzed the biophysical properties of the Bap_B domain in the presence of calcium at neutral pH. First, 1 and 10 mM CaCl_2_ are sufficient to induce a concentration dependent increase in the ellipticity of the far-UV CD spectra of rBap_B (Fig 8C). Deconvolution of the spectra in the absence and in the presence of 1 mM Ca^2+^ indicated that the protein displays very similar secondary structure content in these conditions. The spectrum is dominated in both cases by disordered conformations, although a small reduction in the overall β-sheet content could be observed in the presence of the Ca^2+^ (the 10 mM CaCl_2_ spectrum could not be deconvoluted due to the strong increase in HT voltage below 200 nm in this condition). We next decided to monitor by near-UV CD and intrinsic fluorescence the overall tertiary structure of rBap_B in the presence or absence of Ca^2+^. Despite no significant impact of Ca^2+^ on the environment of rBap_B aromatic residues could be observed by near-UV CD (S11B Fig), the cation promotes a detectable increase in intrinsic fluorescence emission (S11C Fig), suggesting rearrangements in the tertiary context of the protein. To confirm the existence of a change in the aromatic residues environment, we performed thermal denaturation in the absence and in the presence of 1 and 10 mM CaCl_2_. Results indicated that the temperature at which the protein loses half of its intrinsic fluorescence, augmented in the presence of increasing concentrations of calcium (1 and 10 mM), suggesting that the cation exerts a global stabilizing effect on Bap conformation (Fig 8D). Further techniques support this idea. Analytical ultracentrifugation analysis revealed that, in the presence of calcium the rBap_B monomer exhibited a significantly higher sedimentation coefficient (s(20,w)∼3.4 versus ∼3.0). Additionally, rBap_B protein showed a frictional ratio f/f0 = 1.39, compatible with a slightly elongated protein, while in the absence of the cation the protein showed a frictional ration of 1.66 indicating a more elongated and moderately asymmetric protein shape (Figs 8E and S12A). Size exclusion chromatography analysis supported this by revealing that rBap_B is eluted with retard in the presence of calcium (S12C Fig). Additionally, when we performed 1D-NMR of rBap_B in the presence or absence of Ca^2+^, we observed an increase in the number of peaks corresponding to the methyl (0.5 ppm) and amide (8.5 ppm) regions of the spectrum (S12D Fig). The broader line-widths observed in these regions in the absence of Ca^2+^ are in concordance with a molten globule that is semi stable and fluctuates between several conformations. The sharpening of the peaks when Ca^2+^ is present would then be indicative of protein ordering into a more stable state with a smaller hydrodynamic radius (S12D Fig). Finally, analysis of Bap accessibility to proteolytic degradation in the presence of calcium showed that rBap_B was readily hydrolyzed by proteinase K in the absence of calcium, whereas it was protected from proteinase K activity when the cation was present (Fig 8F). Together all these results are consistent with the idea that Bap protein adopts a transient molten globule-like state in the absence of calcium prior to amyloid formation that is stabilized upon calcium binding thus impeding amyloid assembly due to tertiary rearrangements of Bap conformation.

### N-terminal domain of Bap homologous proteins generates amyloid-like aggregates

**Fig 9:**
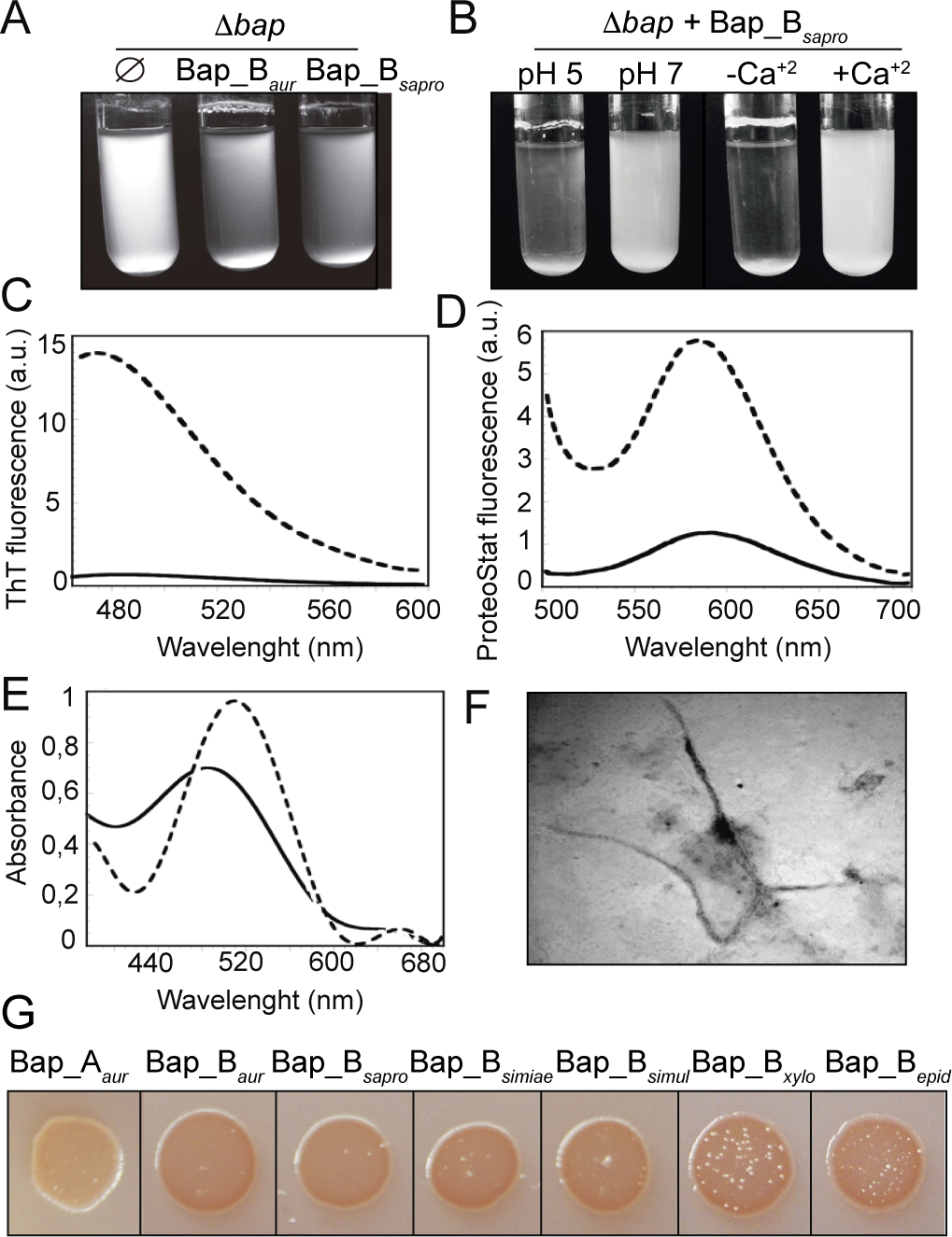
Amyloidogenic behavior of Bap_B orthologous proteins. A) Bacterial clumping of *S*. *aureus* Δ*bap* mutant expressing Bap_B chimeric protein of *S*. *saprophyticus* (Bap_B_sapro_). As a control, Bap_B chimeric protein of *S*. *aureus* was used (Bap_B_aur_). B) Bacterial clumping of *S*. *aureus* Δ*bap* expressing Bap_B_sapro_ cultured in LB (pH 7) and LB-glu (pH 5) and in LB-glu supplemented with 20 mM CaCl_2_. Cell clumps of 3 independent experiments were quantified (S1E Fig). C) Increase in ThT fluorescence emission upon binding to 0.1 mg/ml rBap_B_sapro_ ring aggregates (dashed line). Free ThT emission spectrum is represented (solid line). D) Increase in ProteoStat fluorescence emission upon binding to 0.1 mg/ml rBap_B_sapro_ ring aggregates (dashed line). Free ProteoStat emission spectrum is represented (solid line). E) Shift in CR absorbance spectrum upon binding to 0.1 mg/ml rBap_B_sapro_ aggregates (dashed line). Free CR absorbance spectrum is represented (solid line). F) Electron micrograph of negatively stained aggregates formed by Bap_B_sapro_. G) Variation in colony color phenotype of *E*. *coli* cells exporting the Bap_B orthologous proteins grown on agar supplemented with CR. Bap_B_simiae_: Bap_B from *S*. *simiae*, Bap_B_simul_: Bap_B from *S*. *simulans*, Bap_B_xyl_: Bap_B from *S*. *xylosus*, Bap_B _epid_: Bap_B from *S*. *epidermidis*. As negative control Bap_A from *S*. *aureus* was used (Bap_A_aur_).

Although orthologs of Bap exist in many coagulase-negative staphylococci, homology in region B is variable [35] (S13 Fig and S5 Table). Thus, we wondered whether Bap orthologs could also mediate multicellular behaviour by the generation of amyloid-like aggregates. We selected Bap_B of *S*. *saprophyticus* (Bap_B_sapro_) as it shares an intermediate percentage of identity with Bap_B of *S*. *aureus* (58% identity over the entire length of the B domain). Expression of a chimeric protein containing Bap_B_sapro_ linked to R-ClfA in *S*. *aureus* ∆*bap*∆*spa* (S5 Fig), induced bacterial clumping under acidic culture conditions, but not under basic conditions (Fig 9A and 9B). Consistent with the presence of EF-hand domains, Bap_B_sapro_ was sensitive to the presence of calcium in the media (Fig 9B). As previously shown for rBap_B, purified rBap_B_sapro_ formed precipitated protein aggregates in acidic phosphate-citrate buffer (pH 4.5) that reversibly disassembled after raising the pH to neutral (S6B Fig). Together these results indicate that Bap_B domain of *S*. *saprophyticus* mediates multicellular behavior under acidic culture conditions, analogous to Bap_B domain of *S*. *aureus*.

Biophysical characterization of the rings formed by rBap_B_sapro_ at pH 4.5 indicated that they possess clear amyloid-like features, displaying strong binding to ThT (Fig 9C), ProteoStat (Fig 9D) and CR (Fig 9E). As for rBap_B, light scattering and bis-ANS binding assays demonstrated that rBap_B_sapro_ self-assembled rapidly into aggregates displaying exposed hydrophobic clusters at pH 4.5, whereas it remained soluble at pH 7.0 (S14 Fig). Indeed, when fresh rBap_B_sapro_ was incubated at pH 4.5 for 24 h, the presence of fibrillar structures became apparent (Fig 9F). Finally, we extended the analysis of the amyloid-forming propensity to Bap_B domains of *S*. *simiae*, *S*. *xylosus*, *S*. *epidermidis* and *S*. *simulans* (S5 Table). For that, we used the curli-dependent amyloid generator (C-DAG) system that provides a simple cell-based method to test particular target proteins for their amyloid-forming propensity [53]. The presence of extracellular amyloid aggregates was detected by analyzing the capacity of the strains to bind Congo Red dye (CR). Interestingly, all the Bap_B domain orthologs expressed in C-DAG system were able to bind CR, whereas Bap_A domain of *S*. *aureus* did not (Fig 9G). Together, these data indicate that Bap orthologs also utilize amyloid assembly as a molecular mechanism to induce multicellular behaviour.

## Discussion

There is a growing recognition that proteins play an important role building biofilm matrix scaffold. To fulfill this function these proteins need to provide stable intercellular connections and at least in some cases, also mediate adhesion to the surface. In this report, we have shown that Bap forms extracellular amyloid-like fibers that assist in building the biofilm matrix in *S*. *aureus*. Bap shares structural and functional properties with SasG and Aap proteins of *S*. *aureus* and *S*. *epidermidis* respectively, implicated in cell-to-cell accumulation and adhesion to epithelial cells [38,54–56]. However, the mechanims of action of SasG and Aap are completely different to the one reported here for Bap. All three proteins undergo a limited proteolytic cleavage of the N-terminal domain that induces biofilm formation [15,57]. The mechanism underlying this processing is different among the three proteins. SasG is known to suffer spontaneous cleavage at labile bonds in its B domain, since protease inhibitors added to the growth medium, as well as strains deficient in each known extracellular and membrane-bound proteases, had no effect on the pattern of SasG processing [15]. In the case of Aap, endogenous and also exogenous host-derived proteases are the responsible for protein cleavage, and addition of α_2_-macroglobulin to the growth medium specifically led to the loss of cell clumping and biofilm formation of *S*. *epidermidis* [57]. We failed to identify a staphylococcal protease responsible for Bap cleavage, because protease mutants (∆*aur*, ∆*sspA* and ∆*sspBC*) and protease inhibitors (α_2_-macroglobulin, E64, ScpB and PMSF) did not change the proteolytic pattern of Bap (S4 Fig). However, the possibility that a protease different from the ones tested cannot be discarded and requires further study. In the case of SasG and Aap, the N-terminal domain is removed by proteolysis allowing the C-terminal region containing the G5 domains to promote zinc-dependent self-association of opposing molecules [6,15,57,58]. In contrast, it is the N-terminal region of Bap that is released to the extracellular media and self-assembles into amyloid-like fibers, whilst part of the C-terminal repeats region remains anchored to the membrane. Several pieces of experimental evidence support the amyloid-like properties of the Bap_B domain aggregates. First, far-UV CD spectra reveal a modest switch in secondary structure of Bap from disordered to β-sheet, as the pH becomes more acid. Second, FTIR spectrum of Bap aggregates is dominated by β-sheet/β-turn secondary structure. Third, rBap_B binds to the amyloid diagnostic dyes Thioflavin-T, Congo Red and Proteostat and forms aggregates with fibrillar morphology when observed by electron microscopy. Finally, rBap_B contains short-sequence stretches with significant amyloidogenic potency that together with other unknown sequence stretches could contribute to the fibrillogenesis of Bap fragments. The self-assembly of rBap_B at acidic pH is a fast process, where hydrophobic interactions appear to play an important role, at least at the early stages of the reaction. Genuine bacterial functional amyloids utilize sophisticated machineries that direct the polymerization of amyloid fibers outside the cell. For instance, curli (*csgACB*-*csgDEFG)*, Fap (*fapA-F*) in *Pseudomonas* strain UK4, chaplins (*chpA-H*) in *Streptomyces coelicolor* and TasA (*tapA-sipW-tasA)* in *Bacillus* are expressed together with accessory proteins involved in secretion, nucleation, and assembly of the amyloid subunit [29,30,59–61]. Bap appears to follow a more simplistic model of amyloid auto-aggregation, which does not require the expression of accessory proteins. In this respect, amyloidogenic behavior of Bap could be similar to the mechanism conducted by the surface protein antigen I/II (adhesin P1) of *Streptococcus mutans* [62,63].

**Fig 10:**
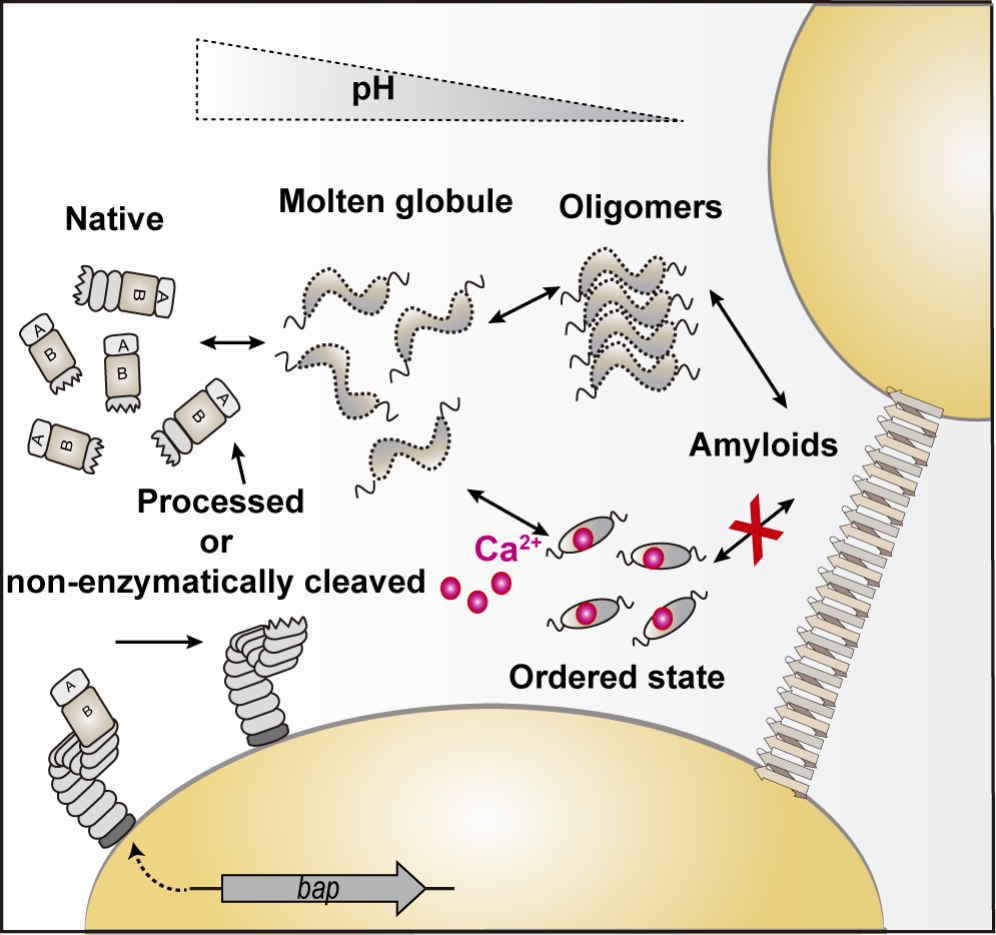
Summary illustrating the effect of pH and calcium in Bap-mediated multicellular behavior. Once located on the bacterial surface, Bap is processed or non-enzymatically cleaved liberating fragments containing the N-terminal domain of the protein that adopt a molten globule-like state. In acidic environments, Bap N-terminal fragments start to self-assemble and ultimately form amyloid fibers that mediate cell-to-cell contacts and biofilm formation through the interaction with the bacterial cell surface by a still unknown mechanism. On the contrary, and in spite of medium acidification, the presence of calcium divalent cations bound to Bap stabilizes the protein. Consequently, Bap is unable to self-assemble into amyloid fibers and does not mediate biofilm formation.

What are the underlying reasons for the conversion of a cell wall anchored protein like Bap into an amyloid fiber? Our results suggest that this strategy allows Bap to play a dual role during biofilm development (Fig 10). Initially, Bap is secreted and covalently anchored to the cell wall. Then, Bap is processed or non-enzymatically cleaved releasing fragments containing the N-terminal region to the media that remain soluble at neutral pH. If the pH of the environment decreases, the N-terminal domain of Bap would transition from its partially ordered native state to an aggregation-prone conformation that would facilitate polymerization into amyloidogenic fibrillar structures. The presence of calcium drastically influences the multicellular behavior promoted by Bap. From a biophysical perspective, the binding of calcium probably to the EF-hand domains of the protein, stabilizes its initial molten globule-like state, likely sequestering the functional N-terminal fragments released from Bap cleavage, and consequently impairing their self-assembly into amyloid structures (Fig 10).

In eukaryotes, there are several examples of proteins involved in aggregation disorders, whose capacity to form multimeric aggregates depend on changes in protein folding caused by binding of metal ions [64]. This is the case of S100A6, an amyloid protein largely expressed in patients with Amyotrophic Lateral Sclerosis (ALS) disease. When S100A6 binds calcium, it suffers a remodeling of the surface electrostatics and hydrophobic patch exposure at the aggregation hotspot inhibiting protein self-assembly into amyloid fibrils [65]. In the case of bacteria, Ca^2+^ bound to α-haemolysin secreted by pathogenic *E*. *coli*, makes the protein more compact, stabilizing its structure and making it less prone to oligomerization [66]. In a similar way, the binding of Ca^2+^ to Bap causes tertiary rearrangements that increase the stability of the intermediate molten globule-like state of Bap in solution and thus decrease its aggregation behavior. This ultimately prevents cellular interactions and biofilm formation in *S*. *aureus*.

Amyloid structures are especially well suited for assembling the biofilm matrix scaffold, as polymerization can occur in the extracellular media in the absence of energy. Furthermore, the amyloid structure provides high stability and inherent resistance against protease digestion and denaturation [28,67]. The pH at which the B domain of Bap shows aggregation activity (pH∼5, early stationary phase) is very close to the isoelectric point (pI∼4.61), where lack of a net charge facilitates interactions between protein molecules, making protein self-assembly more likely. Indeed, a large number of globular and non-globular proteins, including the pathogenic amyloid β peptide and α-synuclein have been shown to display maximum amyloid propensity when they approach their pI, indicating that the solubility of a polypeptide chain is a major factor that determines its conversion to the amyloid state [68]. Because Bap assembly can be reversed when pH is restored to neutrality, it is not difficult to imagine that Bap is able to withstand pH fluctuations, adapting its function by switching from aggregated to soluble forms (and *vice versa*). The ability to fluctuate between soluble and amyloid-like states has been shown to underlie key physiological processes like processing bodies and stress granules formation [69], the cellular response to DNA breakage [70] or the integrity of the cytoskeleton [71]. In *S*. *aureus* this mechanism may have a relevant physiological effect during infectious processes, in which local acidosis usually arises from the accumulation of acidic products as a result of an inflammatory response [72], and bacterial metabolism. Also, this pH-driven Bap-mediated bacterial aggregation mechanism would be physiologically significant for those *Staphylococcus* species expressing Bap homologous proteins that are capable of colonize human host niches displaying mildly acidic conditions (e.g., skin, anterior nares, vagina, urinary tract and mouth) [73,74], as in the case of *S*. *epidermidis* (skin, vagina during prepubertal phase), *S*. *saprophyticus* (urinary tract and vagina) and *S*. *warneri* (skin, nasal cavity, urinary tract).

In a similar way, Foulston *et al* [39] demonstrated that many cytoplasmic proteins reversibly associated with the cell surface in response to pH, acting as a biofilm scaffold matrix in *S*. *aureus*. Regarding calcium ion, its effect on Bap functionality might serve to explain how changes in Ca^2+^ levels during the stages of the lactation cycle affect intramammary infections caused by *S*. *aureus*. Bap displays low binding affinity to calcium, thus medium-to-high millimolar concentrations of the cation are required to saturate Ca^2+^-binding sites in the protein. Normally, the concentration of free Ca^2+^ in mammalian blood is strictly maintained between 1.1–1.3 mM [75,76]}. However, the total Ca^2+^ concentration in milk is higher, around 1.2 mg/liter (~30 mM), being one third of this total amount free in serum (~11 mM) [77]. Thus, Ca^2+^ levels present in the milk during the lactation period are sufficient to inhibit Bap-mediated biofilm development. On the contrary, the low Ca^2+^ concentration conditions that occur in the udder during the dry period allow the formation of Bap-mediated biofilms and the establishment of long-term persistent infections on the mammary gland epithelium [36].

One question that remains open from this study is how Bap amyloid fibers interact with the bacterial surface to induce cell-to-cell aggregation? In *S*. *coelicolor*, it has been proposed that covalently linked cell wall chaplin variants ChpA–C contribute to anchoring the fibers to the cell surface [59]. Following the same reasoning, one would expect that Bap amyloid fibers might interact with the C-terminal domain of Bap that remains covalently anchored to the cell wall. However, the finding that extracellular addition of rBap_B to *bap* deficient *S*. *aureus* strains (Fig 4B) and also to *L*. *monocytogenes* and *E*. *faecalis* (S15 Fig) promoted intercellular adhesion and biofilm formation makes this possibility very unlikely.

Finally, our results indicated that Bap orthologs share similar molecular mechanisms as Bap for mediating biofilm development. *S*. *saprophyticus* is a notable human uropathogen [78]. The pH of the urinary tract varies between 4.5 and 7 [74], representing an environment in which Bap, through the formation of amyloid aggregates and together with urease, UafA [78] and other virulence factors [79] could play an important role in the survival and uropathogenesis of *S*. *saprophyticus*. Except for *S*. *saprophyticus* and *S*. *simiae*, the rest of CNS strains used in this study were mostly isolated from mastitis of lactating dairy cows (cultured from milk samples), a physiological situation in which the presence of calcium, as previously explained, can actually play a relevant role in regulating the functionality of Bap. The diversity of yet unknown factors that could affect Bap amyloid behaviour among different bacterial species is worthy of further study.

## Materials and Methods

### Bacterial strains, culture conditions and plasmids

The bacterial strains and plasmids used in this study are listed in S1 Table. Oligonucleotides were synthesized by StabVida (Caparica—Portugal) (S2 Table). Enzymes for DNA manipulation were supplied by Thermo Scientific and were used according to manufacturer’s recommendations. Staphylococcal strains and *E*. *coli* and were grown in Luria-Bertani (LB) broth or in LB agar (Pronadisa). Media were supplemented when appropriate with 10 μl/ml erythromycin, 100 μg/ml ampicillin, 0.25% wt/vol glucose, 1 μM CdCl_2_, 15 mM CaCl_2_ and 1.25 mM EDTA. Plasmid DNA was isolated from *E*. *coli* strains using a Qiagen plasmid miniprep kit (BioRad), according to the manufacturer’s protocol. Plasmids were transformed into staphylococci by electroporation, using a previously described procedure [21]. Deletion mutants were generated via allelic replacement using the vector pMAD as described previously [80].

### Generation of chimeric proteins

The signal peptide (SP) and the different N-terminal domains of the *bap* gene (AB, B, B∆EF and A) were amplified from *S*. *aureus* V329 and V329 ∆EF [36]. To amplify Bap_AB fragment we used primers Bapori-1mB and Bap-63cK (S2 Table). To obtain Bap_B and Bap_B ∆EF regions we first amplified the signal peptide sequence using primers Bapori-1mB and Bap-65c and second, the region B with primers Bap-66m and Bap-63cK. An overlapping PCR was performed with primers Bapori-1mB and Bap-63cK to get a single fragment. To obtain Bap_A region, two fragments were amplified using primers Bapori-1mB and BapB1 (comprising signal peptide sequence), and BapB2 and BapB3K (comprising A domain). A second overlapping PCR was performed with primers Bapori-1mB and BapB3K in order to obtain a single fragment. To obtain Bap_B region of *S*. *saprophyticus*, we first amplified the signal peptide of Bap from *S*. *aureus* V329 using primers Bapori-1mB and SPbap-sapro-Rv and second, the B-region from *S*. *saprophyticus* B20080011225 using primers bapB-sapro-Fw and Sapbap-KpnI-Rv. An overlapping PCR was performed with primers Bapori-1mB and Sapbap-KpnI-Rv to obtain a single fragment. The entire ClfA fragment used as a control for Bap chimeras, was developed by amplifying *clfA* gene from *S*. *aureus* Newman using primers ClfA-9mB and ClfA-7cE. To allow anchoring of amplified *bap* domains to the bacterial cell wall, the R region of clumping factor A gene containing an LPXTG motif was amplified from *S*. *aureus* Newman strain using primer K-3xF-ClfA containing a flag tag and a recognition sequence for KpnI, and primer ClfA-7cE with a recognition sequence for EcoRI. The KpnI/EcoRI-restricted *R-clfA* was ligated with KpnI/EcoRI-restricted pCN51 vector [81]. The resulting construct was then digested with BamHI and KpnI to insert the previously amplified domains of *bap* gene. The final pCN51 plasmid constructs thus contained different parts of *bap* gene fused to a flag tag followed by the C-terminal R domain of *clumping factor* A gene, expressed under the activity of a cadmium inducible promoter.

### Construction of plasmids for Bap expression in C-DAG system

To obtain *E*. *coli* strains for curli-dependent amyloid generator (C-DAG) system, we PCR amplified from purified genomic DNA (i) region A of *bap* from *S*. *aureus* (primers CDAG BAP_A-Fw and CDAG BAP_A-Rv) and (ii) region B of *bap* from several staphylococcal species: *S*. *aureus* (primers cdag-B-NotI-Fw and cdag-B-XhoI-Rv, S2 Table), *S*. *saprophyticus* (primers BAPsapro-cdag-Fw and BAPsapro-cdag-Rv), *S*. *simiae* (primers BAPsimiae-cdag-Fw and BAPsimiae-cdag-Rv), *S*. *epidermidis* (primers epider-CDAG-Fw and epider-CDAG-Rv), *S*. *simulans* (primers simulans-CDAG-Fw and simulans-CDAG-Rv), and *S*. *xylosus* (primers xylosus-CDAG-Fw and xyosus-CDAG-Rv). The NotI/XhoI-restricted *bapA* and *bapB* fragments were ligated with NotI/XhoI-restricted pEXPORT_XhoI_ plasmid (S1 Table). This vector was obtained by replacing XbaI recognition sequence of the original pEXPORT plasmid [53] for that of XhoI using QuikChange II XL Site-Directed Mutagenesis Kit (Agilent Technologies) and primers pVS72-XhoI-5 and pVS72-XhoI-3. The final pEXPORT constructs were transformed in *E*. *coli* VS39 strain. Induction of protein production and presence of amyloid-like material was assessed on solid medium containing 10 μg/ml Congo Red by evaluating colony-color phenotype, as previously described [53].

### Generation of mutant strains

To generate the deletion in the *aur*, *sspA*, *sspB* genes coding for proteases present in *S*. *aureus*, and a deletion in the *spa* gene coding for surface protein A, we used the pJP437, pJP438, pJP439 [82] and pMAD*spa*AD [20] plasmids which contained two fused fragments of 500 bp each that flanked the left and the right sequence of *aur*, *sspA*, *sspBC* and *spa* genes, respectively. Plasmids were transformed in V329 or Δ*bap* strains by electroporation. Homologous recombination experiments were performed as described [80]. V329 Δ*aur*, Δ*sspA* and Δ*sspBC* (this last strain was also deleted in the cysteine protease inhibitor SspC, which is the last gene of the operon that codifies for SspA and SspB) strains were verified using primers ssp-20cN/ssp-17mS, ssp-24cN/ssp-21mS and aur-Fw/aur-Rv (S2 Table). V329 Δ*spa* and Δ*bap*Δ*spa* strains were verified using primers spaF/spaE (S2 Table).

### Biofilm formation and biofilm dispersion assays

Biofilm formation assay in microtiter wells was performed as described [83]. Briefly, strains were grown overnight at 37°C and then diluted 1:40 in the corresponding media supplemented when required with antibiotics, 20 mM CaCl_2_, or proteases inhibitors (2 U/ml α-macroglobulin, 2 mM cysteine protease inhibitor E64, 10 μM PMSF and 250 nM ScpB). Cell suspension was used to inoculate sterile 96-well polystyrene microtiter plates (Thermo Scientific). After 24 hours of incubation at 37°C wells were gently rinsed two times with water, dried and stained with 0.1% of crystal violet for a few minutes. When desired, crystal violet adhered at the bottom of the wells was resuspended with 200 μl of a solution of ethanol:acetone (80:20 vol/vol) and quantified using a Multiskan EX microplate photometer (Thermo Scientific) with a 595 nm filter.

For biofilm disassembly assays, cells were grown in LB-glu at 37°C on polystyrene microtiter plates. Once formed, adhered biofilm were treated with dispersant agents (0.4 μg/ml Dispersin B, 0.4 μg/ml DNase I, and 20, 100 and 200 μM EGCG) for 2 hours at 37°C. Alternatively, old LB-glu media were extracted and replaced for new LB, LB-glu, and LB-glu + 20 mM CaCl_2_, and incubated overnight at 37°C. Finally, treated and non-treated biofilms adhered to polystyrene wells were macroscopically determined and quantified as previously described.

Biofilm formation under flow conditions was analyzed using microfermenters (Pasteur Institute’s laboratory of Fermentation) with a continuos flow 40 ml/h of LB-glu and constant aeration with sterile pressed air (0.3 bar) [84]. Medium was supplemented with 10 μg/ml erythromycin and 1 μM CdCl_2_ when required. Each microfermentator was inoculated with 10^8^ bacteria from an overnight culture of the corresponding strain. Biofilm development was recorded with a Fugifilm FinePix S5800 digital camera.

### Bacterial clumping assays

Aggregation phenotype in cell suspension was determined as described before [10]. Cells were grown overnight in the corresponding medium (TSB-glu, LB-glu or LB acidified with 0,1 M HCl) at 37°C, shaking at 200 rpm and were examined macroscopically for the presence or absence of aggregates (intercellular adhesion). For bacterial clumping reversion assay, bacteria were grown overnight in LB-glu at 37°C, 200 rpm. Cultures were subsequently centrifuged and LB-glu medium was replaced for LB. After after 6 and 18 h incubation at 37°C, 200 rpm, bacterial aggregation at the bottom of the tube was evaluated for each strain and pictures were taken with a FUJIFILM FinePix S5800 digital camera. To quantify bacterial aggregation, the OD_600nm_ at the top of the culture tubes (approximately 1 cm from the surface) was measured as an estimation of non-settled bacteria (planktonic cells) present in the culture after an overnight incubation at 37°C, 200 rpm. The experiment was independently repeated three times, and data were analyzed with the Mann-Whitney test.

### Microscopy analysis

For immunofluorescence, cells were grown overnight in the corresponding tested conditions and fixed with 3% paraformaldehyde (SIGMA) for 5 minutes. 200 μl of fixed bacteria were placed on coverslips and incubated for 30 minutes. After several washes with PBS, cells were saturated with PBS-0.5% BSA, and finally stained with anti-Bap or anti-Flag (Sigma) antibodies diluted 1:1000. Alexa Fluor 488-conjugated goat anti-rabbit (Invitrogen) diluted 1:200 was used as a secondary antibody and DAPI diluted 1:200 was used to label nuclei. For ProteoStat staining of amyloid material *in-vivo*, cells were grown overnight in LB and LB-glu, at 37°C, in polystyrene 24-wells plates. Adhered biofilm was resuspended and fixed with 3% paraformaldehyde (SIGMA) for 5 minutes. Bacteria were washed several times with 1X PBS, and then incubated for 30 minutes, at room temperature and in darkness with ProteoStat Mix buffer (1X Assay Buffer, 1 μl ProteoStat^®^, 2 μl Hoechst). Bacteria were washed twice with 1X PBS. All preparations were observed with an Axioskop 2 plus epifluorescence microscope (Zeiss) and images were acquired and analyzed with EZ-C1 software (Nikon). For Transmission electron microscopy (TEM), cells were grown overnight in the corresponding tested conditions, washed twice with phosphate-buffered saline (PBS) and then fixed with paraformaldehyde 2% (SIGMA) for 1 hour at room temperature. Formvar/carbon-coated nickel grids were deposited on a drop of fixed sample during five minutes and rinsed three times with phosphate-buffered saline (PBS). Negative staining was performed using 2% uranyl acetate (Agar Scientific, Stansted, UK). Observations were made with a JEOL 1011 transmission electron microscope. For Bap immunogold labelling, grids coated with the sample were washed and incubated for 45 minutes on a drop of PBS containing 1:10 antibody against BapB. After washing with PBS, grids were incubated 45 minutes with gold-conjugated (10nm) goat-anti-rabbit secondary antibody (Aurion, Wageningen, Netherlands). Grids were stained with uranyl acetate as described above.

### Protein expression and purification

Region B of Bap (amino acids 361–819) was PCR amplified from purified *S*. *aureus* V329 genomic DNA using high fidelity Phusion DNA Polymerase (Thermoscientific) and primers bapB1-LIC-Fw and bapB1-LIC-Rv (S2 Table) designed for use in the LIC cloning system. The resulting 1377 bp fragment was cloned in pET46-Ek/LIC vector (Novagen). B-region of Bap from *S*. *saprophyticus* B20080011225 was PCR amplified from its purified genomic DNA using primers bapB-sapro-LIC-Fw and bapB-sapro-LIC-Rv. The resulting 1311 bp fragment was cloned in pET46-Ek/LIC vector (Novagen). Both fusions resulted in Bap_B constructs containing an N-terminal hexahistidine tag (rBap_B and rBap_B_sapro_). Overnight cultures of *Escherichia coli* BL21 DE3 containing Bap_B expression plasmid were diluted 1:100 and grown to an OD_600nm_ of 0.6. Isopropyl B-D-thiogalactopyranoside (IPTG) was added to a final concentration of 0,1 mM and the cultures were shaken at 20°C overnight. After centrifugation, pellets were resuspended, sonicated and centrifuged. Supernatants were filtered (0,45 μm) and rBap_B protein purified by Ni affinity chromatography using HisGraviTrap gravity-flow columns (GE Healthcare). To achieve the highest purity, size exclusion chromatography was applied with a HiLoad 16/600 Superdex 200 pg column (GE Healthcare). The concentration of the purified protein was determined by the Bicinchoninic Acid (BCA) Protein Assay (Pierce, Thermo Scientific) using BSA as a standard.

### Production of anti-BapB antibodies

Rabbit polyclonal antibodies raised against purified rBap_B protein were supplied by Abyntek Biopharma S.L. (Spain). Antibodies were subsequently immunoabsorbed and purified using NAb Spin Kit (Thermoscientific).

### Exogenous complementation

To test extracellular complementation, bacteria were grown in LB, LB-glu or LB-glu + 20 mM CaCl_2_ media mixed with 2 μM-purified rBap_B shaking at 200 rpm at 37°C. Aggregation phenotype in cell suspension was determined and quantified as described above.

### Formation of rBap_B aggregates and reversion assay

To determine the exact pHs at which rBap_B is capable to form aggregates, 2 μM of the protein was incubated in phosphate-citrate buffer at pH ranging from 2.0 until 8. Protein aggregates were macroscopically determined and pictures were taken with a FUJIFILM FinePix S5800 digital camera. For aggregates reversion assay of 2 μM assembled rBap_B and rBap_B_sapro_ protein, the phosphate-citrate buffer at pH 4.5 was removed and exchanged for phosphate-citrate buffer at pH 7. After an overnight incubation at 37°C and 200 rpm, dissolution of rBap_B and rBap_B_sapro_ aggregates was macroscopically determined and pictures were taken with a FUJIFILM FinePix S5800 digital camera.

### Immunoblot analysis

Overnight cultures of *S*. *aureus* strains were diluted 1:100 and grown in LB-glu or LB supplemented with the corresponding antibiotic, 20 mM CaCl_2_ and 1 μM CdCl_2_ when necessary, at 37° C and 200 rpm. Samples were obtained at different point of the growth curve. For protease inhibition assays, diluted *S*. *aureus* V329 cultures were supplemented with proteases inhibitors (2 U/ml α_2_-macroglobulin, 2 mM cysteine protease inhibitor E64, 10 μM PMSF and 250 nM ScpB) and grown until OD≈0.7. Cells were harvested, washed and finally resuspended in 100 μl of PBS buffer containing 30% raffinose (Sigma), 5 μl of lysostaphin 1 mg/ml (Sigma) and 2 μl of DNase 1mg/ml (Sigma). After 2 hours of incubation at 37° C, cells were centrifuged. The supernatants from surface protein extracts were recovered and analyzed by SDS-PAGE or Native gels. For SDS-PAGE, 1 volume of Laemmli buffer was added to the samples and boiled for 5 minutes. 10 μg of protein was used for SDS-PAGE analysis (7,5% separation gel; 5% stacking gel). For Native gels, surface protein extracts were mix 1:2 with native sample buffer (BioRad). Proteins were separated in Criterion XT Tris-acetate gels and Tris/glycine running buffer (BioRad). For Western blot analysis, protein extracts were blotted onto Hybond-ECL nitrocellulose membranes (Amersham Biosciences). Anti-Bap purified antibody and monoclonal anti-Flag M2-Peroxidase (HRP) antibody (Sigma) were diluted 1:20.000 and 1:1000, respectively, with PBS-Tween 5% skim-milk. Alkaline phosphatase-conjugated goat anti-rabbit Immunoglobulin G (Thermo Scientific) diluted 1:5000 in PBS-Tween 5% skim-milk was used as a secondary antibody for Bap detection and the subsequent chemiluminescence reaction was recorded (Chemiluminescent Substrate Thermo Scientific).

### Identification of aggregative peptides

*S*. *aureus* V329 strain was grown in LB and LB-glu media, at 37°C, 200 rpm. After an overnight incubation, cells were harvested, washed and finally resuspended in 100 μl of PBS buffer containing 30% raffinose (Sigma), 5 μl of lysostaphin 1 mg/ml (Sigma) and 2 μl of DNase 1mg/ml (Sigma). After 2 hours of incubation at 37° C, cells were centrifuged. Supernatants were mix 1:2 with native sample buffer (BioRad). Proteins were separated in Criterion XT Tris-acetate gels using Tris/glycine running buffer (BioRad). The material retained in the wells of the native gels was excised, washed three times in ddH_2_0, and digested in-gel with 250 ng of trypsin (Sequencing grade modified Trypsin-Promega) in 50 mM ammonium bicarbonate for 16 h at 37°C, after a denaturation step with DTT (10 mM, 30 min, 40°C) and an alkylation step with Iodoacetamide (25mM, 30 min, room temperature). The resulting peptides were extracted with 1% formic acid, 50% acetonitrile and evaporated to dryness prior to LC-MSMS analysis. For each digested sample, a total volume of 5 μl of tryptic peptides was injected with a flow rate of 300 nL/min in a nanoLC Ultra1D plus (Eksigent). A trap column Acclaim PepMap100 (100 μm x 2 cm; C18, 2 μm, 100 Å) and an analytical column Acclaim PepMap RSLC (75 μm x 15 cm, C18, 5 μm, 100 Å) from Thermo Scientific were used following the next gradient: 0–1 min (5% B), 1–50 min (5–40% B), 50–51 min (40–98% B), 51–55 min (98% B), 55–56 min (5% B), 56–75 min (5% B). (Buffer B: 100% acetonitrile, 0.1% formic acid, Buffer A: 0.1% formic acid). MS analysis was performed on a Q-TRAP 5500 system (ABSciex) with a NanoSpray® III ion source (ABSciex) using Rolling Collision Energy in positive mode. MS/MS data acquisition was performed using Analyst 1.5.2 (AB Sciex) and submitted to Protein Pilot software (ABSciex) against UniprotKB/Swiss-Prot database (restricted to “Staphylococcus”) and then against a specifically restricted database for BAP protein from *Staphylococcus aureus*, using the Paragon™ Algorithm and the pre-established search parameters for 5500 QTRAP.

### Experimental infection

Adherence experiments were performed as described previously [85]. Briefly, prior to use, wells were seeded with 0.3 x 10^6^ MAC-T cells in 6-well tissue culture plates. Once cells were confluent (1.2 x 10^6^) the culture medium was removed and cells were washed with DMEM plus 10% heat-inactivated fetal bovine serum. Overnight bacterial cultures were mixed vigorously and added to the monolayer cells in a multiplicity of infection of 10 in DMEM. Incubation was carried out 1 hour at 37°C in 5% CO_2_. To remove non-adherent bacteria, cells were washed three times with sterile PBS. Eukaryotic cells were lysed with 0.1% Triton X-100. Before plating extracts were mixed vigorously by vortexing. The number of adherent bacteria were determined by serial dilution and plating. Experiments were performed in triplicate.

A mouse foreign body infection model was used to determine the role of Bap aggregates in the pathogenesis of *S*. *aureus*. Groups of 6 CD1 mice were used. A 3-cm segment of intravenous catheter (24G1”, B. Braun) was aseptically implanted into the subcutaneous interscapular space. Each group of six mice was inoculated with 1 x 10^7^ CFU of either *S*. *aureus* V329 or Δ*bap* mutant previosly grown overnight in LB-glu at 37°C. Twelve animals were euthanatized by cervical dislocation on days 4 or 10 post-infection. The catheter was aseptically removed, placed in a sterile microcentrifuge tube containing 1 ml of PBS, and vortexed at high speed for 3 min. Samples were serially diluted and plated onto TSA plates for enumeration of viable staphylococci.

### Ethics statement

All animal studies were reviewed and approved by the Comité de Ética, Experimentación Animal y Bioseguridad, of the Universidad Pública de Navarra (approved protocol PI-019/12). Work was carried out at the Instituto de Agrobiotecnología building under the principles and guidelines described in European Directive 86/609/EEC for the protection of animals used for experimental purposes.

### Proteinase-K digestion

Proteolysis of rBap_B (1 mg/ml) was performed at 37°C in the presence or absence of 50 mM CaCl_2_. The protein was incubated with 80 μg/ml Proteinase-K (SIGMA) for 0, 15, 30, 45 minutes and the reaction was stopped by the addition of 5 mM PMSF. Degradation pattern was analyzed by SDS-PAGE (12%) followed by western immunoblotting with anti-Bap purified primary antibody (1:10.000) and alkaline phosphatase-conjugated goat anti-rabbit Immunoglobulin G (1:5000) (Thermo Scientific) as a secondary antibody.

### Thioflavin T assay

Thioflavin-T (ThT) binding was analyzed for 0.1 mg/ml aggregated rBap_B and rBap_B_sapro_ in the presence of 25 μM ThT, 25°C, pH 4.5. ThT binding was also measured for rBap_B at different concentrations (0.01, 0.018, 0.027 and 0.036 mg/ml) in the presence of 25 μM ThT, 25°C, at pH 4.5 and pH 7. Fluorescence emission spectra were recorded from 460 to 600 nm with an excitation wavelength of 440 nm, using a slit width of 5 nm for excitation and emission in a Jasco FP-8200 spectrophotometer (Jasco corporation, Japan) at 25°C. Each trace represents the average of 5 accumulated spectra. Aggregation kinetics of 0.01 mg/ml rBap_B protein in phosphate-citrate buffer at pH 4.5, pH 4.5 plus CaCl_2_ and pH 7.0 were recorded for 1000 s under agitation (800 rpm) at 25°C, in the presence of 25 μM ThT. The kinetic traces were measured exciting at 440 nm and emission was recorded at 475 nm, slit widths of 5 nm were used for excitation and emission in a Jasco FP8200 spectrophotometer (Jasco corporation, Japan). ThT fluorescence spectra were recorded at the end of the experiment.

### Congo red binding

Congo red (CR) interaction with 0.1 mg/ml aggregated rBap_B and rBap_B_sapro_ at pH 4.5 was tested using a Cary-400 UV/Vis spectrophotometer at 25°C. After 5 minutes of equilibration, the absorbance spectra were recorded from 400 to 700 nm. Each trace represents the average of 5 accumulated spectra.

### ProteoStat binding

Fluorescence emission of 0.1 mg/ml assembled rBap_B and rBap_B_sapro_ stained with ProteoStat was measured on a Jasco FP-8200 fluorescence spectrophotometer (Jasco corporation, Japan) at 25°C. The emission spectra were recorded between 500 and 650 nm wavelength and the samples excited at 484 nm. Slit widths for excitation and emission spectra were 5 nm. The spectra were obtained from the average of 5 consecutive scans.

### ATR-FTIR spectroscopy

ATR FTIR spectroscopy analyses of rBap_B aggregates formed in phosphate-citrate buffer pH 4.5 were performed with a Bruker Tensor 27 FTIR Spectrometer (Bruker Optics Inc.) with a Golden Gate MKII ATR accessory. Spectrum acquisitions consisted of 16 independent scans, measured at a resolution of 2 cm^-1^ within the 1800–1500 cm^-1^ range. Spectra were acquired, background subtracted, baseline corrected and normalized using the OPUS MIR Tensor 27 software. Second derivatives of the spectra were used to determine the frequencies at which the different spectral components were located. All FTIR spectra were fitted to overlapping Gaussian curves using PeakFit package software (Systat Software) and the maximum and the area of each Gaussian were calculated.

### Bis-ANS binding assay

Samples of 0.1 mg/ml rBap_B and rBap_B_sapro_ soluble proteins (phosphate-citrate buffer at pH 7) or protein aggregates (phosphate-citrate buffere at pH 4.5) were prepared in solutions containing 10 μM of Bis-ANS and analyzed immediately on a Jasco FP-8200 fluorescence spectrophotometer (Jasco corporation, Japan) at 25°C. The samples were excited at 370 nm and emission measured between 400 and 600 nm with slit widths of 5 nm. The spectra were obtained from the average of 5 consecutive scans.

### Near-UV and far-UV circular dichroism

Far-UV CD spectra were measured in a Jasco-710 (Jasco, Japan) or in a Chirascan (Applied Photophysics) spectropolarimeter thermostated at 25°C. rBap_B at concentrations ranging from 0.2 to 1.5 mg/ml was measured in 10 mM MOPS either with 1mM CaCl_2_, 10 mM CaCl_2_ or 100 mM CaCl_2,_ or alternatively 10 mM NaCl or 100 mM NaCl and 10 mM EDTA at pH 7.0/7.5. For measurements at acidic pH, rBap_B (6 mg/ml) in 10 mM NaPO_4_ pH 7.0, 50 mM (NH_4_)_2_SO_4_ was diluted 30-fold to 0.2 mg/ml into 100 mM NaPO_4_, 10 mM EDTA at pH 4.4. Spectra were recorded from 260 to 190 nm, at 0.2 nm intervals, 1 nm bandwidth, and a scan speed of 50 nm/min. Twenty accumulations were averaged for each spectrum. Deconvolution of the data were performed using the Dichroweb server implementing the CDSSTR algorithm with reference set 7 [86,87]. Near-UV CD spectra were recorded in a Jasco-710 spectropolarimeter (Jasco, Japan) thermostated at 25°C, from 260 to 320 nm with a 1 nm bandwidth, and a scan speed of 50 nm/min in 10 mM MOPS pH7.0 with 1 mM CaCl_2_, 10 mM CaCl_2,_ or 10 mM NaCl 10 mM EDTA.

### Tryptophan intrinsic fluorescence

Tryptophan intrinsic fluorescence was measured at 25°C on a Varian Cary Eclipse spectrofluorometer using an excitation wavelength of 280 nm and recording the emission from 300 to 400 nm. Five averaged spectra were acquired and slit widths were typically 5 nm for excitation and emission. Protein concentration was 1.5 mg/ml in 10 mM MOPS either with 100mM CaCl_2_ or 100 mM NaCl, 10 mM EDTA at pH 7.5.

### Thermal denaturation

Thermal denaturation was monitored in a Jasco FP-8200 fluorescence spectrophotometer (Jasco, Japan) The samples were excited at 280 nm and the emission was recorded at 350 nm, using slit widths of 5 nm for excitation and emission. The emission was registered each 0.25 K with a heating rate of 0.5 K/min.

### Static light scattering

Static light scattering of 0.1 mg/ml rBap_B and rBap_B_sapro_ in phosphate-citrate buffer at pH 4.5 and pH 7 was recorded on a Jasco FP-8200 spectrofluorometer (Jasco corporation, Japan). Five accumulative spectra were registered with excitation at 330 nm and emission between 320 and 340 nm. Slit widths of 5 nm for excitation and emission were used.

### Dynamic light scattering

Dynamic light scattering data of 1 mg/ml rBap_B protein in phosphate-citrate buffer at pH 3, 4.4 and 7 were obtained with a DynaPro DLS reader (Wyatt Technology, Germany) using an 825 nm wavelength laser and analyzed with Dynamics V6 software. Hydrodynamic radium (nm), polidispersity percentage and diffusion coefficient (cm^2^/s) of each population observed at the different pH values were obtained.

### Gel filtration chromatography

The size exclusion chromatography experiment was performed using a HiLoad 16/600 Superdex 200 pg column (GE Healthcare) connected to an AKTAprime^TM^ Plus chromatography system (GE Healthcare). A 500 μl portion of rBap_B was loaded onto the gel filtration column equilibrated in MOPS buffer (10 mM MOPS, 100 mM NaCl, pH = 7,5) with 100 mM CaCl_2_ or 10 mM EDTA and eluted with one column volume (124 ml) at a flow rate of 1 ml/min. Recorded data were analyzed using PrimeView software (GE Healtcare).

### Analitical Ultracentrifugation (AUC)

All AUC experiments were carried out at 20°C in the presence of 100 mM of CaCl_2_ and 10 mM EDTA, on a Beckman XL-I analytical centrifuge using absorbance optics. Sedimentation velocity was performed for rBap_B at three different concentrations (1, 2 and 3 mg/ml) at 48,000 rpm overnight and the data were analyzed using SedFit 14.7g [88]. Sedimentation equilibrium runs were performed for rBap_B (loading concentrations of 1, 2 and 3 mg/ml) at speeds of 13,000 and 8,500 rpm and analyzed using HeteroAnalysis 1.1.44.

### Nuclear magnetic resonance (NMR)

One-dimensional proton NMR experiments were performed at 30°C on 350 μM Bap_B in a buffer containing 10 mM MOPS, 100 mM CaCl_2_, 10% D2O or 10 mM MOPS, 100 mM NaCl, 10 mM EDTA, 10% D_2_O. Spectra were processed within TopSpin (Bruker).

### Preparation and aggregation of Bap_B short peptides

The predicted peptides GIFSYS and TVGNIISNAG were obtained from CASLO ApS (Lyngby, Denmark) with high purity (99.88% and 98.29% respectively). Peptide stock solutions at 1 mM were prepared by dissolving into citrate buffer. Samples were immediately sonicated for 10 min to dissemble preformed nuclei and centrifuged (5 min at 16,100g) to deposit insoluble material. Peptide solutions were incubated at room temperature (25°C) for four weeks and amyloid properties were evaluated as described above.

### Statistical analysis

The statistical analysis was performed with the GraphPad Prism 5 program. A nonparametric Mann-Whitney test was used to assess significant differences in biofilm formation and bacterial aggregation capacity, as well as for analysis of experimental infection. The differences in bacterial aggregation after exogenous complementation with rBap_B protein were determined using the unpaired Student’s *t* test.

## Supporting Information

S1 Fig Quantification of biofilm formation and aggregation phenotypes of: A) *S*. *aureus* V329 grown in LB (pH>7) and LB-glu (pH<5). B) Δ*bap* strain expressing Bap chimeric proteins cultured in LB-glu. C) Δ*bap* strain expressing Bap_B grown in LB (pH>7) and LB-glu (pH<5). D) *S*. *aureus* Newman and MW2 and *S*. *carnosus* TM300 expressing Bap_B grown in LB-glu. E) Δ*bap* strain expressing Bap_B from *S*. *saprophyticus* cultured in LB (pH>7) and LB-glu (pH<5) and LB-glu + addition of 20 mM of CaCl_2_. Ø: Strains complemented with empty plasmid. Autoaggregation assays demonstrating the settling profiles from liquid suspension were performed by measuring the OD_600nm_ at the top of the culture tubes (1 cm from the surface) as an indication of non-settled cells after an overnight incubation at 37°C, 200 rpm. Static biofilm formation on polystyrene microtiter plates was quantified by solubilizing crystal violet-stained cells with ethanol-acetone (80:20 v/v) and determining the corresponding absorbance at 595nm. Bars represent the mean values from three independent experiments, and error bars represent the standard deviations of the means (*, *P*<0.05; **, *P*<0.01, ns, no statistical differences). Statistical analysis was performed using the Mann–Whitney test.
(TIF)

S2 Fig Effect of pH and calcium addition on Bap aggregation and biofilm formation. Bacterial clumping (A) and biofilm formation (B) of *S*. *aureus* V329, Δ*bap* and Δ*bap* expressing Bap_B cultured in LB media acidified with 0.1 M HCl to a final pH 4.5, at 37°C, 200 rpm. C) Reversibility of bacterial aggregates formed by *S*. *aureus* V329 and Δ*bap* expressing Bap_B chimeric protein cultured in LB-glu (pH<5) with agitation (upper panel). After an overnight incubation at 37°C, the medium was replaced by LB (pH>7), and bacteria were incubated for 6 h (middle panel). LB medium was further replaced by LB-glu (pH<5) to observe bacterial clumping after an overnight incubation (lower panel). Δ*bap* strain was used as a control. D) Biofilm formation of *S*. *aureus* V329 cultured in LB-glu (upper panel). After an overnight incubation the medium was replaced by LB-glu (pH<5), LB (pH>7) and LB-glu + 20 mM CaCl_2_. Bacteria were incubated for 6 h, biofilms were quantified by solubilizing crystal violet-stained cells with ethanol-acetone and the absorbance at 595nm was determined. Data represent the means from three independent experiments.
(TIF)

S3 Fig Role of biofilm matrix molecules in Bap mediated aggregation and biofilm phenotype. For detachment experiments, biofilms formed by *S*. *aureus* V329 and 15981 strains grown in LB-glu for 24 h, were treated with 0,4 μg/ml dispersin B (DspB) (A) or 0,4 μg/ml DNase I (B) for 2 h at 37°C. The quantification of adhered biofilm was performed by the solubilization of crystal violet-stained cells with ethanol-acetone (80:20 v/v) and determination of the absorbance at 595nm. Data represent the means from three independent experiments. ⊘: no treatment. C) Purified Bap aggregates from cell wall extracts were treated with 0,4 μg/ml dispersin B (DspB) or 0,4 μg/ml DNase I for 2 h at 37°C. After treatment, samples were separated in Criterion XT Tris-acetate gels with Tris/glycine running buffer and probed with anti-Bap antibodies. ⊘: untreated V329 cell wall extracts.
(TIF)

S4 Fig A) Western immunoblotting results showing cell surface protein patterns from *S*. *aureus* V329 cells grown in LB-glu. Cell wall proteins extracted at different points of the growth curve were separated on 3–8% Criterion Tris-acetate acrylamide gels and run under denaturing and native conditions and probed with anti-Bap-B antibodies. B) Structural organization of Bap protein. Blue lines correspond to peptides obtained by the MS analysis of the selected band (marked with an asterisk). Amino acid sequences and positions of identified peptides are shown in the bottom table. C) Bacterial clumping of overnight cultures grown in LB-glu of *S*. *aureus* V329 protease-deficient strains: Δ*aur*, Δ*sspA* and Δ*sspBC* and *S*. *aureus* V329 cultured in the presence of protease inhibitors: α_2_-macroglobulin (α_2_-mac), cysteine protease inhibitor (E64), serine protease inhibitor phenylmethylsulfonyl fluoride (PMSF) and Staphostatin A (ScpB) or no addition of protease inhibitor (Ø). D) Biofilm formation in microtiter plates under static conditions. E) Western immunoblotting results showing cell surface protein patterns from protease-deficient strains and *S*. *aureus* V329 grown in the presence of protease inhibitors. Cell wall proteins extracted from exponential cultures (OD≈0.7) were separated on 3–8% Criterion Tris-acetate acrylamide gels and probed with anti-Bap antibodies. The band marked with an asterisk was cut from a Coomasie-stained acrylamide gel and analyzed by mass spectrometry. Δ*bap* strain was used as a control.
(TIF)

S5 Fig Bap-ClfA chimeric proteins are similarly expressed at the bacterial surface of *S*. *aureus*. A) Western-blot analysis showing similar expression levels of Bap chimeras. Cell wall extracts from *S*. *aureus* V329Δ*spa* and Δ*bap*Δ*spa* complemented with the plasmid carrying Bap_AB, Bap_B, Bap_A and ClfA proteins, grown until OD_600nm_ = 4, were separated on 7.5% acrylamide gel and probed with anti-Bap or anti-Flag antibodies. Size markers (in kDa) are indicated. B) Inmunofluorescence showing surface localization of chimeras. Bacteria were fixed and labelled with anti-Bap or anti-Flag antibodies and DAPI.
(TIF)

S6 Fig rBap_B forms reversible aggregates in acidic phosphate-citrate buffer. A) 2 μM of purified rBap_B protein was incubated in phosphate buffer solutions at different pH values. Aggregates were only visible in buffers with pH that fluctuated from 3.6 until 5 (indicated by a red arrow). B) rBap_B_sapro_ forms aggregates at pH 4.5. Reversion assay shows complete disassembly after phosphate-citrate buffer exchange from pH 4.5 to pH 7.
(TIF)

S7 Fig (A) Increase in ThT fluorescence emission upon binding to rBap_B aggregates at pH 4.5 and (B) pH 7. Free ThT emission spectrum is represented in grey. Data for Bap protein at 0.036, 0.027 and 0.018 mg/ml are represented as continuous, dashed and dotted black lines, respectively. (C) Static light scattering of 0.1 mg/ml rBap_B at pH 4.5 (solid line) and pH 7 (dashed line). (D) Bis-ANS fluorescence of 0.1 mg/ml rBap_B at pH 4.5 (solid line) and pH 7 (dashed line). Free bis-ANS is represented in grey.
(TIF)

S8 Fig Gel matrix formed by Bap_B amyloid peptide I and II upon incubation at pH 4.5 for four weeks. (TIF)

S9 Fig Amyloid material is present in the extracellular matrix of *S*. *aureus* V329 biofilms. A) Biofilms from *S*. *aureus* V329 and Δ*bap* strains grown in LB and LB-glu were stained with ProteoStat for 30 minutes. Representative fluorescence microscopic images are shown. B) Insoluble material retained in the native gel pocket from *S*. *aureus* V329 and Δ*bap* strains cultured in LB-glu were extracted from the gel and stained with ProteoStat. C) After an overnight incubation in LB-glu at 37°C, *S*. *aureus* V329 biofilm disassembly in the absence or presence of 20, 100 and 200 μM EGCG was tested. *S*. *aureus* 15981 was used as a control and showed no significant differences of biofilm formation in the presence or absence of ECGC. Bars represent the mean values from five independent experiments (***, *P*<0.001). Statistical analysis was performed using the Mann-Whitney test.
(TIF)

S10 Fig A) Immunoblot of cell wall extracts from *S*. *aureus* V329 and *S*. *aureus* ΔEF-hand and B) Δ*bap* mutant expressing Bap_B chimeric protein and Δ*bap* expressing Bap_B_ΔEF. Bacteria were grown in LB-glu supplemented with 20 mM CaCl_2_. Proteins were separated on 7.5% acrylamide gels (upper panel) or Criterion XT Tris-acetate gels with Tris/glycine running buffer (lower panel) and probed with anti-Bap antibodies. Size markers (in kDa) are indicated. C) Bacterial clumping and biofilm formation of *S*. *aureus* V329 and ΔEF mutant; Δ*bap* expressing Bap_B and Δ*bap* expressing Bap_B_ΔEF grown in LB-glu supplemented (+) or not (-) with 20 mM CaCl_2_.
(TIF)

S11 Fig A) Effect of calcium in secondary structure composition of Bap determined by far-UV CD. Data were analyzed with Dichroweb implementing the CDSSTR algorithm. B) Near-UV CD spectra of 0.2 mg/ml rBap_B in the absence (black) or presence of 1 mM (red) and 10 mM (green) CaCl_2_. C) Intrinsic fluorescence of 1.5 mg/ml rBap_B at pH 7 in the presence (solid line) or absence (dashed line) of 100 mM CaCl_2_.
(TIF)

S12 Fig A) Summary of sedimentation velocity and sedimentation equilibrium parameters of rBap_B at pH 7 and in the presence or absence of calcium confirms monomeric state of the protein in both conditions. B) Sedimentation equilibrium analysis of 2 mg/ml rBap_B in the presence or absence of CaCl_2_, and at different rpm. Dotted lines show global fits. Residuals are shown above. C) Size exclusion chromatography of rBap_B. Chromatograms showing the protein eluted from a HiLoad 16/600 Superdex 200 pg column in buffers in the presence (dashed line) and absence (solid line) of 100 mM CaCl_2_. D) 1D ^1^H nuclear magnetic resonance (NMR) spectrum of 350 μM Bap_B in 10 mM MOPS, 100 mM CaCl_2_ (red) overlaid on that for 350 μM Bap_B in 10 mM MOPS, 10 mM EDTA (blue). The methyl region of the NMR spectrum includes high-field proton resonances observed at low chemical shifts (<0.5 ppm), which indicate the presence of characteristic clusters of aromatic and methyl groups in the core of a structured protein. In addition, the envelope of peaks resonating at high chemical shift (>8.5 ppm) correspond to highly ordered backbone amides present in secondary structure elements. The increase in the number of peaks within the fingerprint methyl and amide regions (arrowed) in the presence of Calcium indicate binding and induced structure in the Bap_B region.
(TIF)

S13 Fig Protein sequence alignment of Bap orthologs. The alignment between regions B of Bap from 16 strains was generated using ClustalW2 multiple sequence alignment tool through Jalview program. GeneBank accession numbers are shown in brackets for each strain.
(TIF)

S14 Fig (A) Bis-ANS fluorescence and (B) static light scattering of 0.1 mg/ml rBap_B_sapro_ at pH 4.5 (solid line) and pH 7 (dashed line). Free bis-ANS is represented in grey.
(TIF)

S15 Fig Exogenous complementation of heterologous gram-positive bacteria with rBap_B purified protein. Bacterial clumping of *L*. *monocytogenes* EGD and *E*. *faecalis* 23 was evaluated after an overnight incubation at 37°C, 200 rpm, in the presence of 2 μM rBap_B protein. Data represent the means from three independent experiments. Error bars represent standard deviation (**, P<0.01; ***, P<0.001). Statistical analysis was performed using the unpaired Student *t* test.
(TIF)

S1 Table Strains and plasmids used in this study. (PDF)

S2 Table Oligonucleotides used in this study. (PDF)

S3 Table Summary of the proteins identified by proteomics from aggregates of *S*. *aureus* in LB-glu and LB. (PDF)

S4 Table Secondary structure composition of Bap under different conditions as determined by far-UV CD. Data were analyzed with Dichroweb implementing the CDSSTR algorithm. *NRMSD = [Σ(θexp– θcal)2 / Σ(θexp)2]1/2 where θexp and θcal are the experimental and calculated ellipticity values at a particular wavelength, respectively.
(PDF)

S5 Table Sequence comparison of B region of Bap between different staphylococcal species. (PDF)

S1 References Supporting Information References. (PDF)
