# Supplementary figures and images for: Staphylococcal Bap Proteins Build Amyloid Scaffold Biofilm Matrices in Response to Environmental Signals
Source: PLoS Pathog. 2016 Jun 21;12(6):e1005711. doi: 10.1371/journal.ppat.1005711 (PMC4915627; doi:10.1371/journal.ppat.1005711)

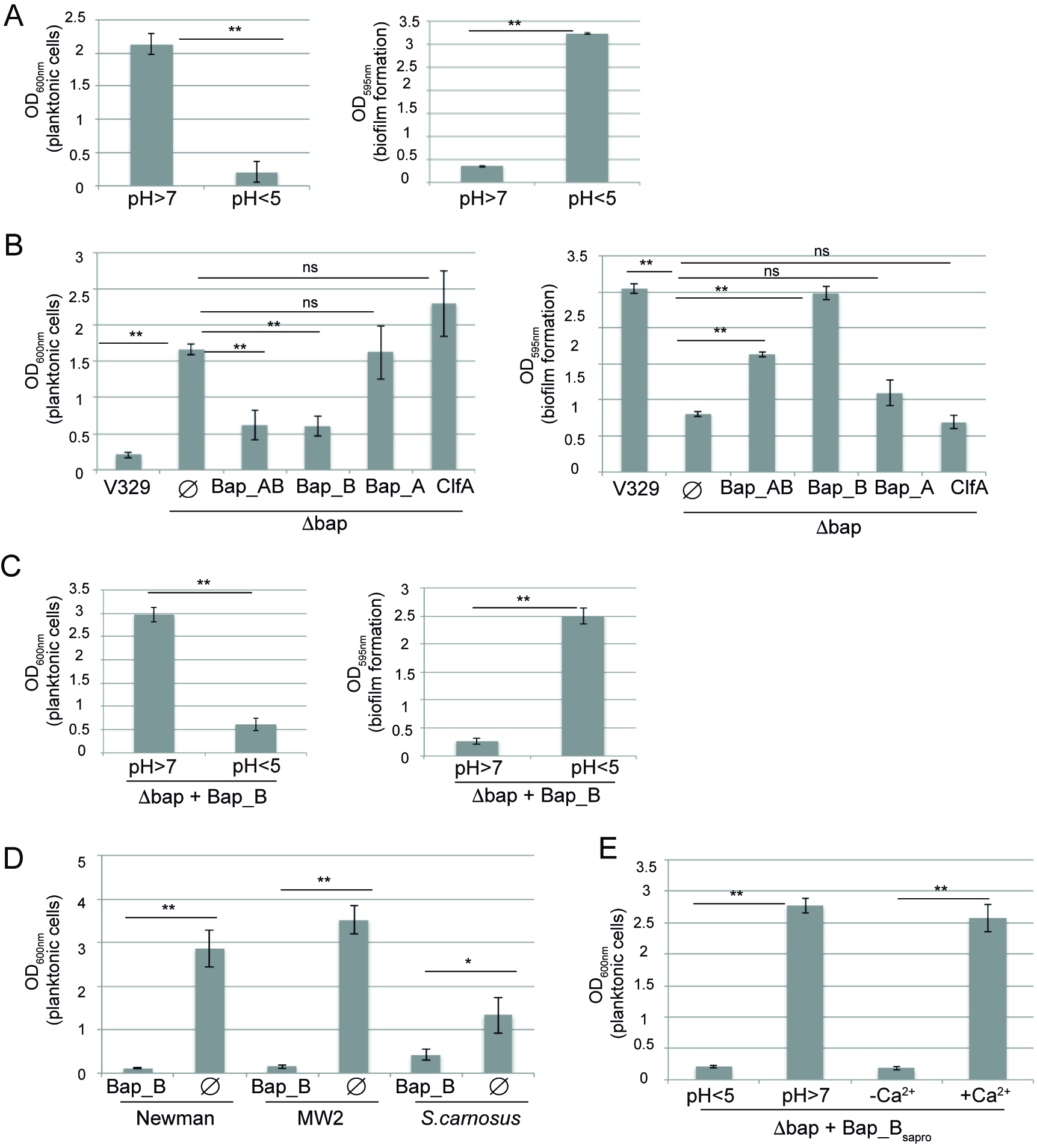

Supplement: S1 Fig — Quantification of biofilm formation and aggregation phenotypes of: A) S. aureus V329 grown in LB (pH>7) and LB-glu (pH<5). B) Δbap strain expressing Bap chimeric proteins cultured in LB-glu. C) Δbap strain expressing Bap_B grown in LB (pH>7) and LB-glu (pH<5). D) S. aureus Newman and MW2 and S. carnosus TM300 expressing Bap_B grown in LB-glu. E) Δbap strain expressing Bap_B from S. saprophyticus cultured in LB (pH>7) and LB-glu (pH<5) and LB-glu + addition of 20 mM of CaCl2. Ø: Strains complemented with empty plasmid. Autoaggregation assays demonstrating the settling profiles from liquid suspension were performed by measuring the OD600nm at the top of the culture tubes (1 cm from the surface) as an indication of non-settled cells after an overnight incubation at 37°C, 200 rpm. Static biofilm formation on polystyrene microtiter plates was quantified by solubilizing crystal violet-stained cells with ethanol-acetone (80:20 v/v) and determining the corresponding absorbance at 595nm. Bars represent the mean values from three independent experiments, and error bars represent the standard deviations of the means (*, P<0.05; **, P<0.01, ns, no statistical differences). Statistical analysis was performed using the Mann–Whitney test. (TIF) [file ppat.1005711.s001.tif]

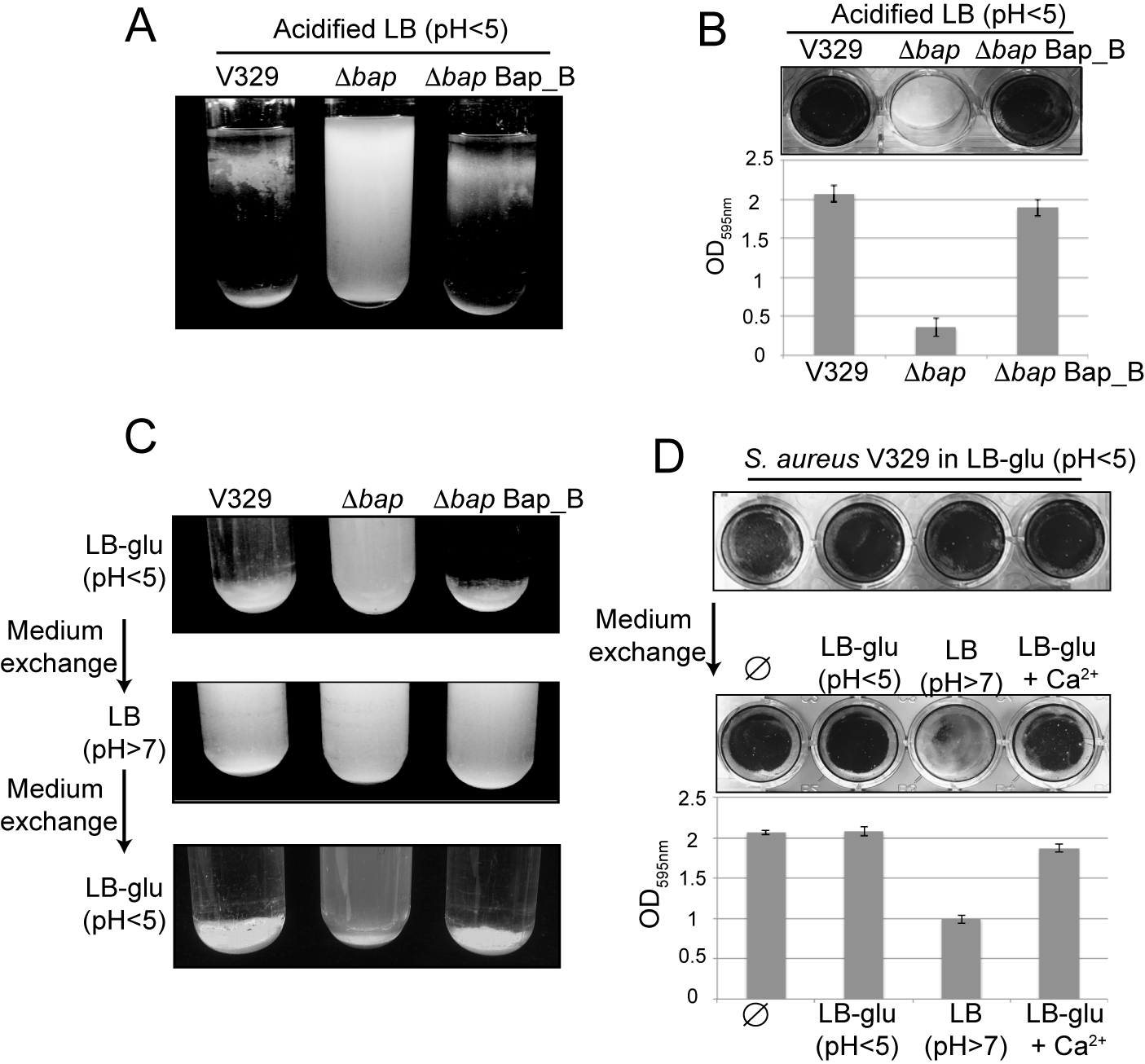

Supplement: S2 Fig — Bacterial clumping (A) and biofilm formation (B) of S. aureus V329, Δbap and Δbap expressing Bap_B cultured in LB media acidified with 0.1 M HCl to a final pH 4.5, at 37°C, 200 rpm. C) Reversibility of bacterial aggregates formed by S. aureus V329 and Δbap expressing Bap_B chimeric protein cultured in LB-glu (pH<5) with agitation (upper panel). After an overnight incubation at 37°C, the medium was replaced by LB (pH>7), and bacteria were incubated for 6 h (middle panel). LB medium was further replaced by LB-glu (pH<5) to observe bacterial clumping after an overnight incubation (lower panel). Δbap strain was used as a control. D) Biofilm formation of S. aureus V329 cultured in LB-glu (upper panel). After an overnight incubation the medium was replaced by LB-glu (pH<5), LB (pH>7) and LB-glu + 20 mM CaCl2. Bacteria were incubated for 6 h, biofilms were quantified by solubilizing crystal violet-stained cells with ethanol-acetone and the absorbance at 595nm was determined. Data represent the means from three independent experiments. (TIF) [file ppat.1005711.s002.tif]

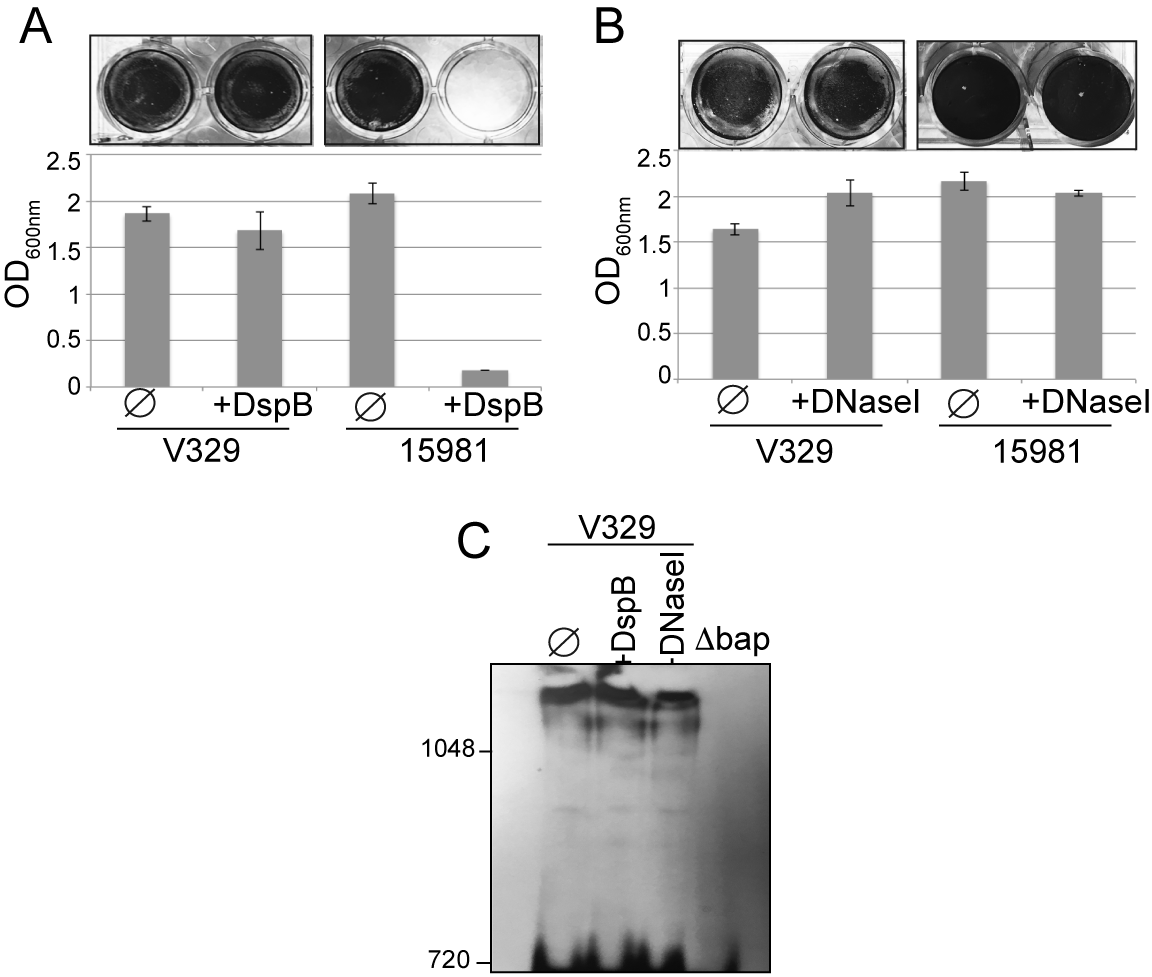

Supplement: S3 Fig — For detachment experiments, biofilms formed by S. aureus V329 and 15981 strains grown in LB-glu for 24 h, were treated with 0,4 μg/ml dispersin B (DspB) (A) or 0,4 μg/ml DNase I (B) for 2 h at 37°C. The quantification of adhered biofilm was performed by the solubilization of crystal violet-stained cells with ethanol-acetone (80:20 v/v) and determination of the absorbance at 595nm. Data represent the means from three independent experiments. ⊘: no treatment. C) Purified Bap aggregates from cell wall extracts were treated with 0,4 μg/ml dispersin B (DspB) or 0,4 μg/ml DNase I for 2 h at 37°C. After treatment, samples were separated in Criterion XT Tris-acetate gels with Tris/glycine running buffer and probed with anti-Bap antibodies. ⊘: untreated V329 cell wall extracts. (TIF) [file ppat.1005711.s003.tif]

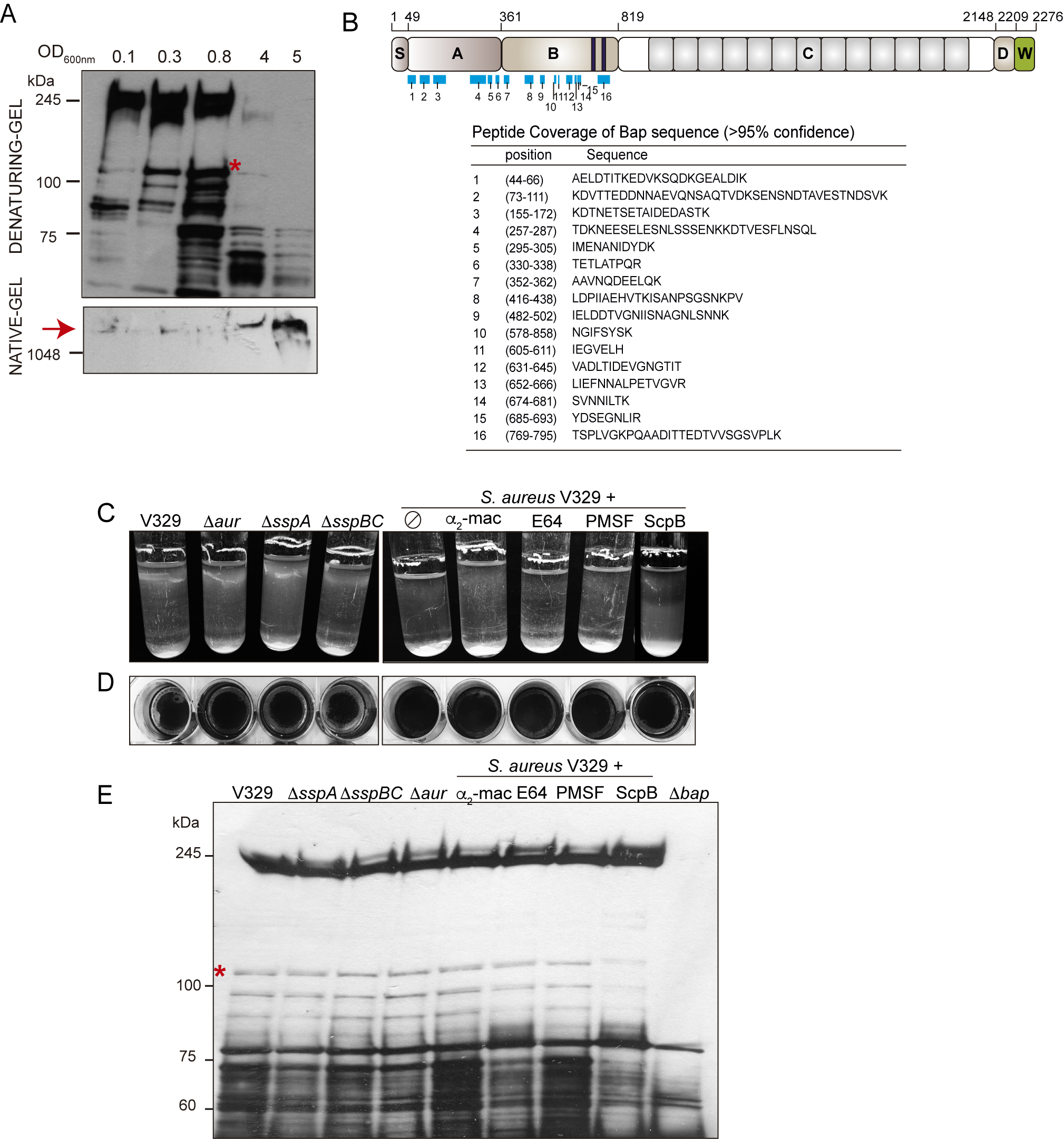

Supplement: S4 Fig — A) Western immunoblotting results showing cell surface protein patterns from S. aureus V329 cells grown in LB-glu. Cell wall proteins extracted at different points of the growth curve were separated on 3–8% Criterion Tris-acetate acrylamide gels and run under denaturing and native conditions and probed with anti-Bap-B antibodies. B) Structural organization of Bap protein. Blue lines correspond to peptides obtained by the MS analysis of the selected band (marked with an asterisk). Amino acid sequences and positions of identified peptides are shown in the bottom table. C) Bacterial clumping of overnight cultures grown in LB-glu of S. aureus V329 protease-deficient strains: Δaur, ΔsspA and ΔsspBC and S. aureus V329 cultured in the presence of protease inhibitors: α2-macroglobulin (α2-mac), cysteine protease inhibitor (E64), serine protease inhibitor phenylmethylsulfonyl fluoride (PMSF) and Staphostatin A (ScpB) or no addition of protease inhibitor (Ø). D) Biofilm formation in microtiter plates under static conditions. E) Western immunoblotting results showing cell surface protein patterns from protease-deficient strains and S. aureus V329 grown in the presence of protease inhibitors. Cell wall proteins extracted from exponential cultures (OD≈0.7) were separated on 3–8% Criterion Tris-acetate acrylamide gels and probed with anti-Bap antibodies. The band marked with an asterisk was cut from a Coomasie-stained acrylamide gel and analyzed by mass spectrometry. Δbap strain was used as a control. (TIF) [file ppat.1005711.s004.tif]

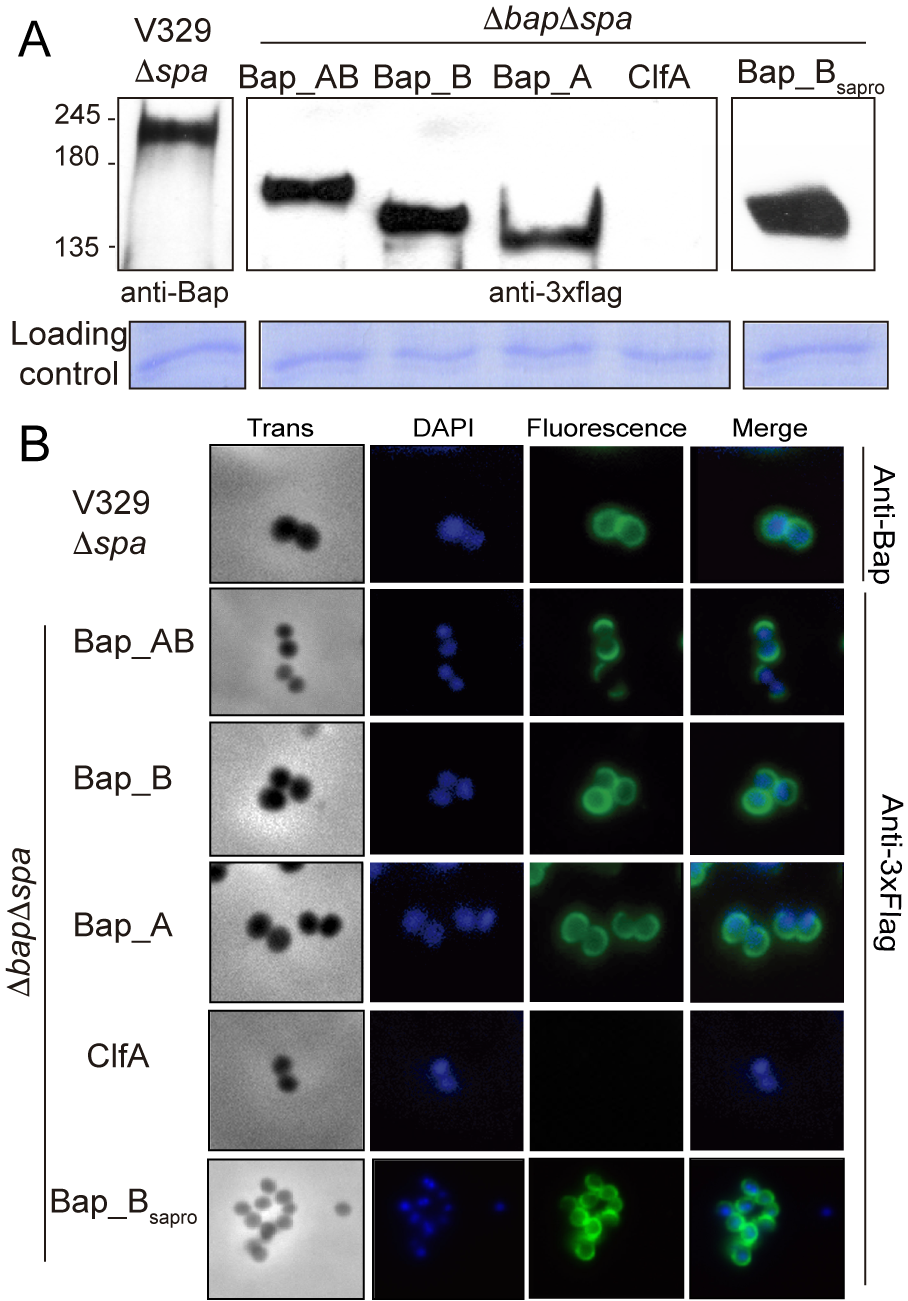

Supplement: S5 Fig — A) Western-blot analysis showing similar expression levels of Bap chimeras. Cell wall extracts from S. aureus V329Δspa and ΔbapΔspa complemented with the plasmid carrying Bap_AB, Bap_B, Bap_A and ClfA proteins, grown until OD600nm = 4, were separated on 7.5% acrylamide gel and probed with anti-Bap or anti-Flag antibodies. Size markers (in kDa) are indicated. B) Inmunofluorescence showing surface localization of chimeras. Bacteria were fixed and labelled with anti-Bap or anti-Flag antibodies and DAPI. (TIF) [file ppat.1005711.s005.tif]

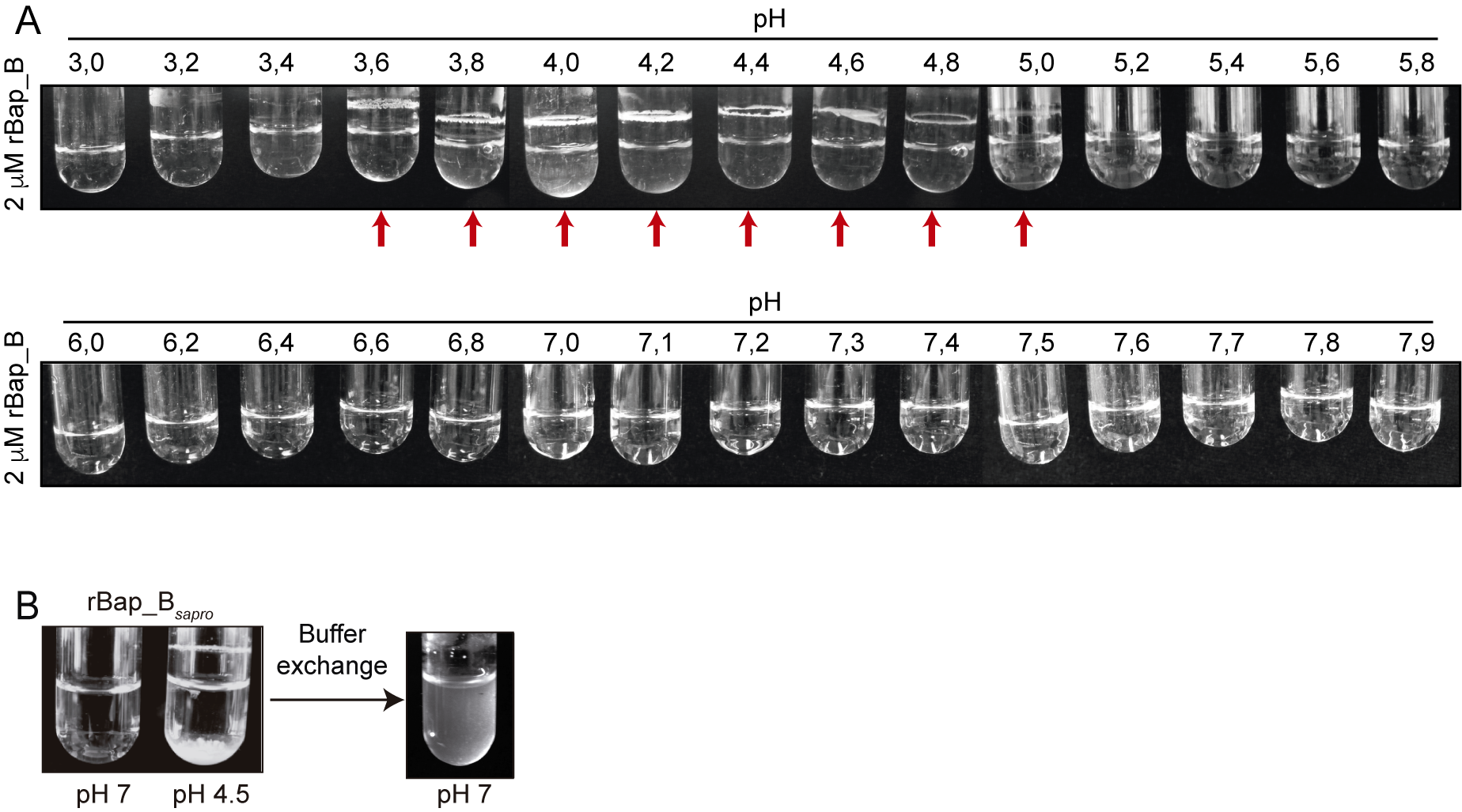

Supplement: S6 Fig — A) 2 μM of purified rBap_B protein was incubated in phosphate buffer solutions at different pH values. Aggregates were only visible in buffers with pH that fluctuated from 3.6 until 5 (indicated by a red arrow). B) rBap_Bsapro forms aggregates at pH 4.5. Reversion assay shows complete disassembly after phosphate-citrate buffer exchange from pH 4.5 to pH 7. (TIF) [file ppat.1005711.s006.tif]

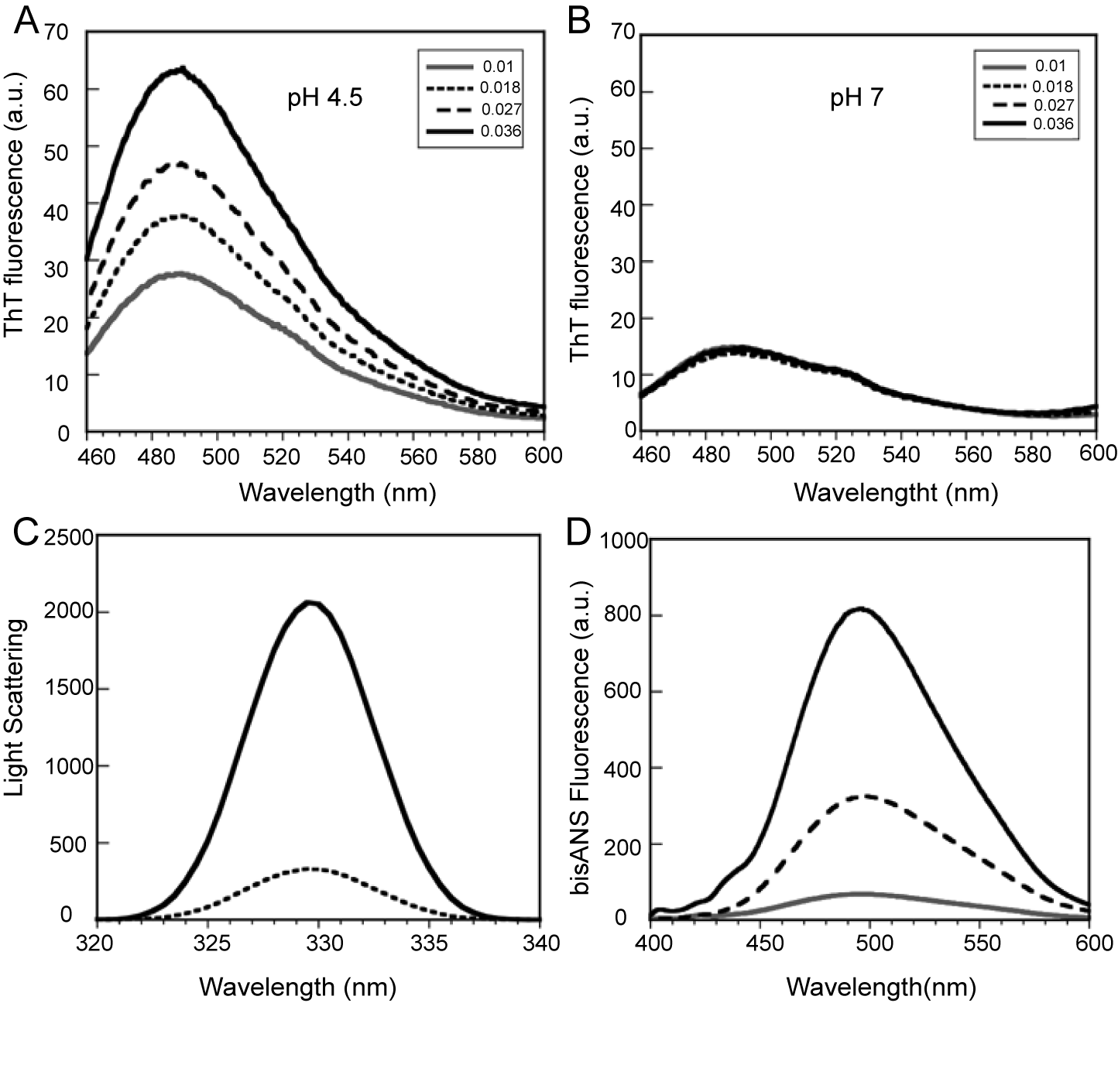

Supplement: S7 Fig — (A) Increase in ThT fluorescence emission upon binding to rBap_B aggregates at pH 4.5 and (B) pH 7. Free ThT emission spectrum is represented in grey. Data for Bap protein at 0.036, 0.027 and 0.018 mg/ml are represented as continuous, dashed and dotted black lines, respectively. (C) Static light scattering of 0.1 mg/ml rBap_B at pH 4.5 (solid line) and pH 7 (dashed line). (D) Bis-ANS fluorescence of 0.1 mg/ml rBap_B at pH 4.5 (solid line) and pH 7 (dashed line). Free bis-ANS is represented in grey. (TIF) [file ppat.1005711.s007.tif]

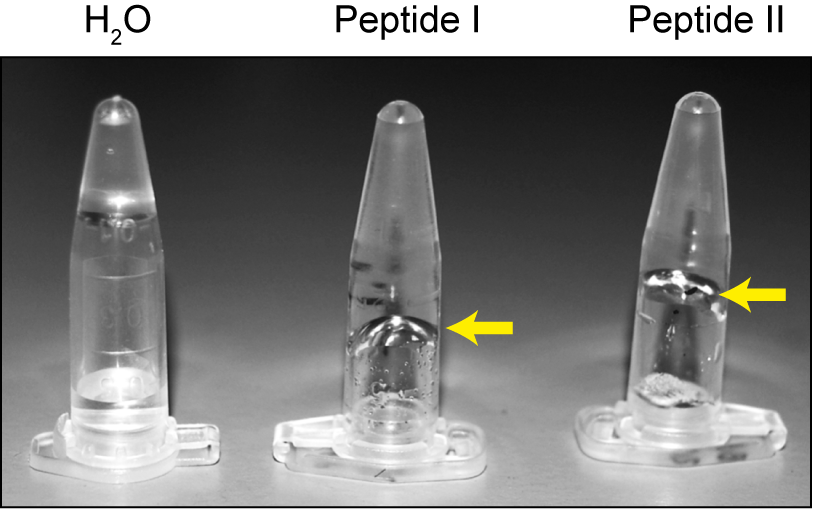

Supplement: S8 Fig — (TIF) [file ppat.1005711.s008.tif]

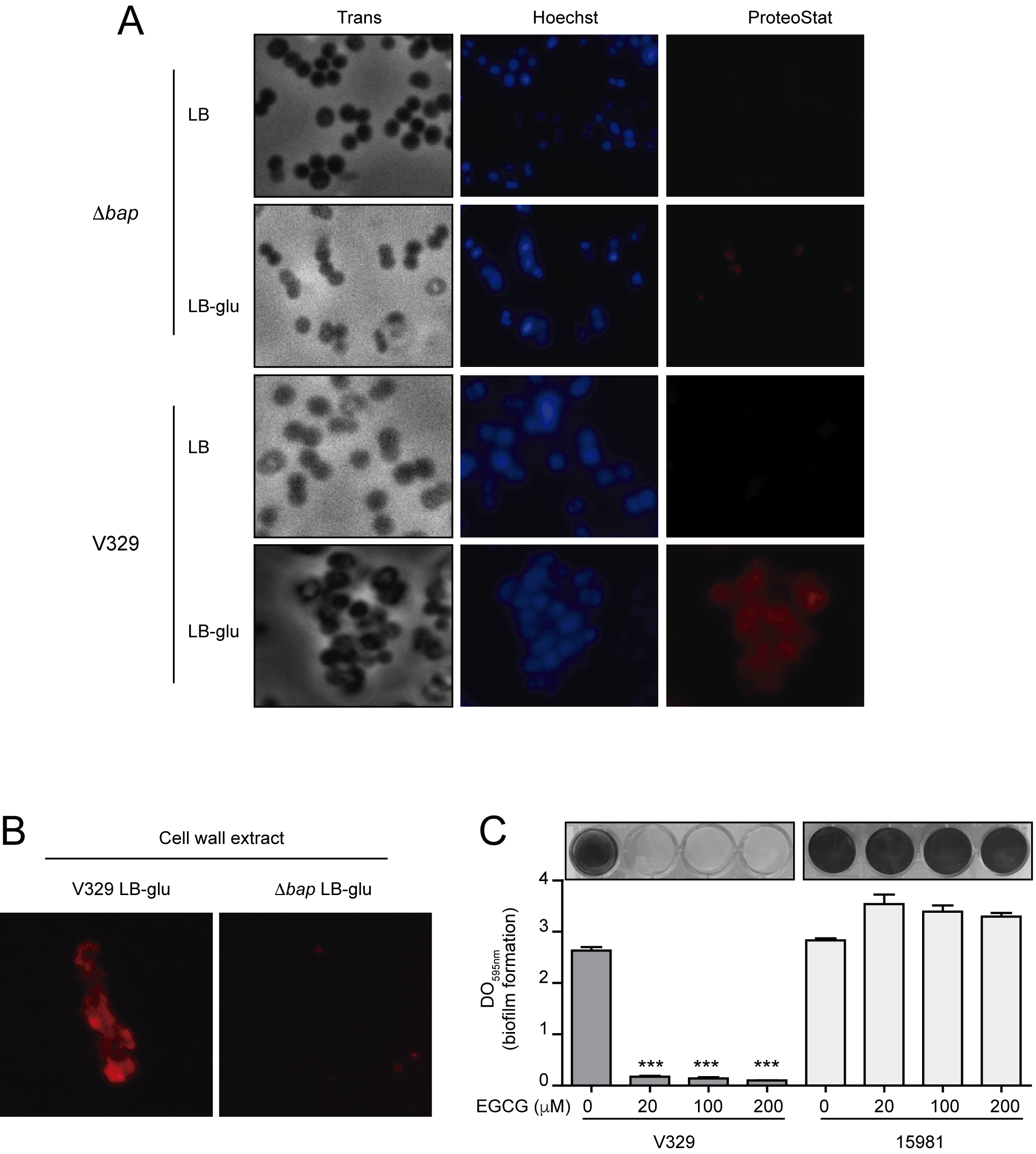

Supplement: S9 Fig — A) Biofilms from S. aureus V329 and Δbap strains grown in LB and LB-glu were stained with ProteoStat for 30 minutes. Representative fluorescence microscopic images are shown. B) Insoluble material retained in the native gel pocket from S. aureus V329 and Δbap strains cultured in LB-glu were extracted from the gel and stained with ProteoStat. C) After an overnight incubation in LB-glu at 37°C, S. aureus V329 biofilm disassembly in the absence or presence of 20, 100 and 200 μM EGCG was tested. S. aureus 15981 was used as a control and showed no significant differences of biofilm formation in the presence or absence of ECGC. Bars represent the mean values from five independent experiments (***, P<0.001). Statistical analysis was performed using the Mann-Whitney test. (TIF) [file ppat.1005711.s009.tif]

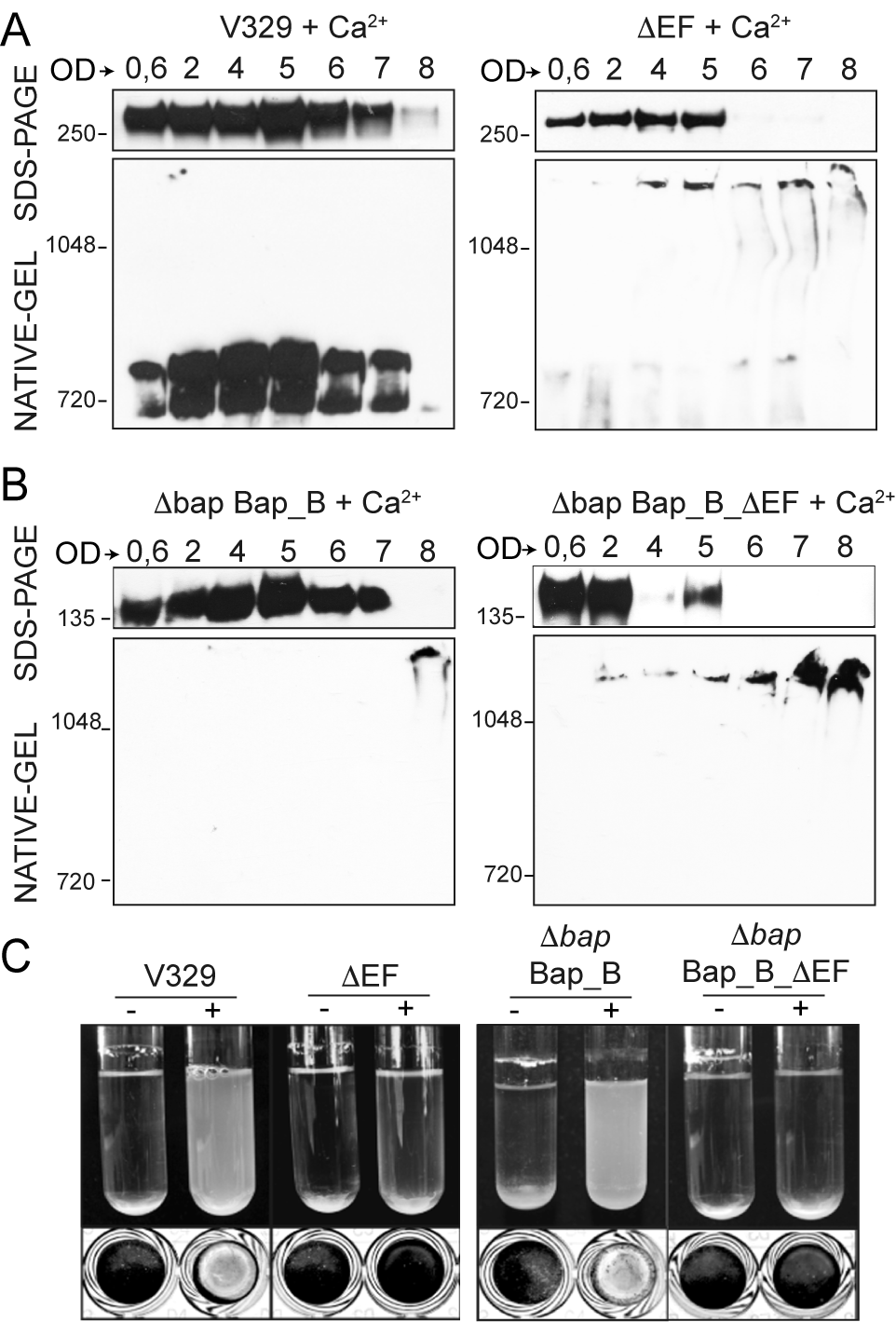

Supplement: S10 Fig — A) Immunoblot of cell wall extracts from S. aureus V329 and S. aureus ΔEF-hand and B) Δbap mutant expressing Bap_B chimeric protein and Δbap expressing Bap_B_ΔEF. Bacteria were grown in LB-glu supplemented with 20 mM CaCl2. Proteins were separated on 7.5% acrylamide gels (upper panel) or Criterion XT Tris-acetate gels with Tris/glycine running buffer (lower panel) and probed with anti-Bap antibodies. Size markers (in kDa) are indicated. C) Bacterial clumping and biofilm formation of S. aureus V329 and ΔEF mutant; Δbap expressing Bap_B and Δbap expressing Bap_B_ΔEF grown in LB-glu supplemented (+) or not (-) with 20 mM CaCl2. (TIF) [file ppat.1005711.s010.tif]

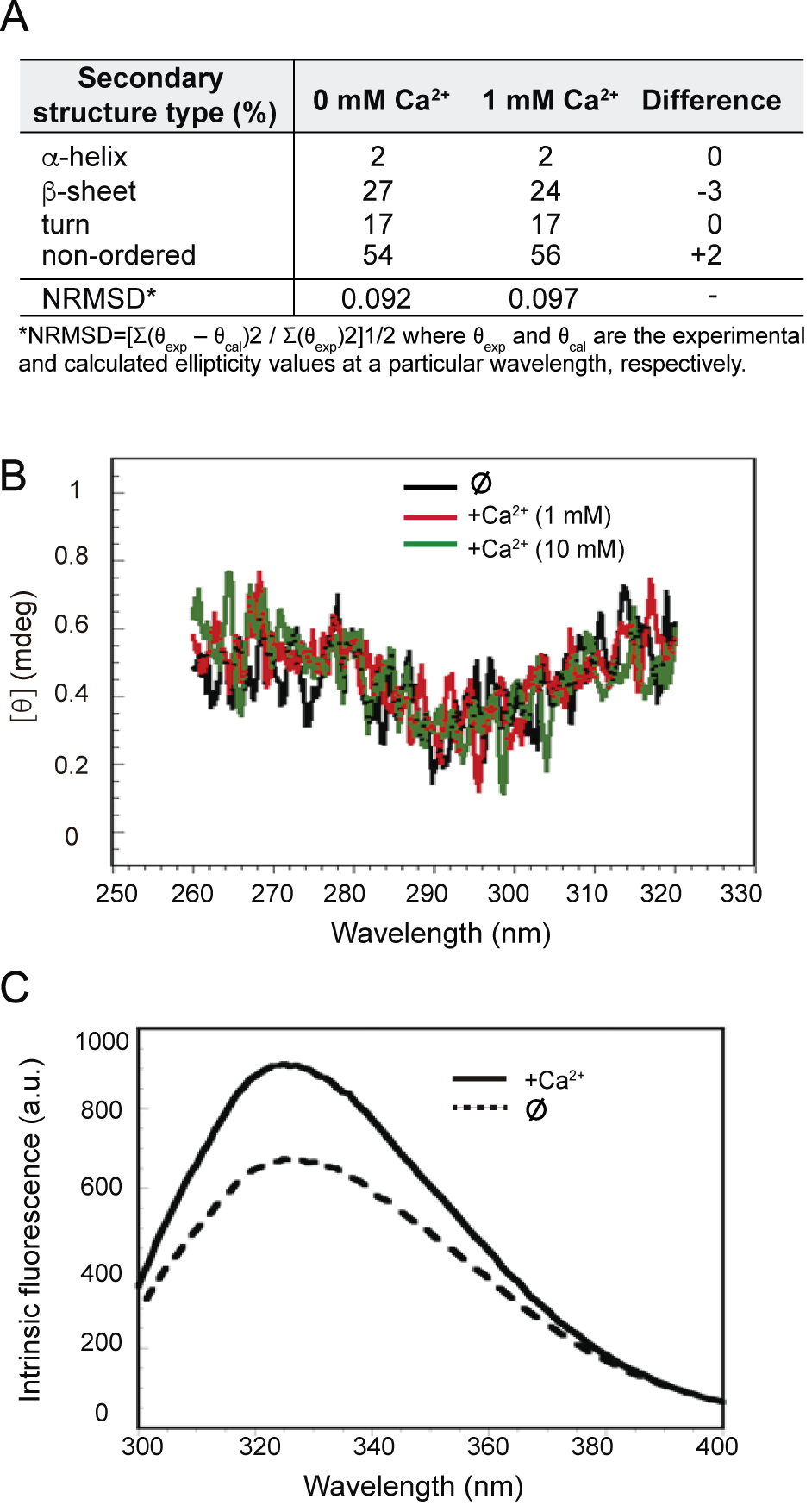

Supplement: S11 Fig — A) Effect of calcium in secondary structure composition of Bap determined by far-UV CD. Data were analyzed with Dichroweb implementing the CDSSTR algorithm. B) Near-UV CD spectra of 0.2 mg/ml rBap_B in the absence (black) or presence of 1 mM (red) and 10 mM (green) CaCl2. C) Intrinsic fluorescence of 1.5 mg/ml rBap_B at pH 7 in the presence (solid line) or absence (dashed line) of 100 mM CaCl2. (TIF) [file ppat.1005711.s011.tif]

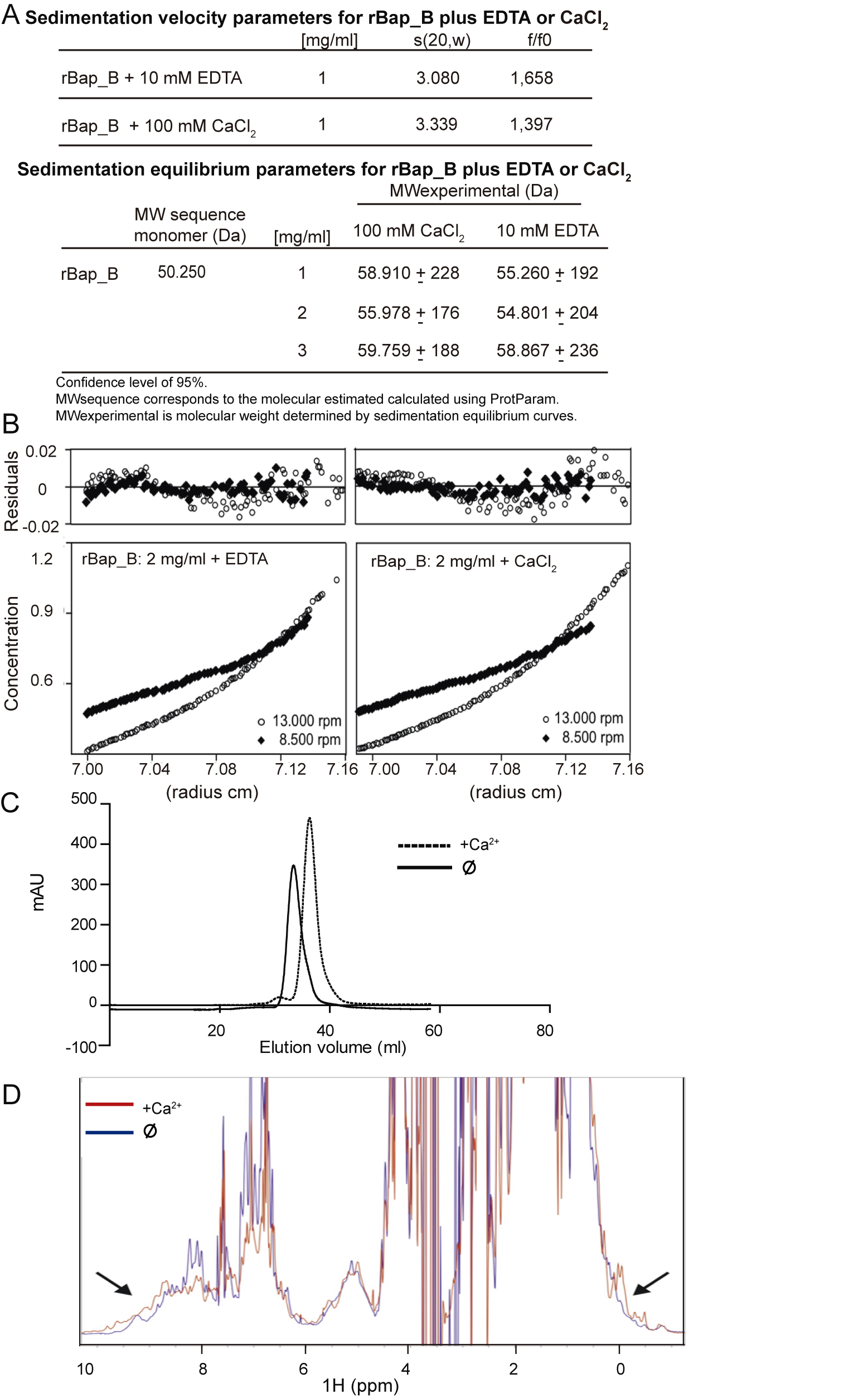

Supplement: S12 Fig — A) Summary of sedimentation velocity and sedimentation equilibrium parameters of rBap_B at pH 7 and in the presence or absence of calcium confirms monomeric state of the protein in both conditions. B) Sedimentation equilibrium analysis of 2 mg/ml rBap_B in the presence or absence of CaCl2, and at different rpm. Dotted lines show global fits. Residuals are shown above. C) Size exclusion chromatography of rBap_B. Chromatograms showing the protein eluted from a HiLoad 16/600 Superdex 200 pg column in buffers in the presence (dashed line) and absence (solid line) of 100 mM CaCl2. D) 1D 1H nuclear magnetic resonance (NMR) spectrum of 350 μM Bap_B in 10 mM MOPS, 100 mM CaCl2 (red) overlaid on that for 350 μM Bap_B in 10 mM MOPS, 10 mM EDTA (blue). The methyl region of the NMR spectrum includes high-field proton resonances observed at low chemical shifts (<0.5 ppm), which indicate the presence of characteristic clusters of aromatic and methyl groups in the core of a structured protein. In addition, the envelope of peaks resonating at high chemical shift (>8.5 ppm) correspond to highly ordered backbone amides present in secondary structure elements. The increase in the number of peaks within the fingerprint methyl and amide regions (arrowed) in the presence of Calcium indicate binding and induced structure in the Bap_B region. (TIF) [file ppat.1005711.s012.tif]

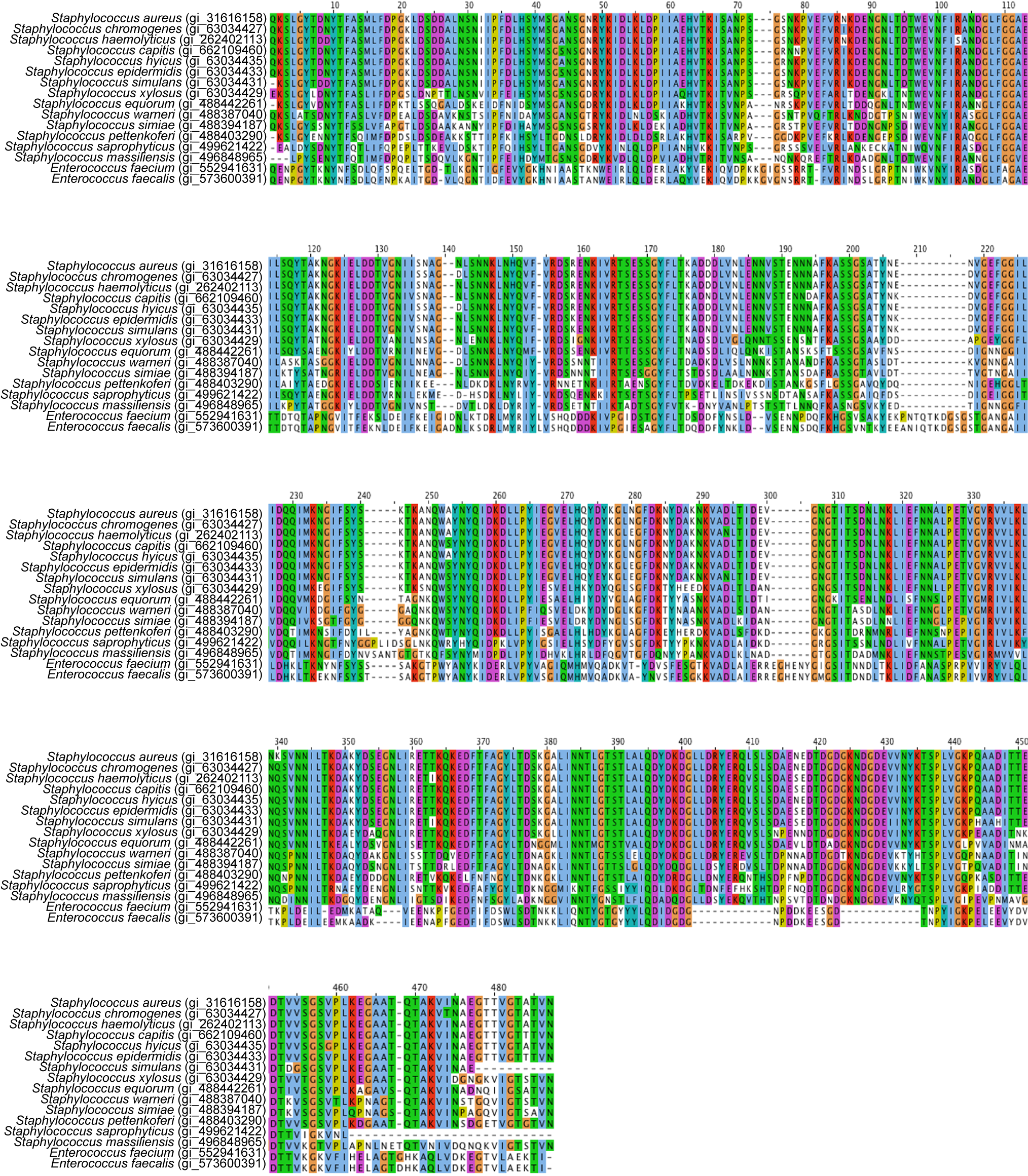

Supplement: S13 Fig — The alignment between regions B of Bap from 16 strains was generated using ClustalW2 multiple sequence alignment tool through Jalview program. GeneBank accession numbers are shown in brackets for each strain. (TIF) [file ppat.1005711.s013.tif]

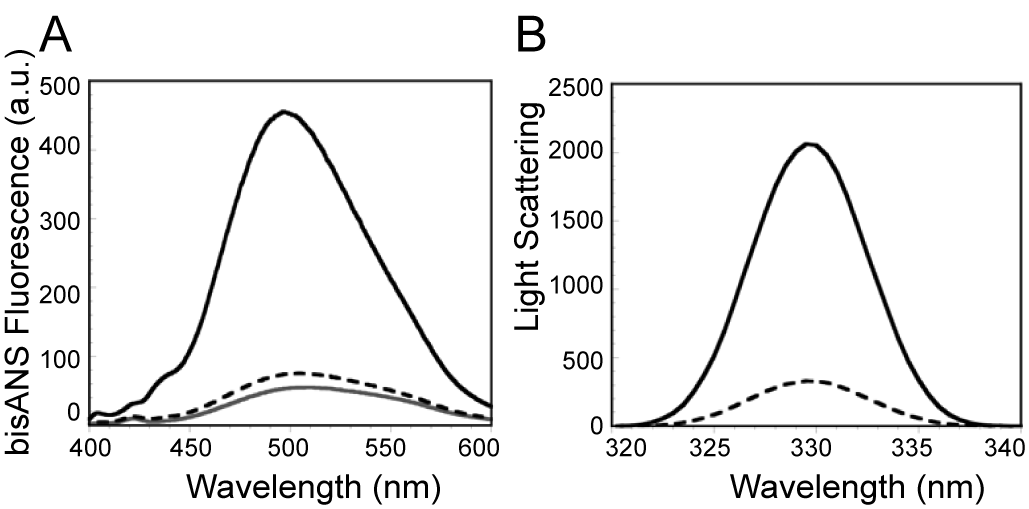

Supplement: S14 Fig — (A) Bis-ANS fluorescence and (B) static light scattering of 0.1 mg/ml rBap_Bsapro at pH 4.5 (solid line) and pH 7 (dashed line). Free bis-ANS is represented in grey. (TIF) [file ppat.1005711.s014.tif]

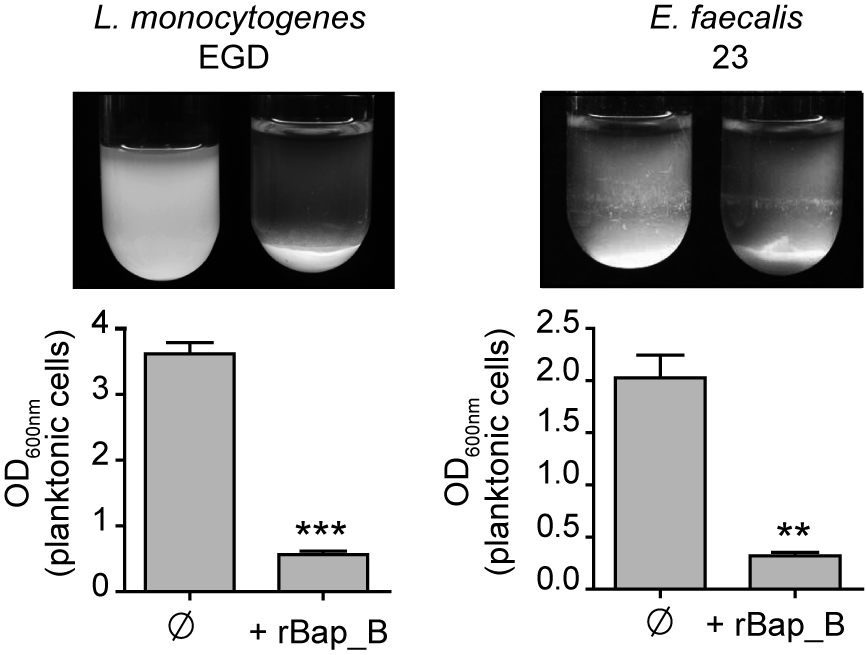

Supplement: S15 Fig — Bacterial clumping of L. monocytogenes EGD and E. faecalis 23 was evaluated after an overnight incubation at 37°C, 200 rpm, in the presence of 2 μM rBap_B protein. Data represent the means from three independent experiments. Error bars represent standard deviation (**, P<0.01; ***, P<0.001). Statistical analysis was performed using the unpaired Student t test. (TIF) [file ppat.1005711.s015.tif]
