# Supplementary material for: Staphylococcal Bap Proteins Build Amyloid Scaffold Biofilm Matrices in Response to Environmental Signals
Source: PLoS Pathog. 2016 Jun 21;12(6):e1005711. doi: 10.1371/journal.ppat.1005711 (PMC4915627; doi:10.1371/journal.ppat.1005711)
Supplement: S1 Table — (PDF) [file ppat.1005711.s016.pdf]

Table S1.

Strain/plasmid	Description	Reference or source[a]
Strains		
Staphylococcus aureus		
V329	Expresses Bap. Biofilm positive.	[1]
Δ*aur*	V329 with deletion of *aur* gene coding for aureolysin	This study
Δ*sspA*	V329 with deletion of *sspA* gene coding for serine protease SspA	This study
Δ*sspB*	V329 with deletion of *sspA* gene coding for cystein protease SspB	This study
ΔEF 2-3	V329 mutated in its EF-hand 2-3 motifs	[2]
Newman	Biofilm-negative strain.	[3]
MW2	Biofilm-negative strain.	[4]
Newman Bap_B	Newman complemented with pCN51:*bap_B*	This study
MW2 Bap_B	MW2 complemented with pCN51:*bap_B*	This study
Δ*bap*	V329 with deletion in *bap* gene.	[5]
Δ*spa*	V329 with deletion in *spa* gene	This study
Δ*bap*Δ*spa*	V329 with deletion in *bap* and *spa* genes	This study
Δ*bap*Δ*spa* pCN51	Δ*bap*Δ*spa* complemented with pCN51	This study
Δ*bap*Δ*spa* Bap_AB	Δ*bap*Δ*spa* complemented with pCN51:*bap_AB*	This study
Δ*bap*Δ*spa* Bap_B	Δ*bap*Δ*spa* complemented with pCN51:*bap_B*	This study
Δ*bap*Δ*spa* Bap_A	Δ*bap*Δ*spa* complemented with pCN51:*bap_A*	This study
Δ*bap*Δ*spa* ClfA	Δ*bap*Δ*spa* complemented with pCN51:*clfA*	This study
Δ*bap*Δ*spa* Bap_B ΔEF	Δ*bap*Δ*spa* complemented with pCN51:*bap_B ΔEF*	This study
Δ*bap*Δ*spa* Bap_B_{sapro}	Δ*bap*Δ*spa* complemented with pCN51:*bap_B* from *S. saprophyticus* B20080011225	This study
Δ*bap*Δ*spa Bap_B_*Δ*pepI_II*	Δ*bap*Δ*spa* complemented with pCN51:*bap_B containing mutations in amyloid peptides I and II*	This study
10833 Δ*ica*_Δ*tagO*	10833 with deletions of the *tagO* gene and the *ica* operon	[6]
10833 Δ*ica*_Δ*tagO Bap_B*	10833 with deletions of the *tagO* gene and the *ica* operon complemented with pCN51:*bap_B*	This study
Staphylococcus carnosus		
TM300	Biofilm negative	[7]
Bap_B	*S. carnosus* TM300 complemented with pCN51:*bap_B*	This study
Staphylococcus saprophyticus		
B20080011225	Clinical strain	CUN[a]
Staphylococcus simiae		
CCM 7213	Squirrel monkey isolate	[8]
Staphylococcus epidermidis		
C533	*bap*-positive strain	[9]
Staphylococcus xylosus		
C482	*bap*-positive strain	[9]
Staphylococcus simulans		
ATCC 1362	*bap*-positive strain	[9]
Escherichia coli		
XL1-Blue	Used for cloning assays	
BL21(DE3) pET46-ek/LIC:*bap_B*	Expresses B region of Bap from *S. aureus* V329	This study
BL21(DE3) pET46-eK/LIC:*bap_Bsapro*	Expresses B region of Bap from *S. saprophyticus* B20080011225	This study
VS39	Δ(*csgABC*) + pVS76 (produces CsgG)	[10]
VS39 Bap_B_{aur}	VS39 complemented with pEXPORT:bap_B (*S. aureus)*	This study
VS39 Bap_A_{aur}	VS39 complemented with pEXPORT:bap_A (*S. aureus)*	This study
VS39 Bap_B_{sapro}	VS39 complemented with pEXPORT:bap_B (*S. saprophyticus)*	This study
VS39 Bap_B_{simiae}	VS39 complemented with pEXPORT:bap_B (*S. simiae)*	This study
VS39 Bap_B_{epid}	VS39 complemented with pEXPORT:bap_B (*S. epidermidis)*	This study
VS39 Bap_$B_{xylosus}$	VS39 complemented with pEXPORT:bap_B (*S. xylosus)*	This study
Plasmids		
pJET1.2/blunt	Cloning vector	Termo Scientific
pCN51	*E. coli*-S. *aureus* shuttle vector with a cadmium inducible promoter.	[11]
pET46-ek/LIC	Expression vector.	Novagen
pMAD	Vector for recombination experiments.	[12]
pEXPORT	Expression vector for C-DAG system (NotI-XbaI restriction sites for insertion of gene of interest)	[10]
$pEXPORT_{XhoI}$	Expression vector for C-DAG system (NotI-XhoI restriction sites for insertion of gene of interest)	This study

[a] CUN, Clínica Universitaria de Navarra (Pamplona, Spain)
