# Supplementary material for: Staphylococcal Bap Proteins Build Amyloid Scaffold Biofilm Matrices in Response to Environmental Signals
Source: PLoS Pathog. 2016 Jun 21;12(6):e1005711. doi: 10.1371/journal.ppat.1005711 (PMC4915627; doi:10.1371/journal.ppat.1005711)
Supplement: S2 Table — (PDF) [file ppat.1005711.s017.pdf]

S2 Table

Oligonucleotide	Sequence
bapori-1mB	GGATCCTTTATTTTGAGGTGAGTAAATATGGG
bap-63cK	GGTACCGGTGCCTTCTGGTGAATTTGG
bap-65c	GGCTCTTTAATTGAATTAGATGAAGCACTATTTTGTACTTCCGC
bap-66m	GCGGAAGTACAAAATAGTGCTTCATCTAATTCAATTAAAGAGCC
bap-B1	GCCATCAGTATCTTCGTTTTCAGCAATAATTGGATCTAAT
bap-B2	ATTAGATCCAATTATTGCTGAAAACGAAGATACTGATGGC
bap-B3K	GGTACCATTTACAGTTGCTGTACC
K-3xF-clfA	GGTACCgactacaaagaccatgacggtgattataaagatcatgacatcgactacaaggatgacgatgacaagGAAATTGAACCAATTCCAGAGGAT
clfA-7cE	GAATTCTTACACCCTATTTTTTCGCC
clfA-9mB	GGATCCCTTTTTAAAAAGAGGGAATAAAATGAATATG
SPbap-sapro-Rv	TTGTCAGAATAATCCAATGCTTCGTCTAATGCTTCACCTTTGTC
bapB-sapro-Fw	GACAAAGGTGAAGCATTAGACGAAGCATTGGATTATTCTGACAA
Sapbap-KpnI-Rv	GGTACCTGATTTATGAAATTCAAAGTTATCT
spaE	GATGATGTATACAATGTATTTC
spaF	TGCGTCTCGATTTAATTGG
Aur-FW	CTTCATGTTCACAATAGTGTCTAAAC
Aur-11mE	ACAAAGGAAGCTGATAGCGG
ssp-20cN	TTGAACTTGATCTTCTTGTATCGC
ssp-17ms	ATTACCGCTCAACCTGAAGGC
ssp-24cN	GGTGAAGACCAAATCCCTCGC
ssp-21mS	ACGAATGGTCATTATGCACCC
bapB1-LIC-Fw	GACGACGACAAGATGCAAAAATCTTTAGGTTACACAGATAATTATAC
bapB1-LIC-Rv	GAGGAGAAGCCCGGTTAATTTACAGTTGCTGTACCAACTGTTGTAC
bapB-sapro-LIC-Fw	GACGACGACAAGATGGAAGCATTGGATTATTCTGACAA
bapB-sapro-LIC-Rv	GAGGAGAAGCCCGGTTATAAATTTACTTTACCAATTACTGTAGTATC
pVS72-XhoI-5	CCAAGCTTGCATGCCTGCAGGTCGACCTCGAGTTAGTGATGATGGTGATGGTGATCGTTA
pVS72-XhoI-3	TAACGATCACCATCACCATCATCACTAACTCGAGGTCGACCTGCAGGCATGCAAGCTTGG
cdag_B_NotI_Fw	GCGGCCGCACAAAAATCTTTAGGTTACACAGATAATTATAC
cadg_B_XhoI_Rv	CTCGAGTTAGTGATGATGGTGATGGTGATTTACAGTTGCTGTACCAACTGTTGTAC
CDAG BAP_A-Fw	GCGGCCGCATCAGAAAATTCCAATGACACTGCT
CDAG BAP_A-Rv	CTCGAGTTAGTGATGATGGTGATGGTGTTCTAATTCAGATTCTTCATTTTTATCAGT
BAPsapro_cdag_Fw	GCGGCCGCAGAAGCATTGGATTATTCTGACAA
BAPsapro_cdag_rv	CTCGAGTTAGTGATGATGGTGATGGTGTAAATTTACTTTACCAATTACTGTAGTATC
BAPsimiae_cdag_Fw	GCGGCCGCACAAAAATCATTAGGTTATTCAAGTAACT
BAPsimiae_cdag_Rv	CTCGAGTTAGTGATGATGGTGATGGTGATTAACAGCTGATGTACCTATCA
epider_CDAG_Fw	GCGGCCGCACAAAAATCTTTAGGTTACACAGAT
epider_CDAG_Rv	CTCGAGTTAGTGGTGATGGTGATGATGATTTACAGTTGTTGTACCAACT
warneri_CDAG_Fw	GCGGCCGCACAAAAATCTTTAGCAACATCAG
warneri_CDAG_Rv	CTCGAGTTAGTGGTGATGGTGATGATGATTAACAGTAGACGTACCAATAAC
xylosus_CDAG_Fw	GCGGCCGCAGAAAAATCATTAGGTTACTTAGATAATT
xylosus_CDAG_Rv	CTCGAGTTAGTGGTGATGGTGATGATGGTTAACTGTAGATGTTCCAATCA
simulans_CDAG_Fw	GCGGCCGCAAAATCTTTAGGTTACACAGATGA
simulans_CDAG_Rv	CTCGAGTTAGTGGTGATGGTGATGATGCTCTGCATTAATTACTTTAGCT

[a] Restriction sites of enzymes are underlined. 3xFlag sequence is in small letters.
