# Supplementary material for: Staphylococcal Bap Proteins Build Amyloid Scaffold Biofilm Matrices in Response to Environmental Signals
Source: PLoS Pathog. 2016 Jun 21;12(6):e1005711. doi: 10.1371/journal.ppat.1005711 (PMC4915627; doi:10.1371/journal.ppat.1005711)
Supplement: S3 Table — (PDF) [file ppat.1005711.s018.pdf]

S3 Table . Summary of the proteins identified by proteomics from aggregates of *S. aureus* V329 in LB-glu and LB.

		LB-glu			LB		
Putative Protein	NCBI GI nº	Total score	% coverage	Peptides (>95%)	Total score	% coverage	Peptides (>95%)
Biofilm-associated protein	31616158	89,13	53,3	53	--	--	--
Bifunctional autolysin precursor	151221134	50,48	53,6	26	41,98	62	20
Hypothetical protein	151220813	33,77	70,2	28	45,82	78,3	33
branched-chain alpha-keto acid dehydrogenase subunit E2	151221173	31,49	64,7	18	24,37	54,9	14
Dihydrolipoamide dehydrogenase	151221174	29,13	68,6	22	22,48	68,2	13
ABC trasporter, amino acid binding protein	151222525	26,25	75,7	15	11,96	69,1	8
50s ribosomal protein L2	151222361	21,97	69,7	12	1,27	37,6	1
Penicillin binding protein 2	151221573	20,29	44,3	12	24,9	68,8	13
Pyruvate kinase	151221804	18,53	58,5	11	--	--	--
Molybdate binding protein	151222391	18,31	65,8	9	10,89	74,2	8
Elongation factor Tu	151220722	17,33	64,7	9	8,82	61,9	6
peptidyl-propyl cis/trans-isomerase	151221945	15,72	66,6	9	14,85	74,4	9
ABC transporter substrate-binding protein	151220994	15,2	67,4	7	22,25	70,7	14
Pyruvate dehydrogenase E1	151221172	13,41	46,8	7	12,71	71,1	7
Hypothetical protein	151220899	12,99	52,9	7	5,95	93,9	3
malate:quinone oxidoreductase	151222716	12,38	37,4	8	2,46	34,4	1
Glyceraldehyde-3-phosphate dehydrogenase	151220953	12,15	67	7	2,1	56,9	1
Signal peptidase IB	151221048	12,01	67,5	7	10,74	63,4	5
Immunoglobulin-binding protein (Sbi)	151222529	10,96	69	4	9,88	57,1	5
50s ribosomal protein L15	151222345	10,38	82,2	5	--	--	--
phosphopyruvate hydratase	151220957	10,09	48,6	5	--	--	--
50s ribosomal protein L21	151221761	9,59	96,1	6	3,72	68,6	2
N-acetylmuramoyl-L-alanine amidase	151222755	8,76	54,9	4	--	--	--
Hypothetical protein	151221023	8,5	32,8	4	--	--	--
Bifunctional preprotein translocase subunit SecD/SecF	151221751	8	30,6	4	1,96	30,2	1
Universal stress protein	151221816	7,95	62,1	4	7,44	76,5	5
Hypothetical protein	151220965	7,43	59,5	4	5,73	50	3
Secretory antigen precursor SsaA	151222411	7,01	34,8	7	0,37	3,7	1
Hypothetical protein	151220889	6,47	52,7	4	--	--	--
30s ribosomal protein S13	151222340	6,17	53,7	3	1,96	79,3	1
30s ribosomal protein S9	151222331	6,1	59,2	4	--	--	--
L-lactate dehydrogenase	151220388	5,64	20,2	3	--	--	--
elongation factor G	151220721	5,57	25,5	4	--	--	--
30S ribosomal protein S4	151221825	5,5	41	3	2,61	39,5	2
30S ribosomal protein S2	151221378	5,48	33,3	3	2	60,4	2
50S ribosomal protein L17	151222337	5,43	63,9	3	--	--	--
50S ribosomal protein L20	151221784	5,35	83,1	4	2,99	83,1	2
Hypothetical protein	151221170	5,08	52,4	2	2	50,5	2
50S ribosomal protein L19	151221363	5,06	76,7	3	1,09	50	1
Cell envelope-related transcriptional attenuator	151222423	4,53	26,7	3	2,12	37,8	2
Hypothetical protein	151221647	4,5	54,4	3	2,29	53,9	2
Immunodominant antigen B	151222749	4,41	57,7	3	11,24	66,9	8
pyruvate dehydrogenase E1 component, alpha subunit	151221171	4,38	49,2	2	4,69	49,2	2
acetolactate synthase	151222323	4,31	31,4	3	--	--	--
ABC transporter iron compound-binding protein	151222143	4,24	55,8	2	4,05	58,1	2
30S ribosomal protein S7	151220720	4,24	41,7	2	2,21	78,2	2
Hypothetical protein	151222466	4,21	36,7	2	2,92	40,5	2
mannosyl-glycoprotein endo-beta-N-acetylglucosamidase	151221879	4,18	33,1	2	1,64	53,9	1
Immunodominant antigen A	151222681	4,02	32,6	3	2,05	21,5	1
30S ribosomal protein S10	151222365	4	72,6	2	2,04	51	1
ferrichrome ABC transporter lipoprotein	151222290	4	24,2	2	8,63	55,7	4
50S ribosomal protein L16	151222357	4	46,5	2	2,44	58,3	2
Hypothetical protein	151220576	3,96	31,1	2	7,97	78,4	5
30S ribosomal protein S8	151222350	3,84	60,6	3	1,37	64,4	1
50S ribosomal protein L5	151222352	3,76	52	3	--	--	--
50S ribosomal protein L4	151222363	3,72	36,2	3	--	--	--
quinol oxidase polypeptide II QoxA	151221142	3,45	49,7	3	16,6	62	9
50S ribosomal protein L29	151222356	3,34	43,8	2	--	--	--
50S ribosomal protein L14	151222354	3,28	32,8	2	--	--	--
alcohol dehydrogenase	151220789	3	17	2	--	--	--

Common proteins identified in both LB and LB-glu conditions are highlighted in yellow.
