# Supplementary material for: Staphylococcal Bap Proteins Build Amyloid Scaffold Biofilm Matrices in Response to Environmental Signals
Source: PLoS Pathog. 2016 Jun 21;12(6):e1005711. doi: 10.1371/journal.ppat.1005711 (PMC4915627; doi:10.1371/journal.ppat.1005711)
Supplement: S4 Table — Data were analyzed with Dichroweb implementing the CDSSTR algorithm. *NRMSD = [Σ(θexp– θcal)2 / Σ(θexp)2]1/2 where θexp and θcal are the experimental and calculated ellipticity values at a particular wavelength, respectively. (PDF) [file ppat.1005711.s019.pdf]

S4 Table

Secondary structure type	pH 7.0	pH 4.4	Difference
α-helix (%)	2	2	0
β-sheet (%)	26	31	+5
turn (%)	17	16	-1
nonordered (%)	55	51	-4
NRMSD*	0.094	0.090	-
