# Supplementary material for: Staphylococcal Bap Proteins Build Amyloid Scaffold Biofilm Matrices in Response to Environmental Signals
Source: PLoS Pathog. 2016 Jun 21;12(6):e1005711. doi: 10.1371/journal.ppat.1005711 (PMC4915627; doi:10.1371/journal.ppat.1005711)
Supplement: S5 Table — (PDF) [file ppat.1005711.s020.pdf]

S5 Table . Sequence comparison of the B region of Bap between different staphylococcal species.

	Protein identity (%)	Protein similarity (%)	Theoretical pI [a]	EF-hand motifs [b]		
				aa	sequence	similarity
S. aureus	100	–	4.61	729-741<br>752-764	DYDKDGLLDRYEr<br>DTDGDGKnDGDEV	89 %<br>91 %
S. epidermidis	97	99	4.56	729-741<br>752-764	DYDKDGLLDRYEr<br>DTDGDGKnDGDEV	91 %<br>98 %
S. simulans	95	98	4.65	729-741<br>752-764	DYDKDGLLDRYEr<br>DTDGDGKnDGDEI	91 %<br>89 %
S. xylosus	80	91	4.52	725-737<br>748-760	DYDKDGLLDRYEr<br>DTDGDGKnDGDEV	91 %<br>89 %
S. simiae	68	80	4.43	612-624<br>651-663<br>743-755<br>766-778	DKDlipFIESVEL<br>DANGNGTITASDL<br>DYDQDGLLDSYEr<br>DTDGDGKnDGDEV	80 %<br>Identity<br>91 %<br>89 %
S. saprophyticus	58	74	4.74	557-569<br>580-592	DLDKDGLtDNFEF<br>DTDGDGKnDGDEV	89 %<br>89 %

[a] Theoretical pI corresponds to the estimated isoelectric point calculated using Compute pI/Mw tool of ExPaSy server.

[b] EF- hand motifs corresponds to the predicted EF-hand-like calcium binding domains calculated using PROSCAN (PROSITE database). Residues that do not conform the EF- hand loop consensus sequence are in lowercase letters.
