# Supplementary material for: Staphylococcal Bap Proteins Build Amyloid Scaffold Biofilm Matrices in Response to Environmental Signals
Source: PLoS Pathog. 2016 Jun 21;12(6):e1005711. doi: 10.1371/journal.ppat.1005711 (PMC4915627; doi:10.1371/journal.ppat.1005711)
Supplement: S1 References — (PDF) [file ppat.1005711.s021.pdf]

S1. References

1. Cucarella C, Solano C, Valle J, Amorena B, Lasa I, Penades JR. Bap, a *Staphylococcus aureus* surface protein involved in biofilm formation. J Bacteriol. 2001;183: 2888–2896.

2. Arrizubieta MJ, Toledo-Arana A, Amorena B, Penadés JR, Lasa I. Calcium inhibits Bap-dependent multicellular behavior in *Staphylococcus aureus*. J Bacteriol. 2004;186: 7490–7498.

3. Duthie ES, Lorenz LL. Staphylococcal coagulase: mode of action and antigenicity. J Gen Microbiol. 1952;6: 95–107.

4. Baba T, Bae T, Schneewind O, Takeuchi F, Hiramatsu K. Genome sequence of *Staphylococcus aureus* strain Newman and comparative analysis of staphylococcal genomes: polymorphism and evolution of two major pathogenicity islands. 2008;190: 300–310.

5. Valle J, Latasa C, Gil C, Toledo-Arana A, Solano C, Penadés JR, et al. Bap, a biofilm matrix protein of *Staphylococcus aureus* prevents cellular internalization through binding to GP96 host receptor. PLoS Pathog. 2012;8: e1002843

6. Vergara-Irigaray M, Maira-Litrán T, Merino N, Pier GB, Penadés JR, Lasa I. Wall teichoic acids are dispensable for anchoring the PNAG exopolysaccharide to the *Staphylococcus aureus* cell surface. Microbiology. 2008;154: 865–877.

7. Rosenstein R, Nerz C, Biswas L, Resch A, Raddatz G, Schuster SC, et al. Genome analysis of the meat starter culture bacterium *Staphylococcus carnosus* TM300. Appl Environ Microbiol. 2009;75: 811–822.

8. Pantůcek R, Sedlácek I, Petrás P, Koukalová D, Svec P, Stetina V, et al. *Staphylococcus simiae* sp. nov., isolated from South American squirrel monkeys. Int J Syst Evol Microbiol. 2005;55: 1953–1958.

9. Tormo MA, Knecht E, Götz F, Lasa I, Penadés JR. Bap-dependent biofilm formation by pathogenic species of *Staphylococcus*: evidence of horizontal gene transfer? Microbiology. 2005;151: 2465–247

10. Sivanathan V, Hochschild A. A bacterial export system for generating extracellular amyloid aggregates. Nat Protoc. 2013;8: 1381–1390.

11. Charpentier E, Anton AI, Barry P, Alfonso B, Fang Y, Novick RP. Novel cassette-based shuttle vector system for gram-positive bacteria. Appl Environ Microbiol. 2004;70: 6076–6085.

12. Arnaud M, Chastanet A, Débarbouillé M. New vector for efficient allelic replacement in naturally nontransformable, low-GC-content, gram-positive bacteria. Appl Environ Microbiol. 2004;70: 6887–6891.
